# Synthesis of Macrocyclic Structures by Intramolecular Photoclick Reaction

**DOI:** 10.1021/jacs.6c09335

**Published:** 2026-07-22

**Authors:** Shubham Sharma, Patrick Foster, Christopher Molnar, Ronaldo Badenhorst, Sergiy Minko, Vladimir V. Popik

**Affiliations:** † Department of Chemistry, 1355 University of Georgia, Athens, Georgia 30602, United States; ‡ J-STAR Research Inc., Proton Pharma, Cranbury, New Jersey 08512, United States; § University of NorthCarolina, Chapel Hill, North Carolina 27599, United States

## Abstract

The photo-SPAAC reaction allows for the efficient preparation of medium- to large-sized macrocycles. Photo-SPAAC cyclization of bifunctional linear molecules, which carry a cyclopropenone-caged dibenzocyclooctyne moiety at one terminus and an azide group at the other, leads to the formation of macrocycles in high to quantitative yields. Macrocycles with 18- to 51-membered ring sizes were prepared both in solution and in the neat state, indicating an unusually high effective molarity of bifunctional cyclooctyne–azide linear molecules. No dimers or higher oligomers were observed in the reaction mixtures. SPAAC cyclization pathways were analyzed at the DFT level of theory. Macrocycles with 17- to 34-membered ring sizes were also successfully prepared by the SPAAC-photo-SPAAC reaction sequence, where monobifunctional dibenzocyclooctadiyne or bis-dibenzocyclooctynes react with bis-azides. This method also produces macrocycles in high yield, albeit small amounts of dimeric cyclic byproducts were detected in some cases.

## Graphical abstract

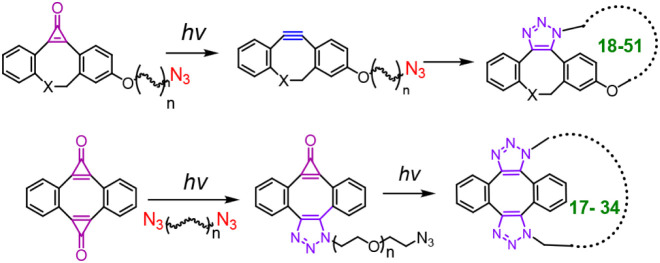

## Introduction

Macrocycles are large cyclic structures consisting of 12 or larger membered rings. Oftentimes, macrocycles are found in nature and exhibit unique biological activity, as highlighted by the FDA-approved agents, erythromycin and rapamycin.^1^ Macrocyclic peptides have attracted significant attention in recent years due to their improved proteolytic stability, cell permeability, and unique binding affinities, especially in protein–protein interactions.^2^ Beyond drug discovery,^3^ macrocycles can be found in supramolecular chemistry, where their unique preorganized cavities are leveraged to enable multivalent binding sites, with applications in molecular recognition and sensing.^4,5^ Despite their apparent utility, the design and synthesis of macrocycles remain an ongoing challenge.^6–9^ The main complication in the synthesis of macrocyclic structures from bifunctional precursor molecules is the competition between macrocyclization and dimerization or oligomerization.^10^ Since effective molarities (EMs) of most common reactions employed in these syntheses rarely exceed the millimolar level, the preparation of macrocycles requires very dilute conditions.^10,11^

Copper-catalyzed azide-alkyne cycloaddition (CuAAC) click chemistry has emerged as a useful macrocyclization strategy due to high selectivity, fast reaction rates, and mild conditions.^12^ Moreover, the resulting triazole ring formed upon CuAAC exhibits polar C–H and N–H bonds, which can enhance binding efficiency.^13^ On the other hand, the use of a copper catalyst can have a hampering effect on the reaction due to the affinity of copper to electron donors and multivalent binding sites in macrocycle precursors.^14^ The strain-promoted azide-alkyne cycloaddition (SPAAC) reaction offers a practical alternative to CuAAC for macrocycle syntheses as it does not require a metal catalyst. However, the preparation of a bifunctional molecule that contains both azide and cyclooctyne moieties is not feasible due to their high mutual reactivity. The alternative approach of coupling bis-azides with bis-cyclooctynes has been reported to produce a mixture of oligomeric and cyclic structures even at concentrations as high as 0.5 M,^15^ thus suggesting high EM for the SPAAC cyclization reaction.

Here, we report an efficient strategy for the preparation of macrocycles using the photo-SPAAC reaction.^16^ The bifunctional linear precursor contains an azide group on one terminus and a cyclopropenone-caged dibenzocyclooctyne (photo-DIBO (**1a**), X = CH_2_ or photo-ODIBO (**1b**), X = O; Scheme 1) on the other.

**Scheme 1:**
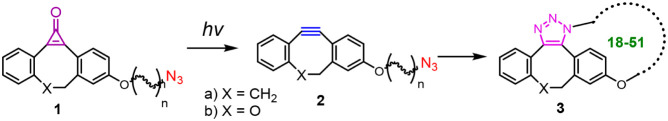
Photo-SPAAC Macrocyclization

Cyclopropenone-caged dibenzocyclooctynes (such as **1a** or **1b**) do not react with organic or inorganic azides and possess excellent thermal and hydrolytic stability. Upon UVA or blue light irradiation, as well as exposure to NIR pulsed laser emission, **1** undergoes efficient decarbonylation (Φ ∼ 0.3–0.4) to produce azide-reactive cyclooctynes (DIBO (**2a**) or ODIBO (**2b**)) in quantitative yield. The azide group then undergoes rapid intramolecular addition to the cyclooctyne moiety, producing macrocycle **3** in a good to quantitative yield (Scheme 1). The utility of the photo-SPAAC cyclization for the synthesis of macrocycles has been demonstrated by preparing 18- to 51-membered cycles (*vide infra*). From the photochemical point of view, the photo-SPAAC macrocyclization offers another convenient feature: irradiation of **1** with UVA light results in the bleaching of the 347 nm band of the photo-XDIBO moiety. Dibenzocyclooctyne **2** and triazole **3** have virtually no absorbance above 320 nm^16a,17^ and, therefore, do not interfere with the photolysis of **1,** even at high conversion.

## Results and Discussion

The general strategy for the preparation of macrocycle precursors **1** is illustrated by the example of the syntheses of photo-ODIBO-EG_4_-N_3_ (**6a**) and photo-ODIBO-EG_3_-N_3_ (**6b**, Scheme 2). Preparation of bifunctional precursor **6a** starts with Williamson’s etherification of photo-ODIBO-OH (**4**)^18^ with TBDMS-protected tetraethylene glycol tosylate to give photo-ODIBO-EG_4_-OTBDMS (**5a**). The removal of silyl protection of the terminal hydroxy group with hydrofluoric acid was followed by the treatment of the resulting **5b** with TosCl in the presence of triethylamine to give photo-ODIBO-EG_4_-OTos (**5c**). The reaction of **5c** with sodium azide in DMF produced the target photo-ODIBO-EG_4_-N_3_ (**6a**, Scheme 2). Photo-ODIBO-EG_3_-N_3_ (**6b**) was prepared by the direct Williamson etherification of **4** with TosO-EG_3_-N_3_. The same reaction using TosO-EG_4_-N_3_ produced **6a** in 15% yield. Details of the preparative procedures are presented in the Supporting Information.^19^

**Scheme 2:**
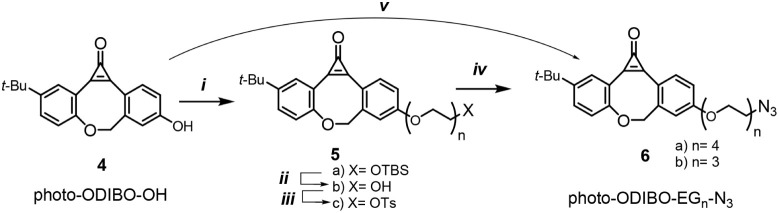
Synthesis of Photo-ODIBO-EG_4_-N_3_ (**6a**) and Synthesis of Photo-ODIBO-EG_3_-N_3_ (**6b**)­^a^

a Reagents and Conditions: (i) TBDMSO­(CH_2_CH_2_O)_4_Tos, K_2_CO_3_, DMF, 87%; (ii) HF_aq_, CHCl_3_ 89%; (iii) TosCl, TEA, DCM, 86%; (iv) NaN_3_, DMF, 57%; (v) Tos­(OCH_2_CH_2_)_n_N_3_, K_2_CO_3_, DMF, 6a 15%, 6b 40%

### Light-Induced Cyclization of Photo-ODIBO-EG_4_-N_3_ (**6a**) and Photo-ODIBO-EG_3_-N_3_ (**6b**)

The irradiation of **6a** in methanol or DCM with a 350 nm fluorescent lamp or LED results in the clean formation of the 18-membered macrocycle **8** (Scheme 3). The duration of irradiation required to achieve the complete consumption of the starting material ranges from 15 s to 5 min depending on the initial concentration of **6a**.^19^ The macrocycle **8** has been isolated in 95% yield in preparative photolysis. Azide groups are stable under 350 nm irradiation, apparently, due to a very low extinction coefficient at this wavelength (ε < 0.1).^19^

**Scheme 3:**
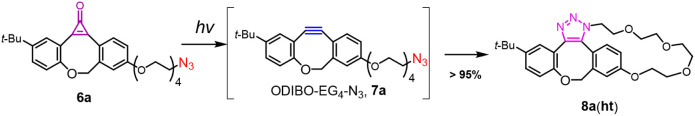
Photolysis of Photo-ODIBO-EG_4_-N_3_ (**6a**)

The SPAAC cyclization of the intermediate ODIBO-EG_4_-N_3_ (**7a**) can produce two isomeric cycles due to head-to-head and head-to-tail addition of azide to the ODIBO moiety (Figure 1). DFT analysis suggests that the regioisomer **8a**(**ht**) is formed faster and is more stable than **8a**(**hh**) (*vide infra*). No evidence of a second isomer was observed in the NMR spectra and in the HPLC analysis of the photolysate. Two-NMR through-bond (HMBC) and through-space (NOESY) correlation spectra did not allow us to assign the regiochemistry of the cyclic product. On the basis of DFT calculations, we have assigned macrocycle **8** the structure **8a**(**ht**).

**Figure 1:**
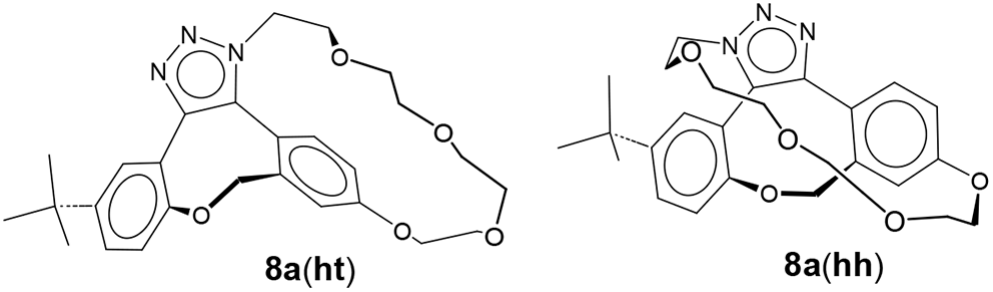
DFT B3LYP/6-311G+d,p optimized geometries of two potential regioisomers of the macrocycle **8a**.

The mass spectra of the crude photolysate of 10 μM to 0.5 mM solutions of photo-ODIBO-EG_4_-N_3_ (**6a**) show no detectable amounts of dimers or oligomers. Interestingly, MS analysis detected only macrocycle **8a** (Calcd for C_27_H_34_N_3_O_5_^+^ 480.2493), even when **6a** was irradiated in the neat form, deposited as a thin layer on a glass slide (Figure S4).^19^ A small peak (∼12%) with a mass (959.4868) corresponding to the protonated dimer (959.4919 for 2M + H^+^) was also observed; however, collision-induced MS/MS spectra of the latter ion produced only one fragment peak corresponding to **8a**, suggesting the formation of a noncovalent dimer in the ionization chamber of the MS instrument, rather than a covalent dimer (Figure S5).^19^ No peaks corresponding to the formation of a trimer or heavier oligomers have been detected. To distinguish between products containing the cyclooctyne moiety (e.g., **7a**) from closed isomers (e.g., **8a**) in mass spectra, a large excess of sodium azide was added to the photolyzates after the completion of the reaction, as judged by UV spectra (Figure 2). Sodium azide rapidly adds to dibenzocyclooctynes (such as **2a**,**b**) to produce the corresponding 2*H*-1,2,3-triazoles (Scheme 4).^20^ No detectable amounts of azide ion–cyclooctyne cycloaddition products (M + 43) were observed in photolyzates of all macrocycle precursors (**6a**,**b**, **9a,b, 12**).

**Scheme 4:**
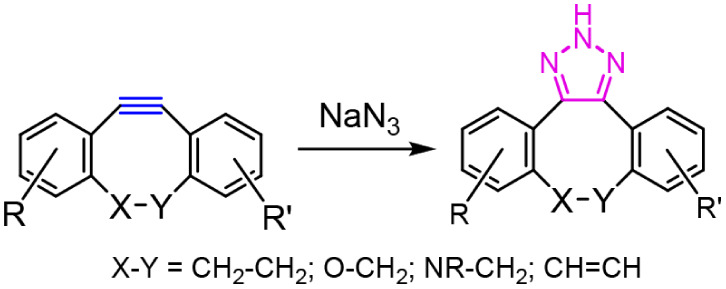
Formation of 2*H*-1,2,3-Traizoles in Reaction of Dibenzocyclooctynes with Azide Ion

**Figure 2:**
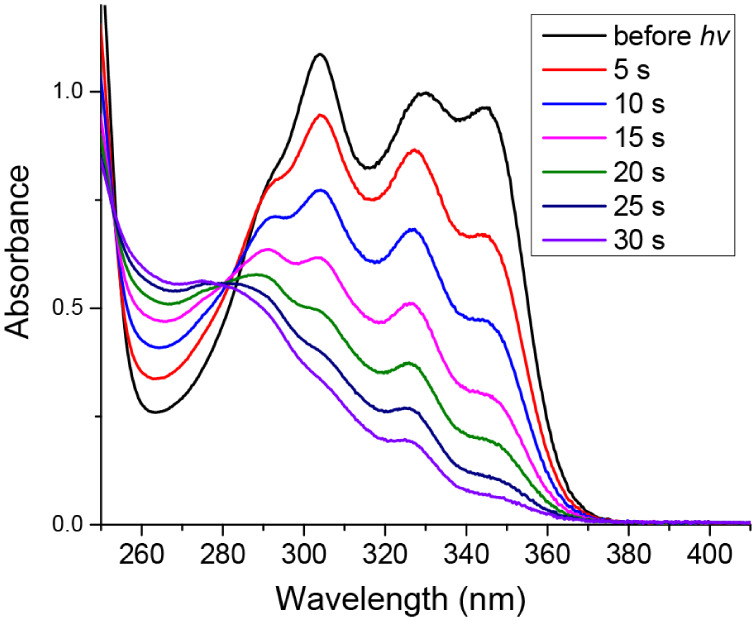
Changes in UV spectra during 350 nm irradiation of 50 μM methanol solution of photo-ODIBO-EG_4_-N_3_ (**6a**).

The photodecarbonylation of the cyclopropenone moiety in photo-ODIBO-EG_4_-N_3_ (**6a**) should initially produce ODIBO-EG_4_-N_3_ (**7a**).^17^ However, UV spectra recorded during irradiation of the former show only consumption of the starting material and the formation of the triazole product. No characteristic ODIBO band^17^ at 324 nm was observed (Figure 2).

The actual rate of ODIBO-EG_4_-N_3_ (**7a**) cyclization has been determined using an LED flash photolysis experiment.^19^ Briefly, solutions of the starting material in MeOH or DCM were irradiated for 15 s with a 1W 340 nm LED to achieve quantitative conversion of the starting material. The changes in absorbance at 324 nm (ODIBO band)^17^ were recorded immediately after the termination of exposure (Figure 3). Since the SPAAC cyclization is a unimolecular process, the observed rate constant and the half-life of the intermediate **7a** should be independent of the initial concentration. In fact, the rates of reaction in DCM at 25 °C were the same at 10, 50, and 200 μM of **6a**, *k*_obs_ = 0.0066 ± 0.0001 s^–1^, and 50% conversion of ODIBO-EG_4_-N_3_ (**7a**) was achieved in 104 ± 1 s at all three concentrations (Figure 3). This observation indicates that the absolute majority of the product(s) is formed in a unimolecular process, supporting the results of the product analysis. It is also interesting to note that the rate of SPAAC cyclization strongly depends on the polarity of the solvents. Thus, the rate increases from DCM (*k*_obs_ = 0.0066 ± 0.0001 s^–1^) to MeOH (*k*_obs_ = 0.0153 ± 0.0007 s^–1^) to aqueous solution (*k*_obs_ = 2.8 ± 0.1 s^–1^). A similar effect of solvent polarity on the rate of SPAAC reactions has been reported previously.^17,21,22^

**Figure 3:**
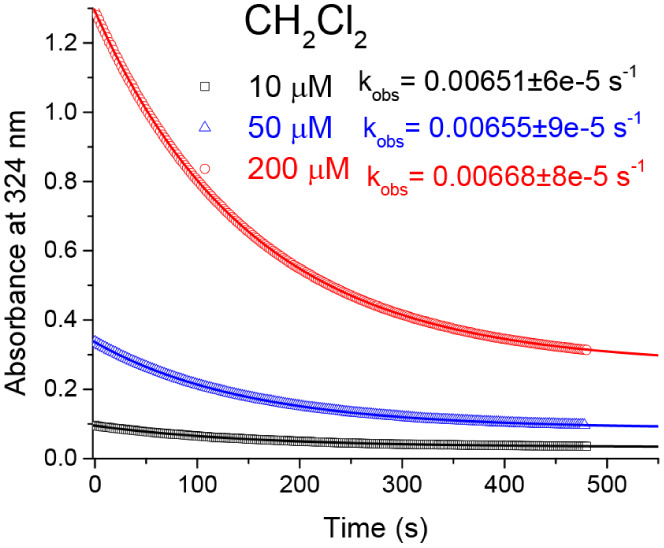
Decay of absorption of ODIBO-EG_4_-N_3_ (**7a**) at 324 nm in the LED flash photolysate of **6a** in DCM at various concentrations.

In an attempt to evaluate the EM of ODIBO-EG_4_-N_3_ (**7a**), the product of its SPAAC reactions was analyzed at 0.12 M concentration. The short lifetime of **7a** (τ_1/2_ ∼ 1.7 min), however, prevents generating concentrations above 0.5 mM of **7a** in homogeneous solutions, as at concentrations of the starting cyclopropenone **6a** above 1 mM, the photodecarbonylation, rather than cyclization, becomes the rate-limiting step of the process. Thus, at 60 mM in DCM, it took 8 min of irradiation at 350 nm for the complete consumption of the starting material **6a**. Apparently, the highest concentration of dibenzocyclooctyne **7a** achieved in this experiment was well below 60 mM. To increase the effective concentration of alkyne **7a**, while keeping the overall amount of the starting cyclopropenone low enough for rapid photoconversion, we have developed an emulsion-based photolysis technique.^19^ Briefly, 50 μL of 0.12 M solution of **6a** in dichloromethane was injected into 10 mL of rapidly stirred brine, creating an emulsion where the concentration of cyclopropenone **6a** within the droplets of DCM was 0.12 M, but the total amount of **6a** in the cuvette was equal to 0.6 mM solution. Thirty seconds of irradiation resulted in the complete consumption of **6a**, creating a concentration of ODIBO-EG_4_-N_3_ (**7a**) close to 0.12 M. After 30 min of incubation in the dark and treatment with an excess of sodium azide, the ESI/MS analysis showed only the prominent peak at *m*/*z* = 480.3, corresponding to macrocycle **8a**. No traces of **7a**, covalent dimer, or their adducts with the azide ion were detected.

350 nm photolysis of a 1 mM DCM solution of photo-ODIBO-EG_3_-N_3_ (**6b**) produced a mixture of products: 15-membered cycle **8b** and three isomeric 30-membered cyclic dimers of **7b** (Scheme 5). It is interesting to note that no detectable amounts of acyclic dimers or higher oligomers were observed (Figure S6).^19^ This observation suggests that EM for the SPAAC reaction of X-DIBO-linker-N_3_ is reduced when the linker length is shorter than 11 atoms; however, the dimeric byproducts undergo quantitative cyclization without the formation of longer oligomers.

**Scheme 5:**
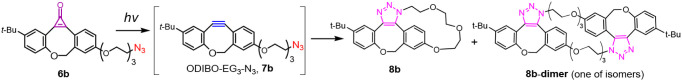
Photolysis of Photo-ODIBO-EG_3_-N_3_ (**6b**)

### Light-Induced Cyclization of Photo-DIBO-EG_4_-N_3_ (**9**)

The irradiation of 10–30 μM DCM solutions of photo-DIBO-EG_4_-N_3_ (**9a,b**) for 2 min at 350 nm results in the complete consumption of the starting material, as evidenced by the bleaching of the characteristic 345 and 329 nm bands of the photo-DIBO chromophore. The resulting UV spectrum corresponds to DIBO-EG_4_-N_3_ (**10a,b**) with characteristic bands at 321 and 304 nm (Figure S1).^19^ DIBO-EG_4_-N_3_ (**10a,b**) then undergoes an SPAAC reaction to yield the 18-membered macrocycle **11a,b** (Scheme 6). The MALDI-TOF MS spectra of the photolyzates of **9a** contained only peaks with *m*/*z* = 494.5, which corresponds to macrocycle **11a** (Calcd 494.26 for C_26_H_36_N_3_O_5_^+^, Figure S7a). The MS/MS spectrum of a presumable dimer peak at 987.8 (Calcd 987.52 for 2M + H^+^) confirms this peak to be a noncovalent dimer of **11a**. Similar results were obtained in the 350 nm photolysis of 2-hydroxyethyl-substituted photo-ODIBO-EG_4_-N_3_ (**9b**, Figure S7b). In both cases, no detectable peaks of oligomers were observed, indicating the quantitative formation of the macrocycles **11a**,**b** in the photo-SPAAC cyclization of photo-DIBO-EG_4_-N_3_ (**9a**,**b**).

**Scheme 6:**
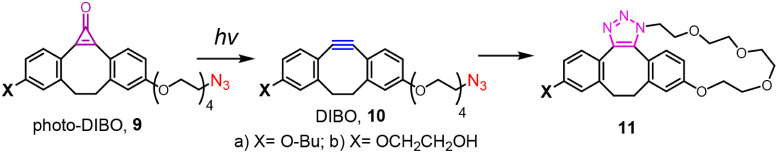
Photolysis of Photo-DIBO-EG_4_-N_3_ (**9a,b**)

The kinetics of the SPAAC cyclization of **10b** was monitored by the decay of absorbance at 321 nm at 25 °C in DCM, which followed the first-order rate law well (Figure S2).^19^ The observed rate, *k*_obs_ = (3.70 ± 0.1) × 10^–4^ s^–1^, was the same at 10 and 20 μM concentrations of the starting photo-DIBO-EG_4_-N_3_ (**9b**) before irradiation, supporting the intramolecular nature of the reaction. The cyclization of **10b** was ∼2.5 times faster in methanol, *k*_obs_ = (1.08 ± 0.01) × 10^–3^ s^–1^. Approximately a 10-fold rate reduction of the SPAAC cyclization reaction of **10b** versus **7** corresponds to the lower reactivity of DIBO cyclooctynes.^17^

### Light-Induced Cyclization of Photo-ODIBO-O­(CH_2_)_11_-N_3_ (**12**)

To test if the unusually efficient macrocyclization of bifunctional molecules **7** and **10** is facilitated by the tetraethylene glycol linker between azido and cyclooctyne moieties, the reactivity of photogenerated ODIBO-O­(CH_2_)_11_-N_3_ (**13**, Scheme 7) with a same length *n*-alkyl linker was studied. Photo-ODIBO-O­(CH_2_)_11_-N_3_ (**12**) was prepared following the same general procedure as for **6a**.^19^ DCM or methanol solutions of **12** were irradiated at 350 nm until the complete consumption of the starting material, which was evident by the bleaching of the cyclopropenone band at 345 nm (Figure 5). The resulting spectrum contains bands at 311 and 326 nm, characteristic of the ODIBO chromophore (Figure 5), which is indicative of the formation of ODIBO-O­(CH_2_)_11_-N_3_ (**13**, Scheme 7). The photolysate was incubated for 24 h in the dark at ambient temperature and quenched with an excess of sodium azide. The ESI-MS of the resulting mixture shows only peaks belonging to the macrocycle **14** (Scheme 7). No dimers or heavier oligomers, nor products of azide ion addition, were detected. The exclusive formation of the macrocycle **14** was observed at 10, 50, and 200 μM, as well as in the neat form. Preparative photolysis of photo-ODIBO-O­(CH_2_)_11_-N_3_ (**12**) allowed us to isolate two isomers of the macrocycle: **14a** (74%) and **14b** (21%, Scheme 7).^19^

**Scheme 7:**
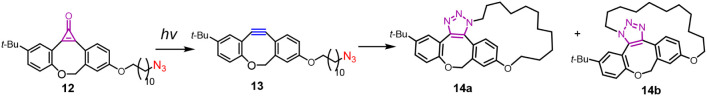
Photolysis of Photo-ODIBO-O­(CH_2_)_11_-N_3_ (**12**)

As in the case of **8a**, HMBC and NOESY spectra of **14a and 14b** were not helpful. The assignment of the structure **14a** to the major isomer produced in the cyclization of ODIBO-O­(CH_2_)_11_-N_3_ (**13**) is based on the results of DFT calculations, which predict the faster formation of regioisomer **14a** (*vide infra*). It is interesting to note that several signals in NMR spectra of the minor isomer **14b** are doubled at room temperature Upon heating to 75 °C, some of these signals coalesce to produce broad peaks (Figure S8).^19^ These observations indicate the presence of relatively slow conformational equilibrium in solutions of **14b**. According to DFT calculations (*vide infra*), there are two relatively stable conformers of **14b,** which are close in energy (Figure 4). The energy gap between conformations of **14a** is much higher, according to DFT calculations. In fact, the NMR spectra of **14a** show no evidence of conformational equilibria under ambient conditions Figure 5.

**Figure 4:**
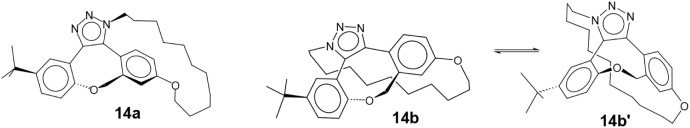
DFT B3LYP/6-311G+d,p optimized geometries of two regioisomers of the macrocycle **14**. Two conformations of a minor regioisomer **14b** predicted by DFT calculations.

**Figure 5:**
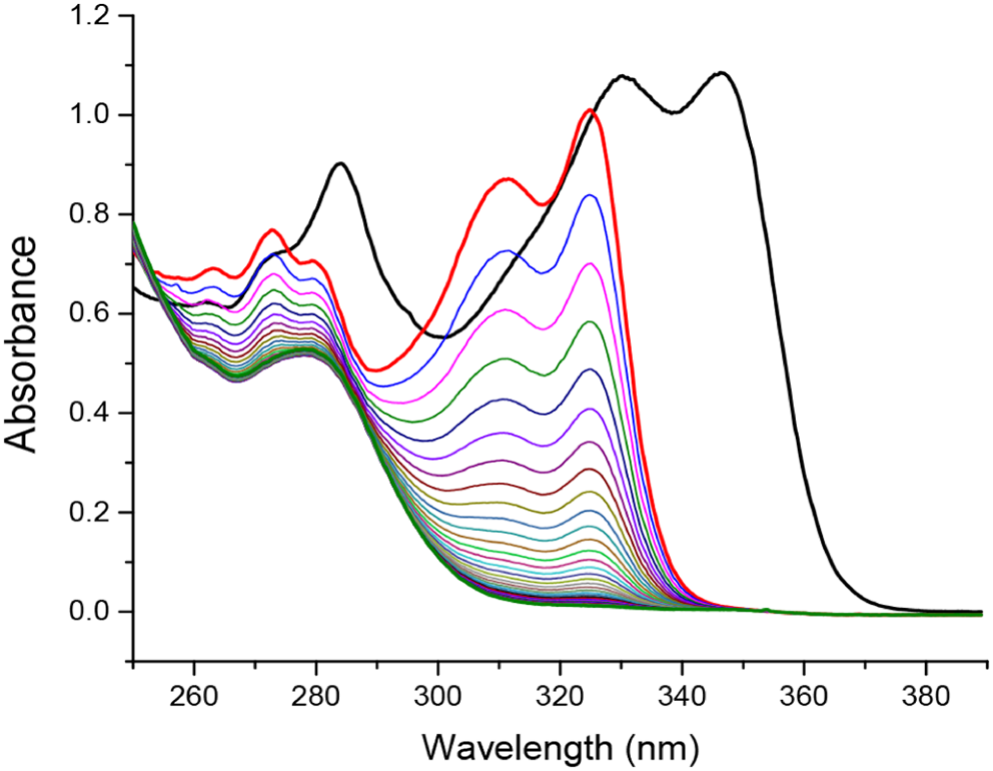
Changes in UV spectra during 340 nm irradiation of 50 μM methanol solution of photo-ODIBO-O­(CH_2_)_11_-N_3_ (**12**).

The consumption of **13** followed the first-order rate law well (Figure 6). The rate of SPAAC cyclization of **13**, as well as the half-lifetime of **13**, was independent of the concentration of the starting material (Figure 6), supporting the unimolecular reaction of **13**. As in previous examples (**7** and **10**), the reaction was faster in methanol (*k*_obs_ = (2.14 ± 0.05) × 10^–4^ s^–1^) than in DCM (*k*_obs_ = (1.012 ± 0.005) × 10^–4^ s^–1^).^19^ The 60-fold reduction of the rate of the SPAAC cyclization of **13** versus **7** probably reflects the increased Δ*H* ^‡^ of the cyclization due to the reduced flexibility of the *n*-alkyl linker versus the tetraethylene glycol linker, as there are no gauche or eclipsed interactions around C–O bonds in **7**. In addition, the inductive and field effects of oxygen atoms make EG_4_-N_3_ more reactive than alkyl azide in the SPAAC reaction. The difference in the cyclization rates agrees very well with the results of DFT calculations (*vide infra*).

**Figure 6:**
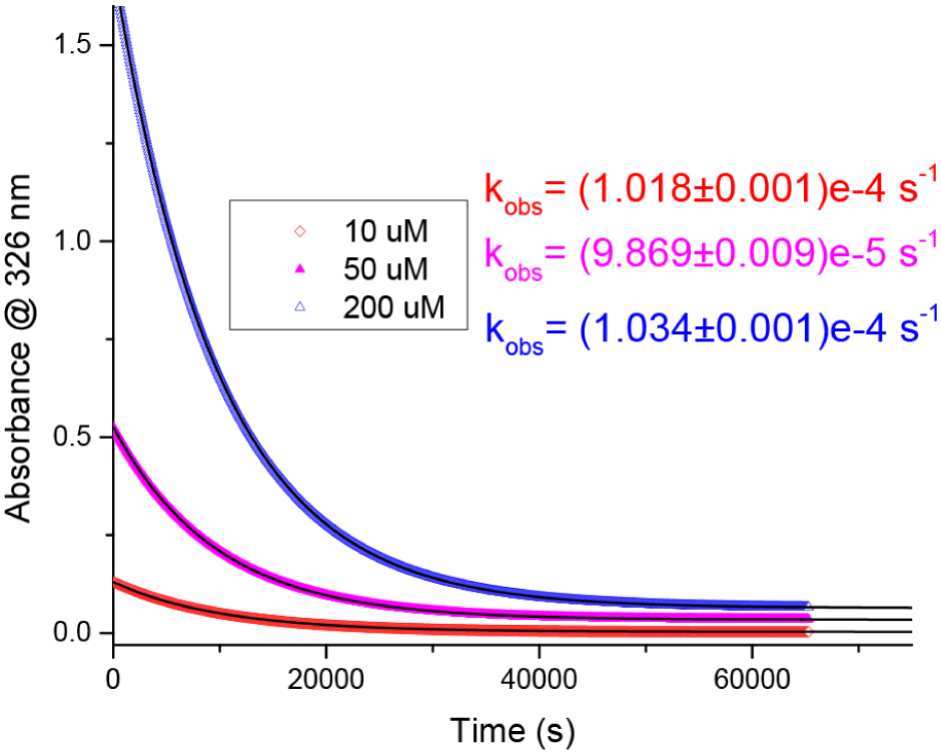
Decay of absorption at 326 nm of ODIBO-O­(CH_2_)_11_-N_3_ (**13**) in DCM in the photolysate of **12** at various concentrations of the starting material.

In fact, the reaction of ODIBO-OH (**15**) with EG_4_-N_3_ (**16a,** *k* = 0.51 ± 0.02 M^–1^s^–1^ in MeOH and 0.25 ± 0.01 M^–1^s^–1^ in DCM) was two times faster than with 11-azidoundecan-1-ol (**16b**, *k* = 0.24 ± 0.01 M^–1^s^–1^ in MeOH; 0.124 ± 0.008 M^–1^s^–1^ in DCM) Scheme 8). It should also be noted that **15** is somewhat less reactive than ODIBO-OMe.^17^

**Scheme 8:**
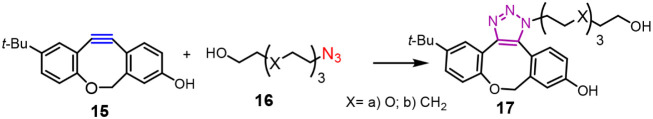
SPAAC Addition of HO-EG_4_-N_3_ (**16a**) and 11-Azidoundecan-1-ol (**16b**) to ODIBO (**15**)

### Determination of the Activation Parameters for the Cyclization and Dimerization Reactions ODIBO-EG_4_-N_3_(**7**) and ODIBO-O­(CH_2_)_11_-N_3_ (**13**)

In order to understand the unusually high effective molarity of SPAAC reactions of bifunctional ODIBO-EG_4_-N_3_ (**7a**) and ODIBO-O­(CH_2_)_11_-N_3_ (**13**), the rates of cyclization reactions were measured at different temperatures in the range of 15 to 65 °C in ethanol. Temperature-equilibrated solutions of **6a** and **12** (40 μL in ethanol) were irradiated by the 350 nm lamps until the complete consumption of the starting cyclopropenones was achieved (∼15 s), and the consumption of the corresponding cyclooctynes **7a** and **13** was followed by UV spectroscopy (*vide supra*). MS analysis of the photolyzates of **7a** and **13** incubated at 65 °C indicates that macrocycles **8a** and **14** remain the principal product as no dimers were detected. The temperature dependence of the observed rate constants allowed us to determine activation parameters (i.e., Δ*H* ^‡^ and Δ*S* ^‡^) of the SPAAC cyclization of **7a** and **13** (Figure 7). As should be expected for the macrocyclization, reactions of both compounds show highly negative entropy of activation. Interestingly, Δ*S* ^‡^ = 37 cal mol^–1^K^–1^ is the same for both compounds. The enthalpy of activation for the cyclization of **7a** is ∼2.5 kcal mol^–1^ lower than for **13**, which manifests itself in the faster reaction of the former. This difference agrees well with the results of DFT calculations (*vide infra*).

**Figure 7:**
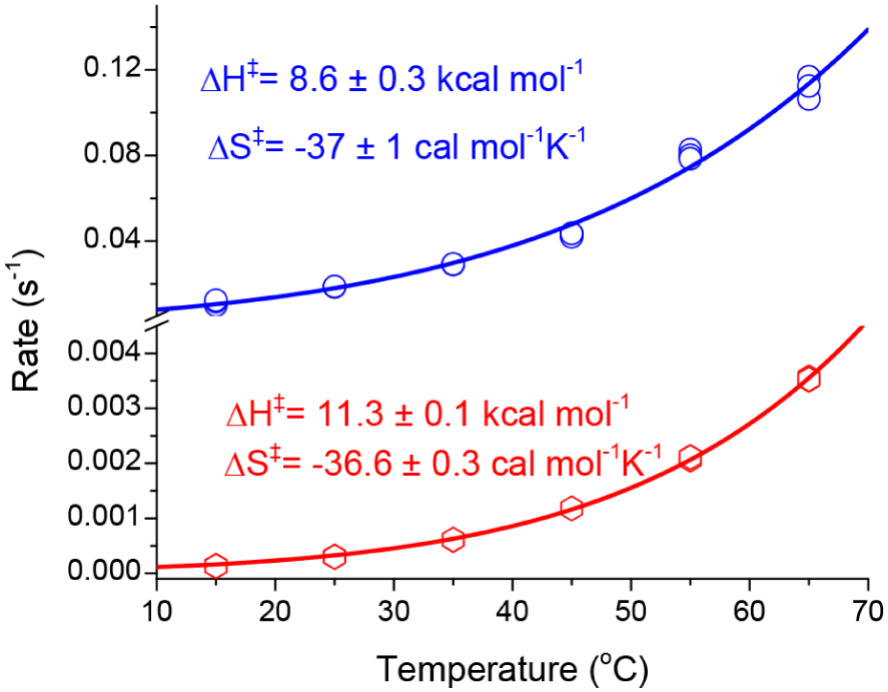
Temperature dependence of the cyclization rate of EtOH solutions of ODIBO-EG_4_-N_3_ (**7a**, blue circles) and ODIBO-O­(CH_2_)_11_-N_3_ (**13**, red hexagons).

To emulate the dimerization reaction of **7a**, we have studied the temperature dependence of the rate of addition of photo-ODIBO-EG_4_-N_3_ (**6a**) to ODIBO-EG_4_-TBS (**18**), as the actual dimerization reaction of **7a** cannot be directly observed (Scheme 9).

**Scheme 9:**
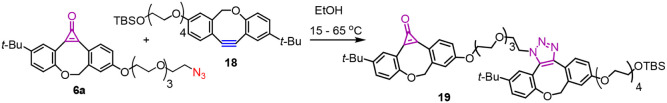
SPAAC Additions of Photo-ODIBO-EG_4_-N_3_ (**6a**) to ODIBO-EG_4_-TBS (**18**)

The second-order rate constant of this SPAAC reaction can be evaluated from 2.25 ± 0.03 M^–1^s^–1^ at 25 °C to 13 ± 1 M^–1^s^–1^ at 65 °C (Table S3).^19^ These rates are in agreement with previously reported rates of the SPAAC reaction of the parent ODIBO.^17^ The intra- versus intermolecular rate difference indicates that Δ*G* ^‡^ of activation for the dimerization reaction is ∼3 kcal lower than that of cyclization at 25 °C. This observation qualitatively agrees with the results of DFT calculations (*vide infra*). The value of EM (∼0.01 M at 25 °C in DCM) estimated from the rates of cyclization of ODIBO-EG_4_-N_3_ (**7a**) and the reaction presented in Scheme 8, however, disagrees with the exclusive formation of macrocycle **8** at 0.12 M concentration and in the neat form (*vide supra*).

From the temperature dependence of the observed rate of the SPAAC addition of azide **6a** to cyclooctyne **18,** we can evaluate Δ*H* ^‡^ = 8.3 ± 0.4 kcal mol^–1^ and Δ*S* ^‡^ = −29 ± 1 cal mol^–1^ K^–1^ for the process (Table S3, Figure 8). Interestingly, the enthalpies of activation for the inter- and intramolecular SPAAC reactions of **7a** are the same, suggesting that there is no detectable buildup of ring strain in the transition state leading to the macrocycle **8a**. Expectedly, Δ*S* ^‡^ for the intermolecular SPAAC reaction of **7a** is 8 cal mol^–1^ K^–1^ less negative than for the intramolecular counterpart. However, its value still suggests a rather tight transition state for the dimerization reaction.

**Figure 8:**
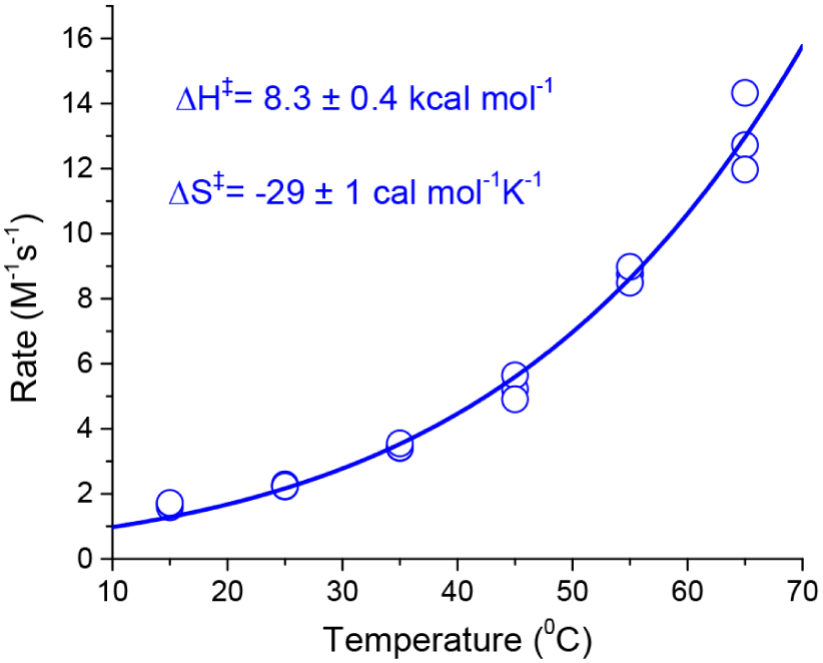
Temperature dependence of the SPAAC addition rate in EtOH of photo-ODIBO-EG_4_-N_3_ (**6a**) to ODIBO-EG_4_-Ts (**18**).

### Photo-SPAAC Synthesis of 51-Membered Macrocycle

The photo-SPAAC cyclization proved to be an efficient strategy for the synthesis of medium-sized macrocycles **8a**, **11a,b**, and **14**. To explore the utility of this reaction for the preparation of larger macrocycles, we have explored the photo-SPAAC cyclization of the bifunctional compound **20** (Scheme 10). The latter possesses a different cyclopropenone-caged dibenzocyclooctyne, i.e., photo-DIBO, at one terminus and an azide moiety at the other. 350 nm irradiation of **20** results in the decarbonylation of the cyclopropenone moiety and the formation of the dibenzocyclooctyne **21**, as evident from the bleaching of the photo-DIBO band at 346 nm and the formation of the characteristic 302 and 319 nm bands of the DIBO chromophore (Figure 9).^16^ After 24 h incubation of the photolysate in the dark at ambient temperature, a large excess of sodium azide was added, and the resulting mixture was analyzed by MALDI-TOF mass spectrometry. As in the case of **6a**, **9**, and **12**, the only prominent peak in the mass spectrum was observed at 1109.7 (ESI MS), which corresponded to the macrocycle **22** (Scheme 10). The clean formation of **22** was observed in DCM, MeOH, or DMSO upon photolysis of **20** at concentrations up to 60 mM. No heavier ions were detected up to 3500 kDa. Preparative photolysis of **20** produced macrocycle **22** in a quantitative yield.^19^

**Figure 9:**
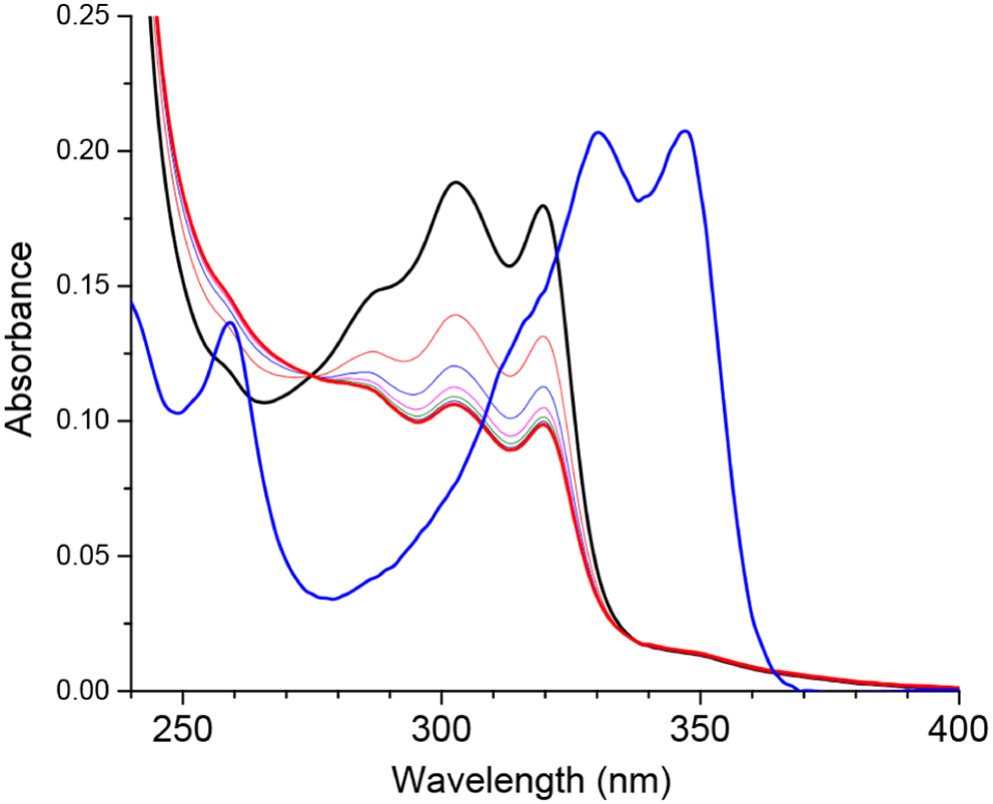
UV spectra of a 10 μM methanol solution of cyclo-propenone **20** before (blue line) and immediately after 350 nm photolysis (black line). The spectra of the consumption of dibenzocyclooctyne **21** (black line) were recorded every 8 min.

**Scheme 10:**
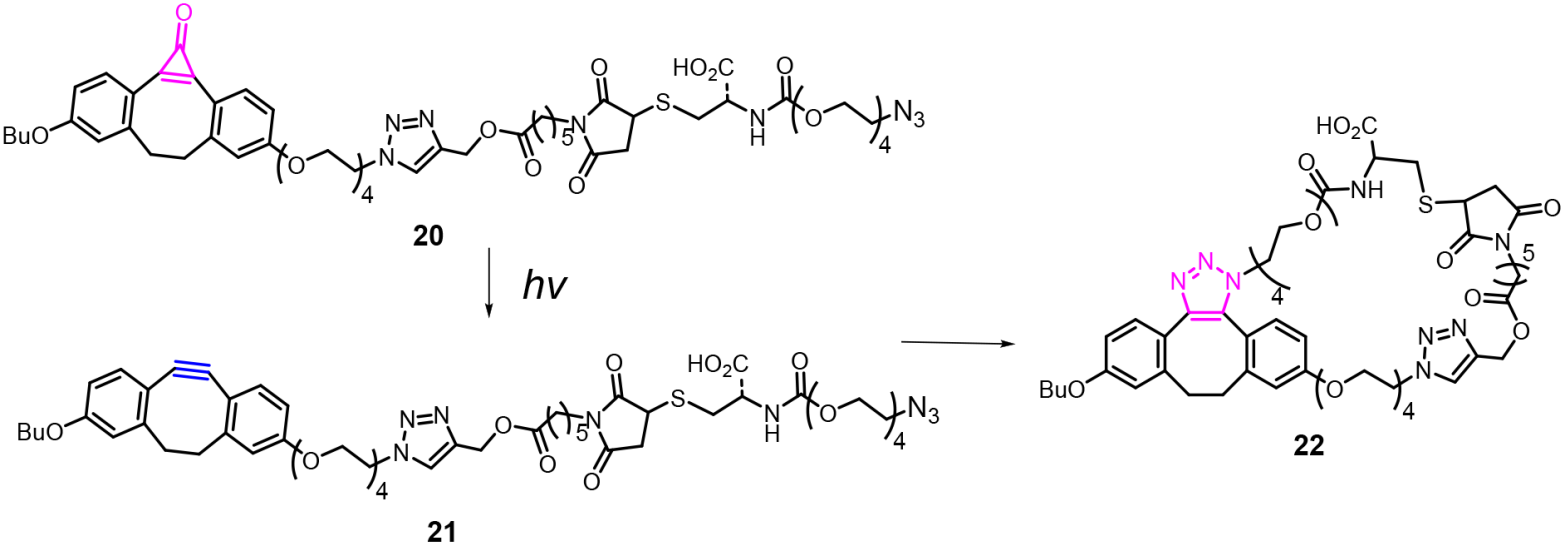
Photolysis of Photo-DIBO-linker-N_3_ (**20**)

As in the case of photo-ODIBO-EG_4_-N_3_ (**6a**), the photodecarbonylation of **20** becomes the rate-limiting step in homogeneous solutions at concentrations above 60 mM, limiting the maximum concentration of **21**. To explore the outcome of SPAAC cyclization at higher concentrations of the latter, the emulsion-based photolysis technique has been employed (100 μL of 120 mM solution of **20** in 2 mL of DCM).^19^ The MS of the DCM layer of the photolysate contained only two peaks: the major one at 1109.7 (corresponding to M + H^+^, C_53_H_73_N_8_O_16_S^+^) and the minor one (∼10%) at 2218.4 (2M + H^+^, C_106_H_145_N_16_O_32_S_2_^+^, Figure S9).^19^ The latter ion was found to be a noncovalent dimer of **22** by MS/MS analysis. These MS data have confirmed the complete consumption of the starting material **20** and the clean formation of the macrocycle **22**. Finally, a glass slide was spin-coated with cyclopropenone **20** from a DCM solution, creating a thin layer of the neat substrate. After 1 min of 350 nm irradiation, the photolysate was washed from the glass slide with MeOH, containing 1 M of sodium azide to quench unreacted alkynes. The MS analysis indicated the quantitative formation of the macrocycle **22** (Figure S10).^19^

The rate of the SPAAC cyclization of DIBO-azide **21** was measured using UV spectroscopy by following the decay of the 319 nm band of the DIBO chromophore (Figure 9). The resulting data followed the first-order rate law. The rates and the half-life of **21** were independent of the substrate concentration in the range of 10 μM to 100 μM, *k*_obs_ = (1.03 ± 0.01) × 10^–3^ s^–1^ (Figure 10), confirming the unimolecular nature of the reaction.

**Figure 10:**
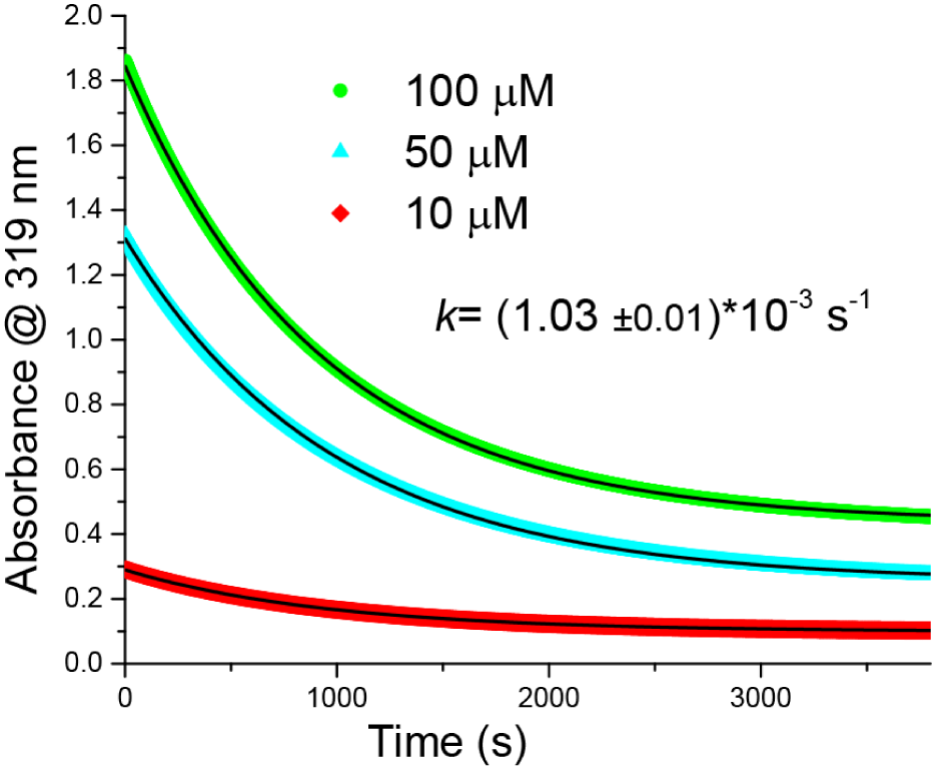
Decay of absorption at 319 nm of dibenzocyclooctyne **21** in DCM in the photolysate of **20** at various concentrations of the starting material.

As in previous cases, the rate of cyclization was progressively faster in more polar solvents, DCM (*k*_obs_ = (1.03 ± 0.01) × 10^–3^ s^–1^), methanol (*k*_obs_ = (2.2 ± 0.3) × 10^–3^ s^–1^), and water:MeOH (9:1) (pH = 7.4, *k*_obs_ = (1.77 ± 0.02) × 10^–2^ s^–1^).

### Synthesis of Macrocycles by Double-SPAAC Cross-Coupling

An alternative strategy for the preparation of macrocycles is based on a cross-coupling of two bifunctional components, each carrying two identical moieties at opposite termini.^10^ In fact, the formation of macrocycles in the double-SPAAC reaction between bis-cyclooctyne, e.g., DIBOD (**24**, for DIBenzocycloOctaDiyne, a.k.a. Sondheimer diyne), and bis-PEG-azides has been reported before.^15^ Major complications in the use of DIBOD (**24**) for macrocycle synthesis are the low stability of this compound and the huge difference between the first and second SPAAC reactions of diyne **24**. While the first addition of azide proceeds at a rate similar to DIBO (*k* ∼ 0.07 M^–1^s^–1^),^23,24^ the second reaction is more than 500 times faster (*k* ∼ 34 M^–1^s^–1^).^25^ To address these issues, we have employed photo-DIBOD (**23**), a bis-cyclopropenone caged photoprecursor to **24**, which quantitatively produces **24** upon exposure to UVA light.^24^ Photo-DIBOD (**23**) possesses unique thermal stability and allows for the sequential activation of dibenzocyclooctyne moieties.^25^ The photo-DIBOD (**23**)–bis-azide two-component macrocycle preparation strategy provides access to several macrocyclic structures from the same set of precursors. For example, photolysis of **23** in the presence of 1 eq. of bis-azide-TEG (**25**) yields 80% of the 17-membered macrocycle **26**, along with 15% of a cyclic dimer **27** and trace amounts of a cyclic trimer (Scheme 11, Figure S11).^19^ The NMR spectra of the isolated macrocycle **26** show the presence of two regioisomers in a ca. ratio of 2:1.^19^ We were not able to separate the isomers and assigned the major isomer a less constrained structure, **26a**. We have also assigned a cyclic structure to the dimer **27** since the photolyzates were quenched by the addition of sodium azide, which converts all unreacted dibenzocyclooctyne moieties into 2*H*-1,2,3-triazoles (Scheme 4).

**Scheme 11:**
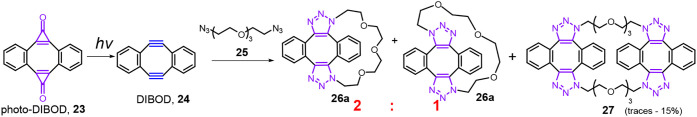
Photochemical Generation of DIBOD (**24**) in the Presence of Bis-TEG Azide (**25**)

Slow addition of 1 eq. of **25** (over 2 h) to the solution of photogenerated DIBOD **24** produces an almost quantitative yield of **26a**,**b** with only trace amounts of the dimer, according to MS data (Figure S12).^19^ Slow (over 2 h) photolysis of **23** in the presence of 1 eq. of **25** produces similar results.

Controlled irradiation of photo-DIBOD (**23**) allows for the preparation of MC-DIBOD **28** (Mono Cyclopropenone caged DIBOD) by monodecarbonylation of the starting material (Scheme 12).^25^ A reaction of **28** with 1 eq. of bis-azido-EG_4_ **25** yields bifunctional MC-DIBOT-EG_4_-N_3_ (**29**, DIBOT for mono-Triazole of DIBOD, Scheme 12).

**Scheme 12:**
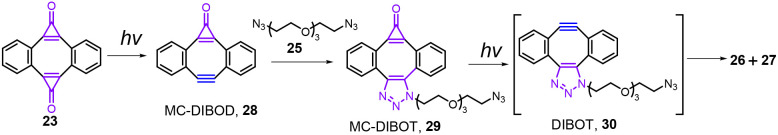
Preparation and Light-Induced Macrocyclization of MC-DIBOT-EG_4_-N_3_ (**29**)

Photodecarbonylation of a 30 mM DCM solution of MC-DIBOT-EG_4_-N_3_ (**29**) under 350 nm irradiation yields the intermediate DIBOT-EG_4_-azide (**30**), which undergoes rapid SPAAC cyclization to produce macrocycles **26** and ∼5% of the dimer **27** (Scheme 12).

When MC-DIBOD (**28**) is allowed to react with 0.5 eq. of bis-azido-EG_4_ **25**, bis-MC-DIBOT adduct **31** is produced. Irradiation of a 30 μM DCM solution of bis-cyclopropenone **31** in the presence of an equimolar amount of **25** produces the 34-membered macrocycle **27** (Scheme 13). The MALDI-TOF mass spectrum shows no other products in the photolysate (Figure S12).^19^ The NMR spectra of the isolated **27** indicate that two regioisomeric cycles, designated as “head”-to-head” (**27a**) and “head-to-tail” (**27b**), are formed in a 1:1 ratio.

**Scheme 13:**
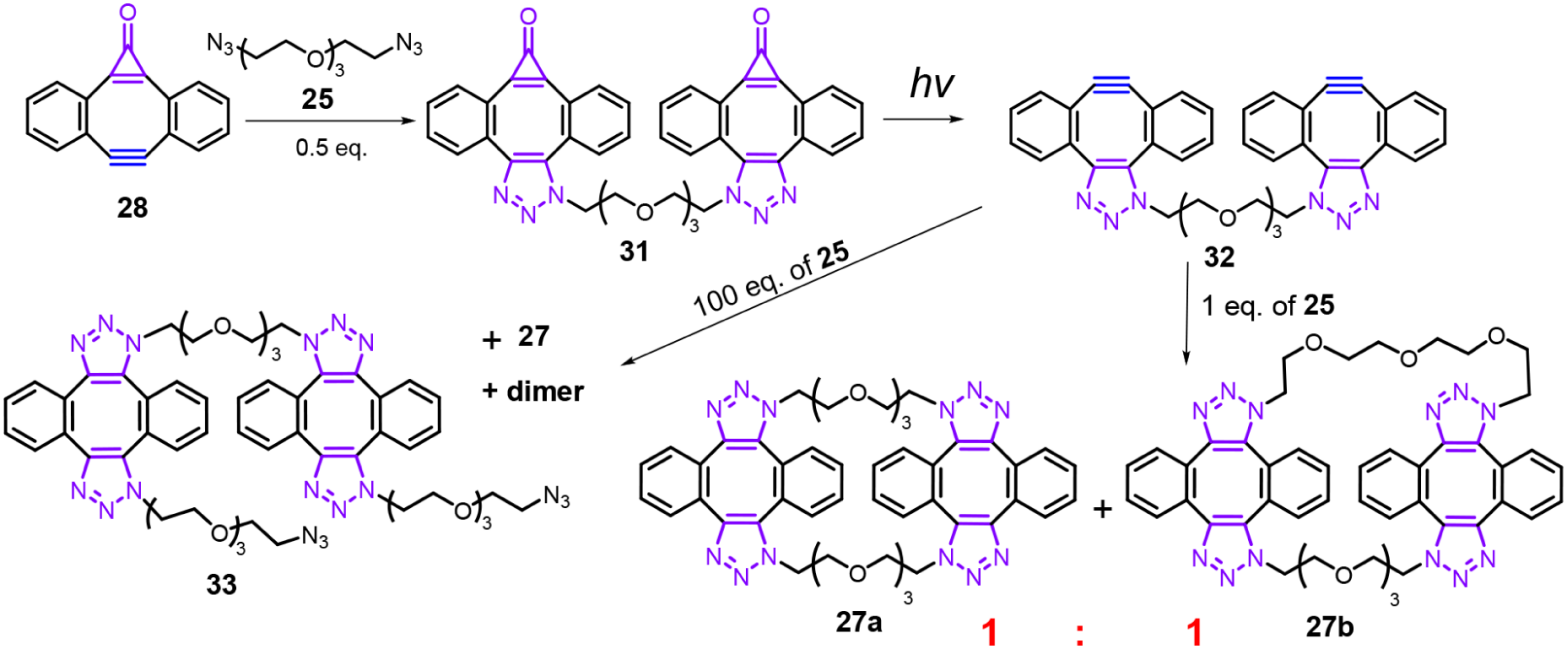
Preparation and Photo-SPAAC Reaction MC-DIBOT-EG_4_-MC-DIBOT (**31**)

This reaction, apparently, proceeds via the photogenerated intermediate bis-DIBOT (**32**), which undergoes sequential SPAAC reactions with bis-azide **25** (Scheme 13). In fact, the consumption of **32**, followed by the decay of the 360 nm band of the DIBOT chromophore, showed biphasic behavior, indicating sequential reactions (Figure S3).^19^ The rate of the first reaction, the SPAAC addition of **25** to **32**, is estimated at *k*_AD_ *∼* 23 M^–1^s^–1^, and the rate of the SPAAC cyclization step is *k*_cycl_ ∼ 0.002 s^–1^.

The ESI HRMS of the photolysate of **31** in the presence of a 100-fold excess of bis-azido-EG_4_ **25** shows that bis-adduct **33** (1133.5172, calcd. for C_56_H_65_N_18_O_9_^+^: 1133.5176) is the major product, along with ∼15% of macrocycle **27** (889.3890, calcd. for C_48_H_49_N_12_O_6_^+^: 889.3893) and ∼20% of the dimer, i.e., 2 × **32** + 3 × **25** (2022.9023, calcd. for C_104_H_113_N_30_O_15_^+^: 2022.9030, Figure S13).^19^ The ratio of the products agrees well with the above rate estimate, assuming that the addition of the second molecule of **25** proceeds at a rate similar to that of the first addition.

### DFT Analysis of SPAAC Cyclization of ODIBO-EG_4_-N_3_ (**7a**) and ODIBO-O­(CH_2_)_11_-N_3_ (**13**)

The cyclization pathways of two bifunctional dibenzocyclooctyne-azide compounds **7a** and **13** were analyzed computationally at the density functional theory (DFT) level, as the DFT calculations were extensively employed for the exploration of the mechanism, reactivity, and driving forces of the SPAAC reactions.^26^ Geometries of the starting ODIBO-EG_4_-N_3_ (**7a**) and ODIBO-O­(CH_2_)_11_-N_3_ (**13**), SPAAC transition states, as well as the final products **8a** and **14a,b**, were optimized using the B3LYP functional and 6-311+G­(d,p) basis set. Electronic energies and vibrational frequencies were computed at the same level.^19^

The conformational space of starting cyclooctynes, ODIBO-EG_4_-N_3_ (**7a**) and ODIBO-O­(CH_2_)_11_-N_3_ (**13**), is, obviously, very complex. We have briefly explored it by conducting B3LYP/6-31+G­(d) restricted geometry optimization with the distance between the central nitrogen atom of the azide group and the carbon of the triple bond fixed at 5, 10, and 15 Å (in the fully extended, all anticonformation, this distance is ∼21 Å). The resulting structures were then reoptimized with no restrictions. The geometry of conformations obtained in such a manner was reoptimized at the B3LYP/6-311+G­(d,p) level. Expectedly, the most stable conformations of **7a** and **13** were found to have fully extended linkers (Figures 11 and 12). Therefore, the extended conformations of both **7a** and **13** have been selected as the reference points for the energy calculations.

**Figure 11:**
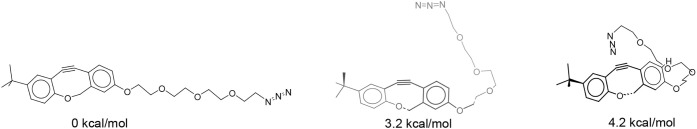
DFT B3LYP/6-311G+d,p optimized geometries and relative energies of three conformations of ODIBO-EG_4_-N_3_ (**7a**).

**Figure 12:**
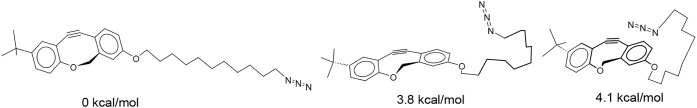
DFT B3LYP/6-311G+d,p optimized geometries and relative energies of three conformations ODIBO-O­(CH_2_)_11_-N_3_ (**13**).

SPAAC cyclization of ODIBO-EG_4_-N_3_ (**7a**) can produce two isomeric macrocycles: head-to-tail adduct **8a**(**ht**), where the substituent on the triazole nitrogen atom is directed away from the *t*-butyl group, and head-to-head adduct **8a**(**hh**) with the opposite arrangement of substituents (*vide supra,* Figure 1). The isomer **8a**(**ht**), as expected, is lower in energy by almost 9 kcal mol^–1^ (Figure 13). While both macrocycles **8a** can exist in several conformations, the most stable geometries are presented in Figure 1.

**Figure 13:**
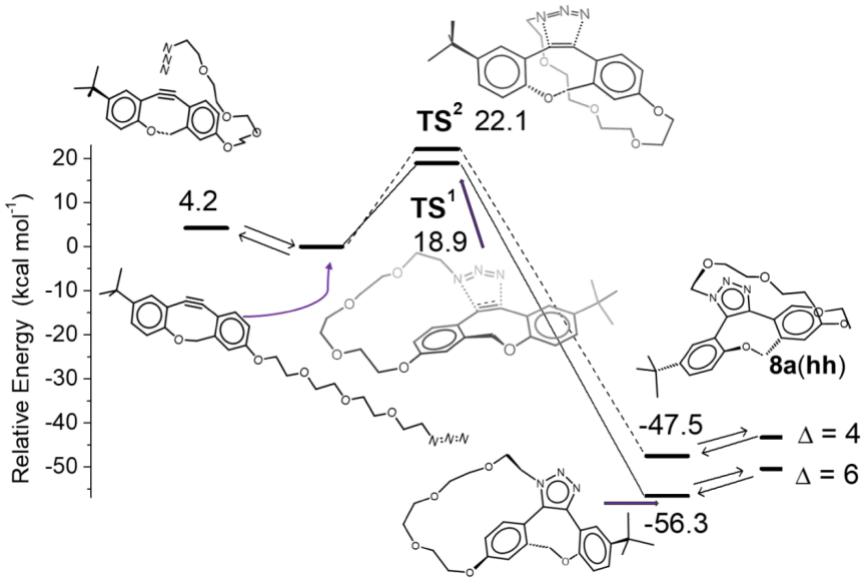
B3LYP/6-311+G­(d,p) energy diagram for the cyclization of ODIBO-EG4-N_3_ (**7a**).

The head-to-tail transition state (**TS** ^**1**^) leading to the formation of adduct **8a**(**ht**) is 3.2 kcal mol^–1^ lower than the head-to-head **TS** ^**2**^ leading to **8a**(**hh**). This difference corresponds to ∼200 times the difference in the rate of formation at 25 °C and agrees with the observation of a single product in the SPAAC cyclization of ODIBO-EG_4_-N_3_ (**7a**). The most stable conformation of the macrocycle **8a**(**ht**) is 6 kcal mol^–1^ lower in energy than the closest alternative (Figure 13). This result agrees with the absence of any evidence of conformational equilibria in the proton spectra of **8a**(**ht**).

The head-to-tail transition state **TS** ^**1**^ in SPAAC cyclization of ODIBO-O­(CH_2_)_11_-N_3_ (**13**) is 2.4 kcal mol^–1^ higher in energy than **TS** ^**1**^ for **7a**, which corresponds to the 56-fold rate reduction at 25 °C (Figure 14). This value nicely agrees with the experiment. The energy difference between **TS** ^**1**^ (leading to **14a**) and head-to-head transition state **TS** ^**2**^ (producing **14b**) is 3.2 kcal/mol, which is exactly the same as **the TS** ^**1**^ – **TS** ^**2**^ difference in ODIBO-EG_4_-N_3_ (**7**) cyclization (Figures 13 and 14). However, preparative photolysis of photo-ODIBO-O­(CH_2_)_11_-N_3_ (**12**) allowed for the isolation of both isomers **14a** and **14b** in an ∼7:2 ratio (*vide supra*). The formation of significant amounts of **14b** in the intramolecular SPAAC reaction of ODIBO-O­(CH_2_)_11_-N_3_ (**13**) suggests that the actual difference in activation barriers is smaller. It is interesting to note that the conformations of the macrocycle **14b** (2.9 kcal/mol) are closer in energy than in **8a**(**ht**) (5.9 kcal/mol), **8a**(**hh**) (4.2 kcal/mol), and **14a** (11 kcal/mol), potentially explaining why the variable-temperature proton spectra of **14b** indicate the presence of conformational equilibrium.^19^

**Figure 14:**
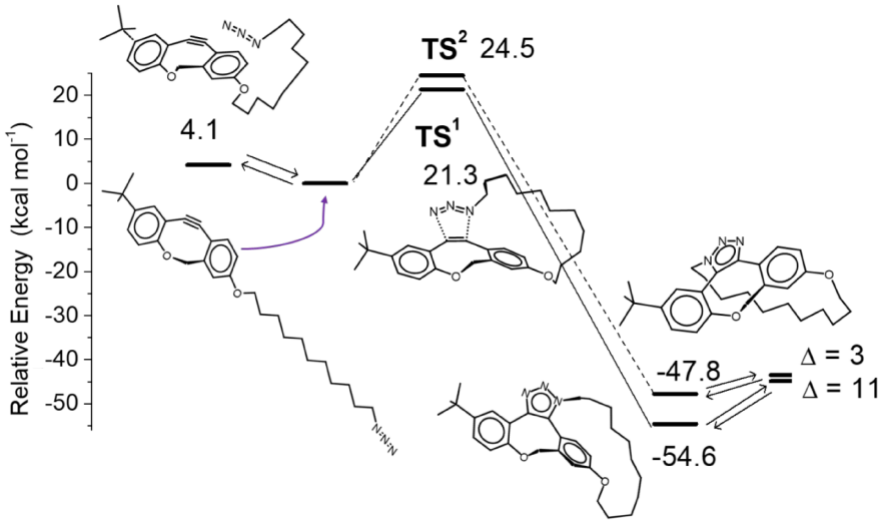
B3LYP/6-311+G­(d,p) energy diagram for the cyclization of ODIBO-O­(CH_2_)_11_-N_3_ (**13**).

Finally, the activation barrier for the dimerization of ODIBO-EG_4_-N_3_ (**7a**) was analyzed. Due to the size of the system, the geometry of the dimerization transition state (**TS**_**dim**_), starting material **7a**, and head-to-tail cyclization transition state **TS** ^**1**^ were optimized using a smaller set of orbitals, i.e., 6-31+G­(d). The single-point energies of the resulting geometries were calculated with the 6-311+G­(d) set. The activation barrier of the head-to-tail dimerization was 11.7 kcal/mol, while for head-to-head dimerization, the barrier was somewhat higher, 12.8 kcal/mol.

These DFT calculations predict that the activation energy for the dimerization is 7.0 kcal/mol lower than that of cyclization, **TS**_**dim**_ = 11.7 kcal/mol and **TS** ^**1**^ = 18.7 kcal mol^–1^. In MeOH, the difference grows by another 1 kcal mol^–1^. Qualitatively, these results agree with our experimental data, i.e., Δ*H* ^‡^ for dimerization is ∼1.5 kcal mol^–1^ and Δ*G* ^‡^ (25 °C) ∼2.5 kcal mol^–1^ is lower than for cyclization (Figures 7 and 8), but the experimental difference in activation energies is much smaller than predicted by DFT calculations (7.0 kcal/mol).

## Conclusions

Two efficient SPAAC-based, as well as photo-SPAAC-based, macrocyclization strategies are presented in this report. In the first method, a linear bifunctional macrocycle precursor equipped with a cyclopropenone-caged dibenzocyclooctyne moiety at one terminus and an azide group on the other is employed. Macrocyclization is induced by UVA–blue light irradiation, which causes the decarbonylation of the cyclopropenone group and the quantitative formation of dibenzocyclooctyne. The latter moiety reacts with the azide terminus, producing macrocycles in high to quantitative yields. No dimers or higher oligomers are observed in reaction mixtures. The macrocycles are produced in solution at concentrations from 10 μM to 0.12 M, as well as in the neat state. Performing reactions in the temperature range from 25 to 65 °C does not affect the outcome.

The rate of SPAAC macrocyclization is high; for ODIBO-based precursors in DCM at 25 °C, it can reach 0.007 M^–1^s^–1^ and is somewhat slower for DIBO analogs, up to 0.001 M^–1^s^–1^. The rate of SPAAC cyclization increases with solvent polarity. Thus, the reaction is ∼2 times faster in MeOH and ∼400 times faster in aqueous solutions. The effective molarity for these compounds in methanol solution can be estimated at EM ∼0.1 M or above. With this photo-SPAAC method, 18- to 51-membered macrocycles have been prepared. SPAAC cyclization pathways were analyzed at the DFT level of theory.

An alternative approach to the preparation of macrocycles is based on the double-SPAAC reaction or SPAAC-photo-SPAAC reaction sequence, where monobifunctional dibenzocyclooctadiyne or bis-dibenzocyclooctynes react with bis-azides. This method also produces a high yield of macrocycles. Small amounts of dimeric cyclic byproducts have been detected in some cases. Macrocycles with 18- to 34-membered rings have been prepared with the double-SPAAC method.

## Supplementary Material



## Notes

Experimental procedures, synthesis and characterization of compounds, relevant mass and UV spectra, kinetic study data, and the details of DFT calculations (PDF)
