# Supplementary material for: Synthesis of Macrocyclic Structures by Intramolecular Photoclick Reaction
Source: J Am Chem Soc. 2026 Jul 22;148(30):32461–72. doi: 10.1021/jacs.6c09335 (PMC13449954; doi:10.1021/jacs.6c09335)
Supplement: Supplementary file 1 [file ja6c09335_si_001.pdf]

Supporting Information

Synthesis of Macrocyclic Structures by Intramolecular Photo-Click Reaction

Shubham Sharma, Patrick Foster, Christopher Molnar, Ronaldo Badenhorst, Sergiy Minko, Vladimir V. Popik*

Department of Chemistry, University of Georgia, GA 30602, USA

vpopik@uga.edu

Table of contents:

Experimental……………..……………………..……………………...………………………………..S2

Kinetic measurement ………...…………………………………………………………………S3

Materials………………………...……………………………………………………………………..S5

Theoretical Procedures ………...………………………………………………………………S22

Experimental

DCM, DMF, and THF were dried over anhydrous sodium sulfate and purified by passing through the alumina column before use. HPLC-grade hexanes, ethyl acetate, and methanol were dried over anhydrous sodium sulfate or 4Å molecular sieves. Molecular biology grade absolute ethanol has been employed for kinetic studies. Flash chromatography was performed using 40-63 μm silica gel. Anhydrous sodium sulfate has been employed for drying in work-up procedures. All NMR spectra were recorded in $CDCl_3$ using 400 MHz instrument and chemical shift were referenced to TMS unless otherwise noted. Solutions for photochemical reactions were prepared using HPLC grade water and acetonitrile. Electronic spectra and scanning kinetics were recorded using Cary 60 UV-Vis spectrometer.

Photolytic experiments. Analytical photolyses were performed by the irradiation of ca. 10^{-4} M solutions of cyclopropenones in 1 cm fluorescent quartz cells using Luzchem LZC-4 photochemical reactor equipped with 8 (18 W) fluorescent UV lamps (350 nm)[1] or 340 nm LED (0.6 μW/mm^2) under constant stirring. The photolysates were treated with an excess of sodium azide in methanol. Preparative photolyses were conducted by irradiating methanol or DCM solutions of c.a. 50-100 mg of cyclopropenones in a quartz vessel equipped with a stirrer and an immersible cooling finger using 16 (18 W, 350 nm) lamps in the Rayonet photochemical reactor.

Photolyses in emulsion. 50 – 100 μL of 0.12 M corresponding cyclopropenone in dichloromethane was injected in 2-10 mL of rapidly stirred brine creating an emulsion, where the concentration of cyclopropenone within the droplets of DCM was 0.12 M, but the total amount of it in the cuvette was equal to 6 - 0.6 mM solution. The rapidly stirred emulsion was irradiated for 30 s with 8 (18W) 350 nm fluorescent bulbs. After incubation in the dark, 3 mL of DCM was added, organic layer separated, solvent removed in vacuum at r.t., the residue re-dissolved in methanol, and an excess of sodium azide added.

Irradiation in the neat form. A microscope glass slide was spin-coated in the dark with a thin layer of 1 - 10 mM DCM solution of the substrate. The solvent was let to evaporate at r.t. in the dark. The UV spectrum of the resulting thin layer was measured to ensure that the optical density of the layer at 350 nm is between 0.4 and 0.7. The slide was then placed on the rotating platform in the Luzchem photoreactor and irradiated from the top with four 350 nm fluorescent UV lamps for 30 s at r.t. The slide was incubated in the dark for 4 h and washed with 5 mL of methanol. Approximately 1 mg of sodium azide was added to the photolysate, which was subsequently concentrated and submitted to ESI MS analysis.

Kinetic Measurements

Rate measurements were performed using Carry-300 Bio UV-Vis spectrometer equipped with a Peltier temperature-controlled cell holder at 25 ± 0.1 ^{0}C. Reactions were monitored by following the decay of the characteristic 324 nm absorbance band of either the ODIBO or DIBO chromophore. Observed first-order rate constants were calculated by least-squares fitting of a single exponential function. Rates of cyclization of **7**, and **13**, as well as reaction of **6** with **18**, were determined in absolute ethanol at various temperatures using Shimadzu UV 2600 spectrometer equipped with a Peltier temperature-controlled cell holder (Tables S1-3). The temperature was controlled with 0.1 ^{0}C accuracy.

Table S1. Rate data for the cyclization ODIBO-EG$_4$-N$_3$ (**7**) in absolute ethanol at various temperatures

Temperature / ^{0}C	k_{obs} / s^{-1}
15	0.0100, 0.0117, 0.0124
25	0.0185, 0.0188, 0.0187
35	0.0292, 0.0291, 0.0293
45	0.0435, 0.0420, 0.0435
55	0.0827, 0.0802, 0.0785
65	0.117, 0.106, 0.112

Table S2. Rate data for the cyclization ODIBO-O(CH$_2$)$_{11}$-N$_3$ (**13**) in absolute ethanol at various temperatures

Temperature / ^{0}C	k_{obs} / 10^{-4} s^{-1}
15	1.31, 1.35, 1.31
25	3.00, 3.02, 2.99
35	6.07, 6.10, 6.12
45	11.8, 11.8, 11.8
55	21.2, 20.7, 21.1
65	35.2, 35.6, 35.3

Table S3. Rate data for the reaction of photo-ODIBO-EG$_4$-N$_3$ (**6**, 0.2 mM) with ODIBO-EG$_4$-Ts (**18**, 0.02 mM) in absolute ethanol at various temperatures

Temperature / ^{0}C	k_{obs} / 10^{-4} s^{-1}
15	3.17, 3.40, 3.44
25	4.58, 4.44, 4.47, 4.48
35	6.81, 6.85, 7.10
45	10.4, 11.3, 9.82
55	17.5, 17.0, 18.0
65	25.4, 23.9, 28.6

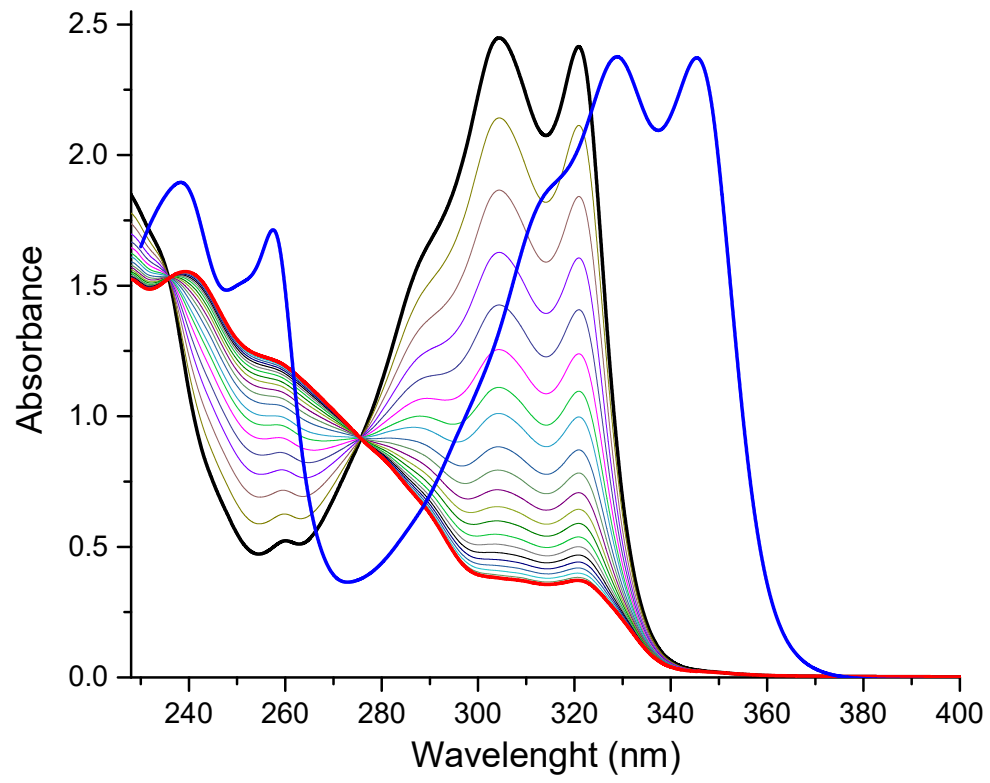

Figure S1. Changes in UV spectra during 350 nm irradiation of 50 μM DCM solution of photo-DIBO-EG$_4$-N$_3$ (**9b**).

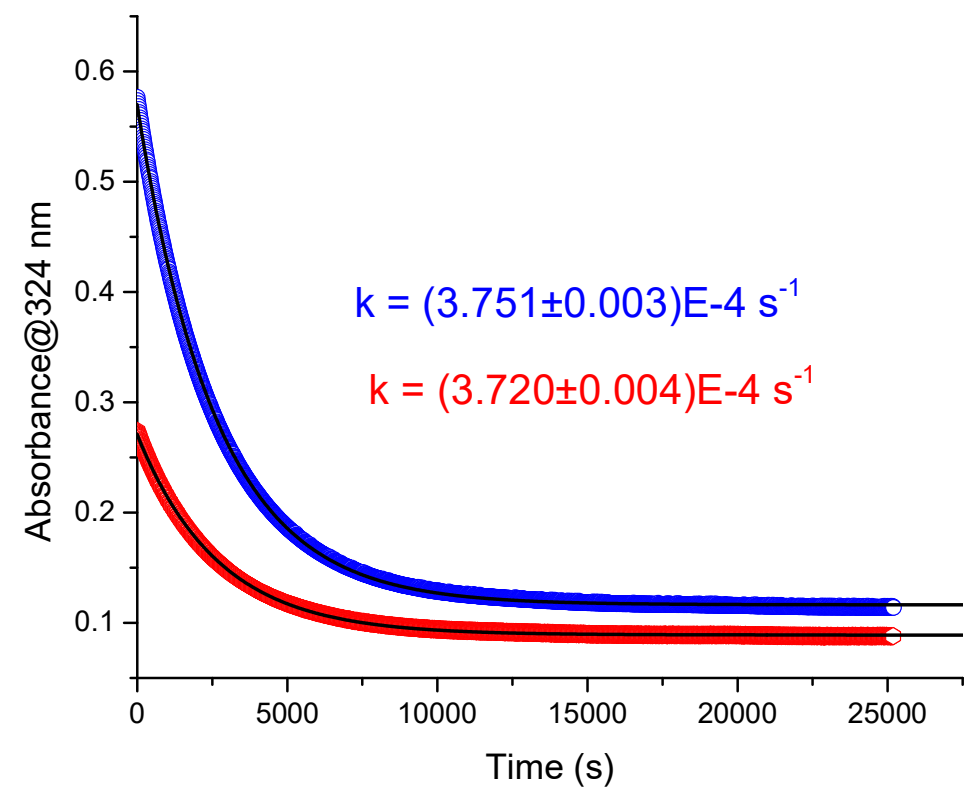

Figure S2. Decay of absorption at 324 nm of DIBO-EG$_4$-N$_3$ (**10b**) in DCM in the photolysate of (**9b**) at 10 μM (red) and 20 μM (blue) of starting material at 25±0.1 ^{0}C.

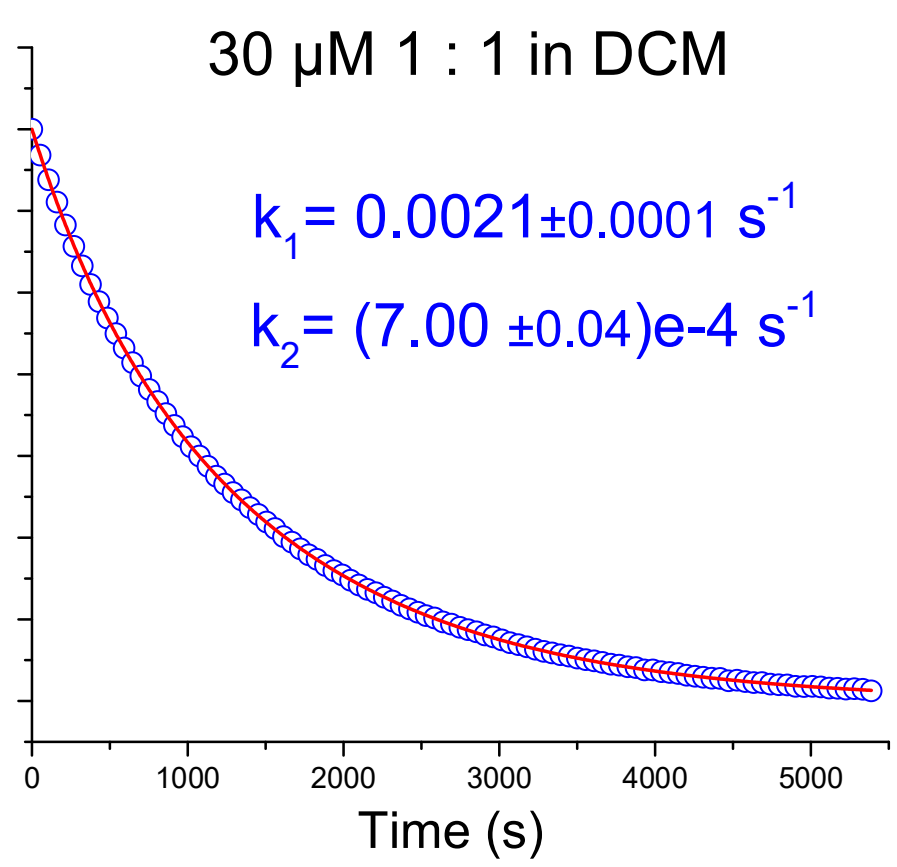

Figure S3. Decay of absorption at 264 nm in reaction of DIBOT-EG$_4$-DIBOT (**32**) in DCM in the photolysate of 30 μM of **31** in the presence of 30 μM of bis-azide (**25**) at 25±0.1 ^{0}C.

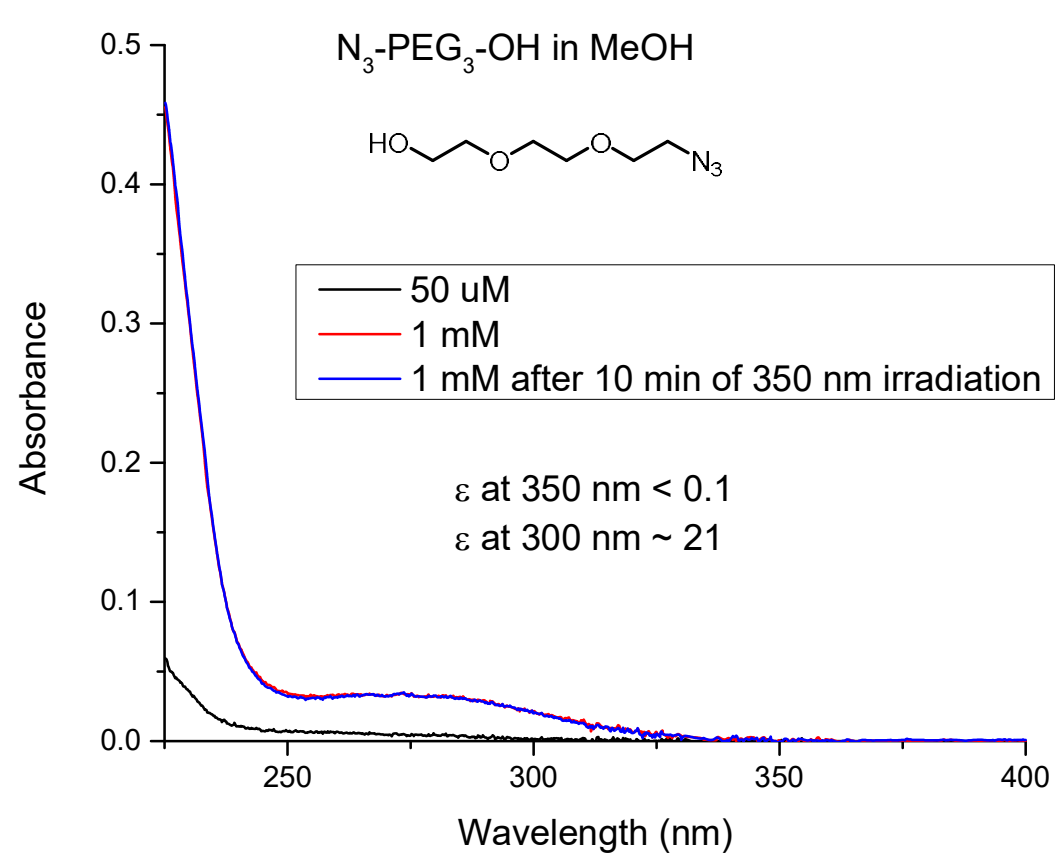

Changes in UV spectra after 10 min of 350 nm irradiation of 1 mM MeOH solution of EG_3-N_3

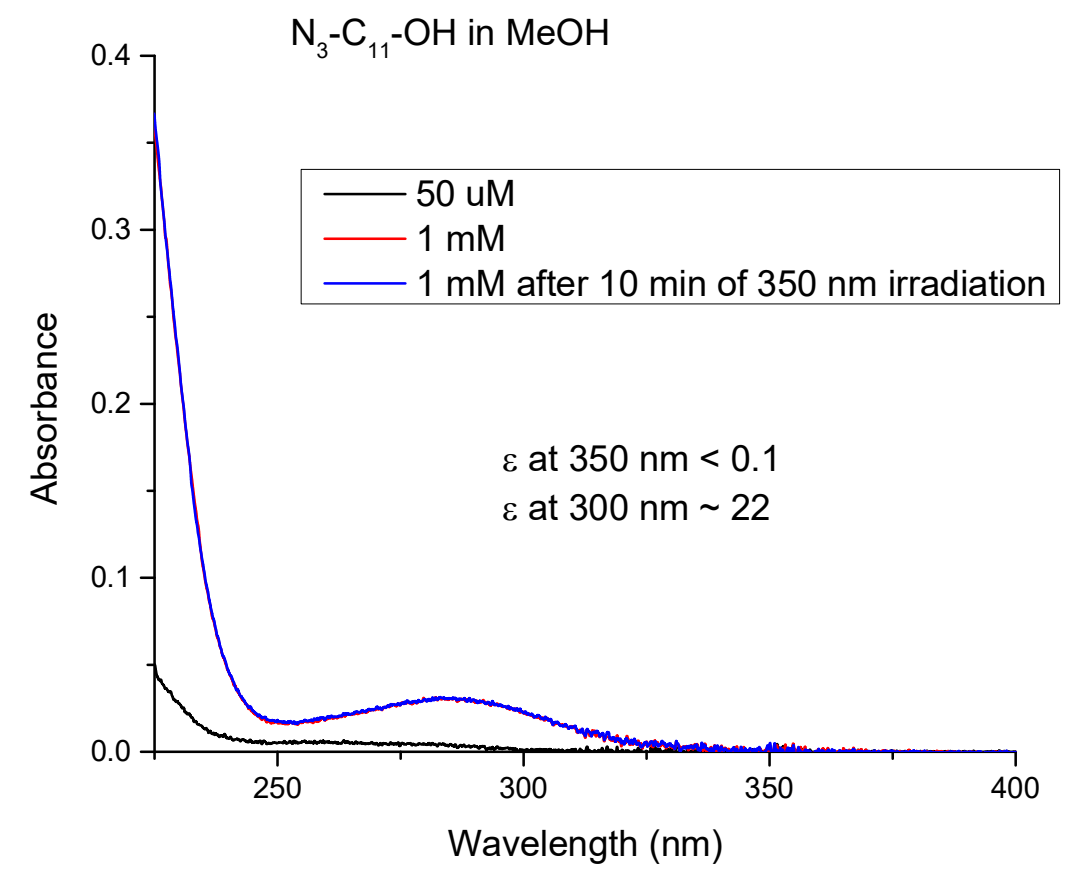

Changes in UV spectra after to min of 350 nm irradiation of 1 mM MeOH solution of HO-$(CH_2)_{11}$-N_3

Materials

Photo-ODIBO-OH (**4**)[2], photo-DIBO-OH (**34**),[3] photo-DIBO-EG-OH (XX),[4] photo-DIBOD (**23**),[5] ODIBO-OH (**15**),[6] and MC-DIBOD (**28**)[7] were prepared using previously reported procedures. Other reagents were obtained from commercial sources and used as received unless noted.

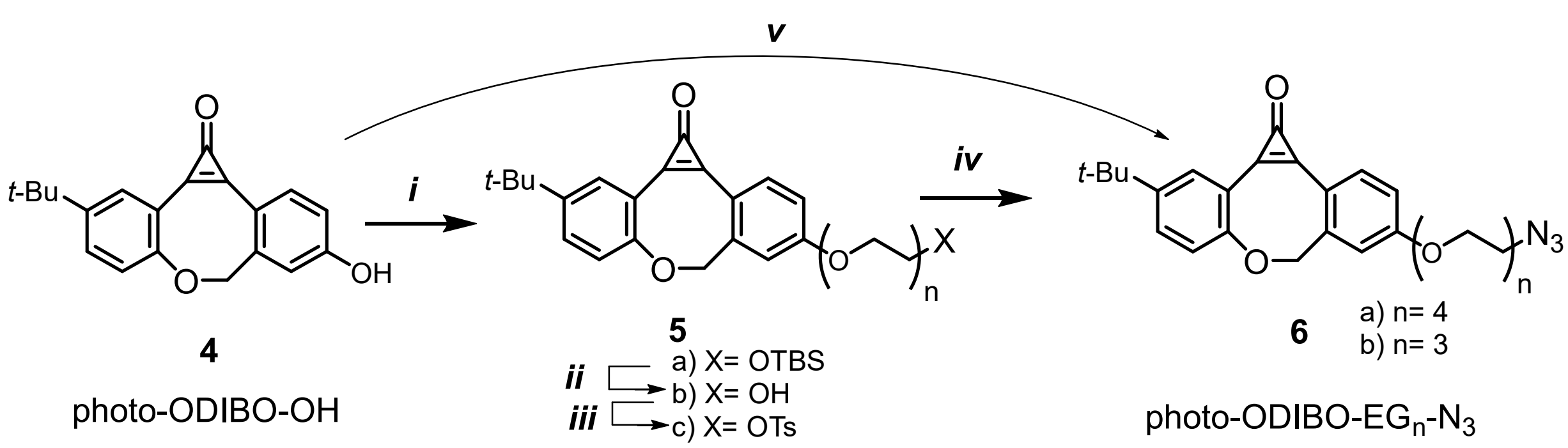

3-(tert-butyl)-9-((2,2,3,3-tetramethyl-4,7,10,13-tetraoxa-3-silapentadecan-15-yl)oxy) dibenzo[b,f]cyclopropa[d]oxocin-1(7H)-one (**photo-ODIBO-EG$_4$-OTBDMS**, **5a**).[8] Potassium carbonate (0.166 g, 1.2 mmol) was added to a solution of photo-ODIBO-OH (**4**, 0.1867 g, 0.6 mmol) and TsO-EG$_4$-OTBDMS (0.560 g, 1.2 mmol) in DMF (8 mL) and the reaction mixture was stirred for 4 h at 80 °C, cooled to room temperature, diluted with ethyl acetate (150 mL), washed with water (75 mL x 5) then brine (50 mL x 3), dried over anhydrous sodium sulfate, and concentrated in vacuo. The residue was purified by chromatography (ethyl acetate: hexanes 3:1) to afford **5a** (0.3111 g, 87%) as a faint yellow oil.

^1H NMR δ 7.91 (d, 2H), 7.48 (d, 1H), 7.18 (d, 1H), 7.03 (m, 2H), 5.24 (d, 1H), 4.76 (d, 1H), 4.20 (t, 2H), 3.86 (t, 2H), 3.74 (m, 4H), 3.62 (m, 6H), 3.52 (t, 2H), 1.32 (s, 9H), 0.85 (s, 9H), 0.02 (s, 6H).

$^{13}C\{^1H\}$ NMR δ 162.0, 160.4, 152.6, 147.9, 144.1, 142.3, 140.7, 135.4, 131.0, 130.6, 121.9, 117.9, 117.4, 117.1, 114.7, 78.7, 72.7, 70.99, 70.79, 70.77, 70.71, 69.5, 67.9, 62.8, 34.6, 31.4, 25.9, 18.4, -5.2.

3-(tert-butyl)-9-(2-(2-(2-(2-(hydroxyethoxy)ethoxy)ethoxy)ethoxy)dibenzo[b,f]-cyclopropa[d]oxocin-1(7H)-one (**photo-ODIBO-EG$_4$-OH**, **5b**).[8] 50% aqueous HF (0.0168 g, 0.84 mmol) was added to a solution of **5a** (0.250 g, 0.419 mmol) in CH_3CN (8 mL) and stirred for 15 minutes. The reaction mixture was quenched with $NaHCO_{3(aq)}$, extracted with ethyl acetate (50 mL x 3), washed with water (50 mL x 3) and brine (50 mL x 3), dried over anhydrous sodium sulfate and concentrated in vacuo. The residue was run through a silica plug (ethyl acetate) to afford **5b** (0.180 g, 89%) as a colorless oil.

^1H NMR δ 7.87 (d, 2H), 7.45 (d, 1H), 7.14 (d, 1H), 7.00 (m, 2H), 5.21 (d, 1H) 4.71 (d, 1H), 4.17 (t, 2H), 3.82 (d, 2H), 3.66 (m, 4H), 3.60 (m, 6H), 3.54 (t, 2H), 2.97 (s, 1H), 1.28 (s, 9H)

$^{13}C\{^1H\}$ NMR δ 161.9, 160.3, 152.6, 147.9, 143.9, 142.1, 140.6, 135.3, 130.9, 130.6, 121.9, 117.7, 117.3, 116.9, 114.6, 78.6, 72.5, 70.8, 70.6, 70.5, 70.3, 69.4, 67.9, 61.6, 34.5, 31.3.

2-(2-(2-(2-((3-(tert-butyl)-1-oxo-1,7-dihydrodibenzo[b,f]cyclopropa[d]oxocin-9-yl)oxy) ethoxy)ethoxy)ethoxy)ethyl 4-methylbenzenesulfonate (**photo-ODIBO-EG$_4$-OTos**, **5c**).[8]

Triethylamine (0.069 g, 0.68 mmol) was added to a stirring solution of p-toluene sulfonyl chloride (0.13 g, 0.68 mmol) and **5b** (0.218 g, 0.45 mmol) in CH_2Cl_2 (10 mL). The reaction mixture was stirred for 12 h, concentrated in vacuo and purified by chromatography (methanol: chloroform 1:20) to afford **5c** (0.248 g, 86%) as a colorless oil.

^1H NMR δ 7.94 (d, 2H), 7.79 (d, 2H), 7.51 (d, 1H), 7.33 (d, 2H), 7.21 (d, 1H), 7.06 (m, 2H), 5.28 (d, 1H), 4.79 (d, 1H), 4.23 (t, 2H), 4.15 (t, 2H), 3.89 (t, 2H), 3.68 (m, 6H), 3.59 (s, 4H), 2.42 (s, 3H), 1.35 (s, 9H)

$^{13}C\{^1H\}$ NMR δ 162.1, 160.5, 152.8, 148.1, 144.9, 144.1, 142.4, 140.8, 135.5, 133.2, 131.2, 130.7, 129.9, 128.1, 122.1, 117.9, 117.5, 117.1, 114.8, 78.8, 71.0, 70.9, 70.8, 70.7, 69.6, 69.4, 68.8, 68.1, 34.7, 31.5, 21.7.

9-(2-(2-(2-(2-azidoethoxy)ethoxy)ethoxy)ethoxy)-3-(tert-butyl)dibenzo[b,f]cyclopropa[d] oxocin-1(7H)-one (**photo-ODIBO-EG$_4$-N$_3$**, **6a**).[8] Sodium azide (0.130 g, 2 mmol) was added to

a stirring solution of **5c** (0.248 g, 0.39 mmol) in DMF (10 mL). The reaction mixture was stirred for 36 h, filtered and concentrated in vacuo. The residue was purified by chromatography (ethyl acetate: hexanes 1:2) to afford **6** (0.112 g, 57%) as a faint yellow oil.

^{1}H NMR (900 MHz, DMF) δ 7.86 (m, 2H), 7.43 (dd, 1H), 7.13 (d, 1H), 7.00 (d, 1H), 6.97 (dd, 1H), 5.19 (d, 1H), 4.71 (d, 1H), 4.16 (m, 2H), 3.82 (m, 2H), 3.66 (m, 2H), 3,62 (m, 2H), 3.59 (m, 6H), 3.30 (m, 2H), 1.28 (s, 9H).

^{13}C{^{1}H} NMR (226 MHz, DMF) δ 161.9, 160.3, 152.5, 147.8, 143.9, 142.1, 140.6, 135.3, 130.9, 130.6, 121.9, 117.7, 117.2, 116.9, 114.5, 78.6, 70.8, 70.62, 70.59, 70.57, 69.9, 69.4, 67.9, 50.6, 34.5, 31.2.

HRMS (ESI/ Orbitrap), *m/z*: $[M+H]^+$ Calcd for $C_{28}H_{34}N_3O_6^+$ 508.2442; Found 508.2447.

Macrocycle 8a. A solution of photo-ODIBO-EG$_4$-N$_3$ (0.053 g, 0.104 mmol) in DCM (220 mL) was irradiated for 10 min with stirring and incubated overnight at room temperature in the dark. The reaction mixture was concentrated in vacuo and purified by chromatography (methanol: chloroform 1:40) to afford 8 (0.047 g, 95%) as a colorless oil.

^{1}H NMR δ 7.65 (d, 1H), 7.36 (d, 1H), 7.25 (d, 1H), 7.23 (dd, 1H), 7.09 (dd, 1H), 6.87 (d, 1H), 4.70 (m, 2H), 4.51 (br, 2H), 3.80 (m, 2H), 3.69 (m, 2H), 3.51 (s, 4H), 3.44 (m, 2H), 3.19 (m, 4H), 1.31 (s, 9H).

^{13}C{^{1}H} NMR δ 160.2, 152.3, 143.9, 137.6, 133.5, 130.0, 129.1, 127.0, 120.4, 120.0, 117.9, 117.7, 116.5, 113.1, 77.4, 72.1, 71.4, 71.0, 70.3, 70.2, 70.0, 67.1, 48.3, 37.3, 29.9.

HRMS (ESI/ Orbitrap), *m/z*: $[M+H]^+$ Calcd for $C_{27}H_{34}N_3O_5^+$ 480.2493; Found 480.2471

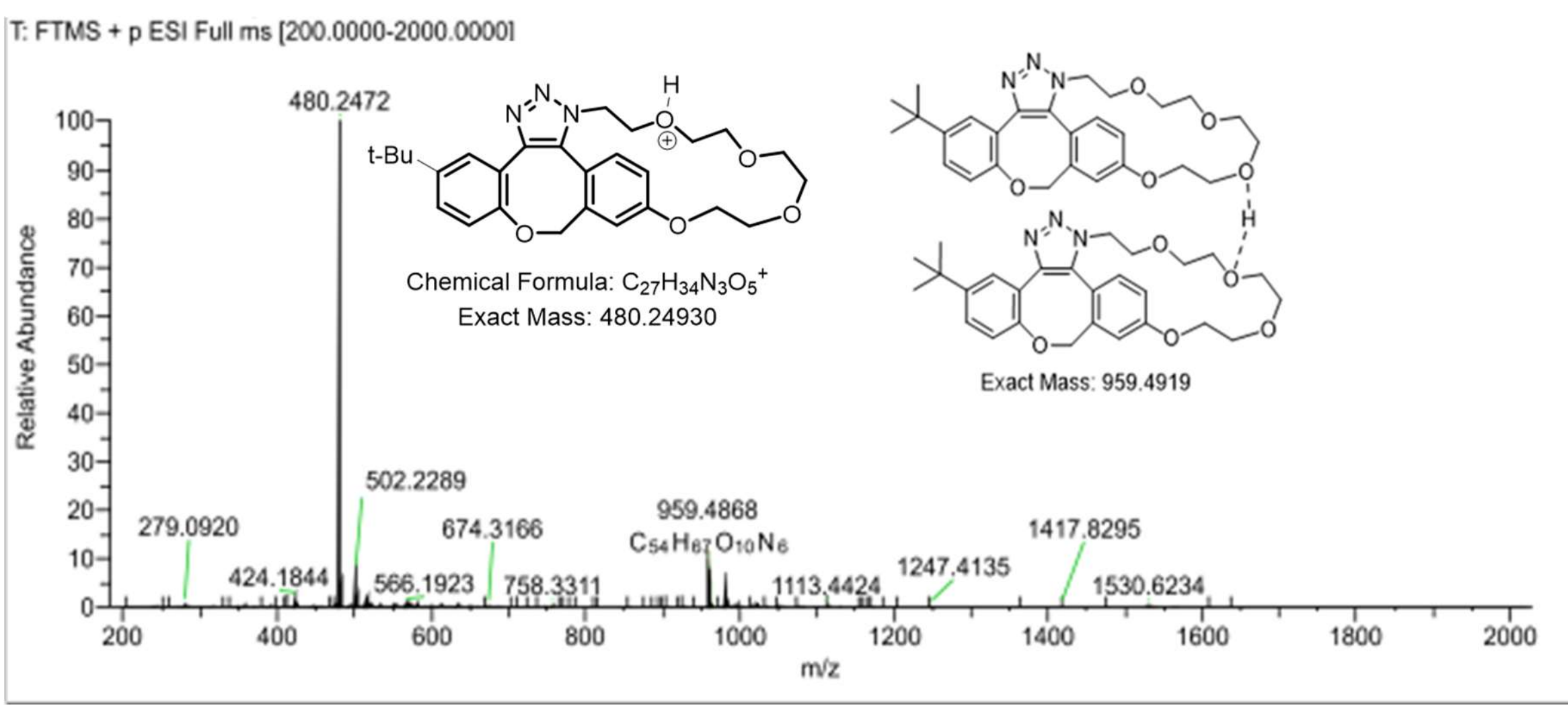

Figure S4. Mass spectrum of the photolysate of **6a** in the neat form with NaN$_3$ post-photolysis treatment.

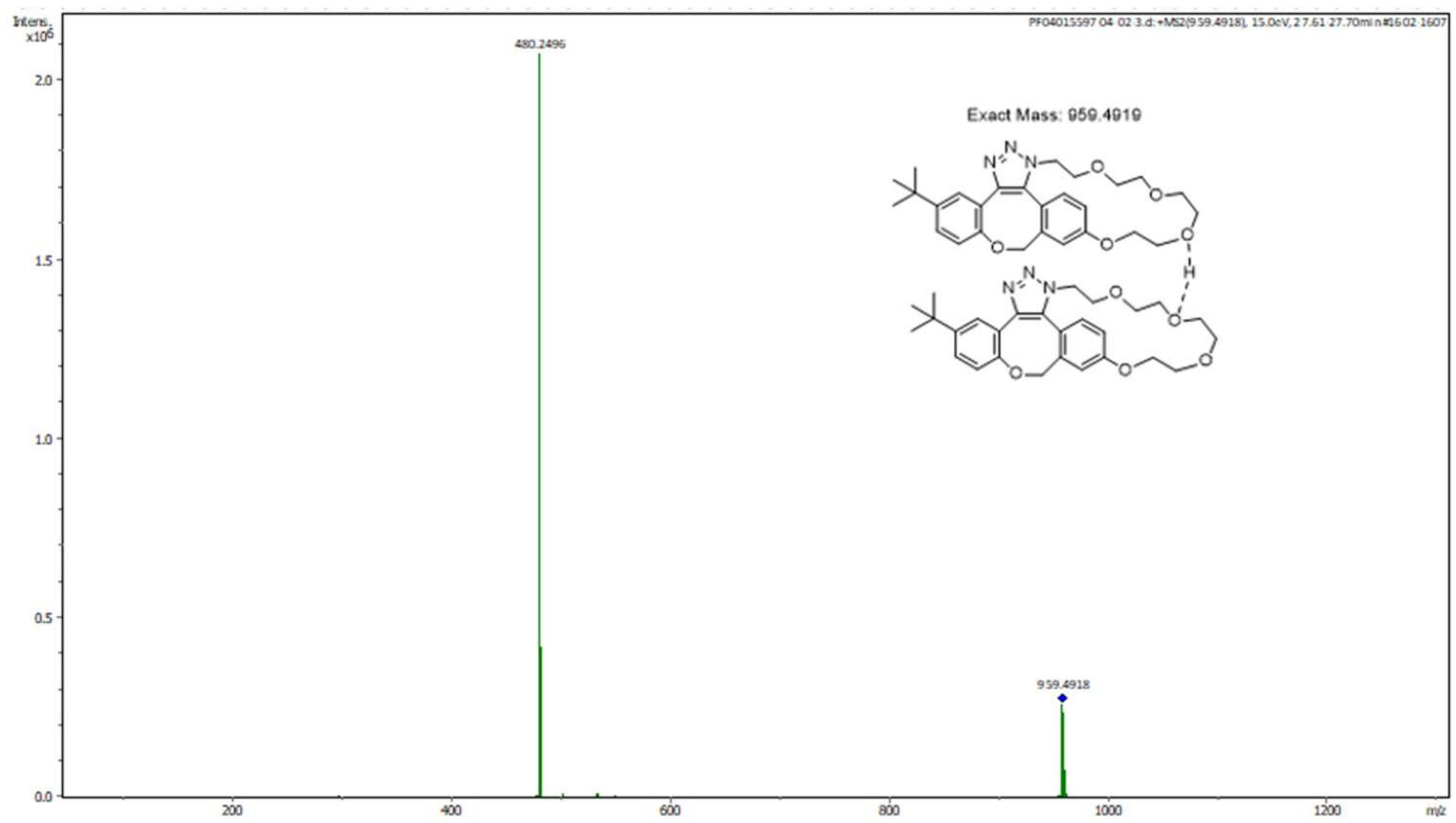

Figure S5. ESI MS-MS (15 eV) spectrum of 959 m/z.

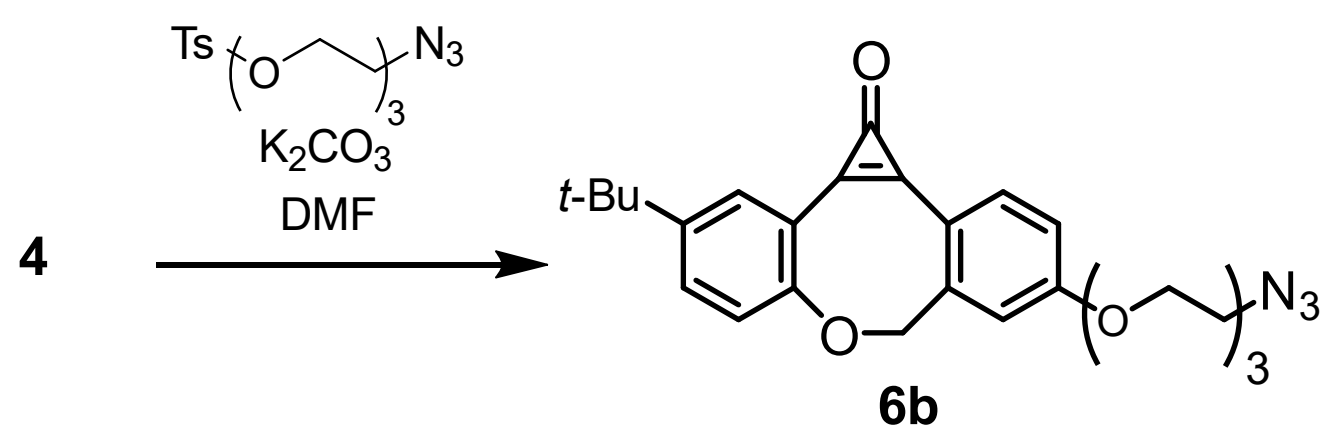

9-(2-azidoethoxy)-3-(tert-butyl)dibenzo[*b,f*]cyclopropa[*d*]oxocin-1(7*H*)-one (photo-ODIBO-EG_3N_3**, 6b**). 2-(2-(2-azidoethoxy)ethoxy)ethyl tosylate (92 mg, 0.25 mmol), photo-ODIBO-OH (**4**, 50 mg, 0.16 mmol), and potassium carbonate (45 mg, 0.32 mmol) were dissolved in DMF (2.5 mL) and heated for 5 hours at 80 ^{o}C. The reaction mixture was diluted with 10 mL ethyl acetate and washed with brine, dried over anhydrous magnesium sulphate and concentrated. Crude product was purified by chromatography (20-60% ethyl acetate in hexane) to give 29 mg (40%) of **6b** as a yellow oil.

^{1}H NMR δ 7.93 (m, 2H), 7.49 (d, J = 8 Hz, 1H), 7.20 (d, J = 8 Hz, 1H), 7.05 (m, 2H), 5.27 (d, J = 9 Hz, 1H), 4.77 (d, J = 9 Hz, 1H), 4.23 (m, 2H), 3.92 (m, 2H), 3.76 – 3.65 (m, 6H), 3.38 (t, J = 7 Hz, 2H), 1.34 (s, 9H).

^{13}C {^{1}H} NMR δ 162.0, 160.5, 153.0, 153.8, 148.0, 135.5, 131.1, 130.7, 129.8, 128.0, 122.0, 117.9, 117.4, 117.0, 114.6, 78.8, 71.0, 70.8, 70.2, 69.6, 67.9, 50.8, 34.7, 31.42.

HRMS (ESI/ Orbitrap), *m/z*: $[M+H]^+$ Calcd for $C_{26}H_{30}N_3O_5^+$ 464.2180; Found 464.2168

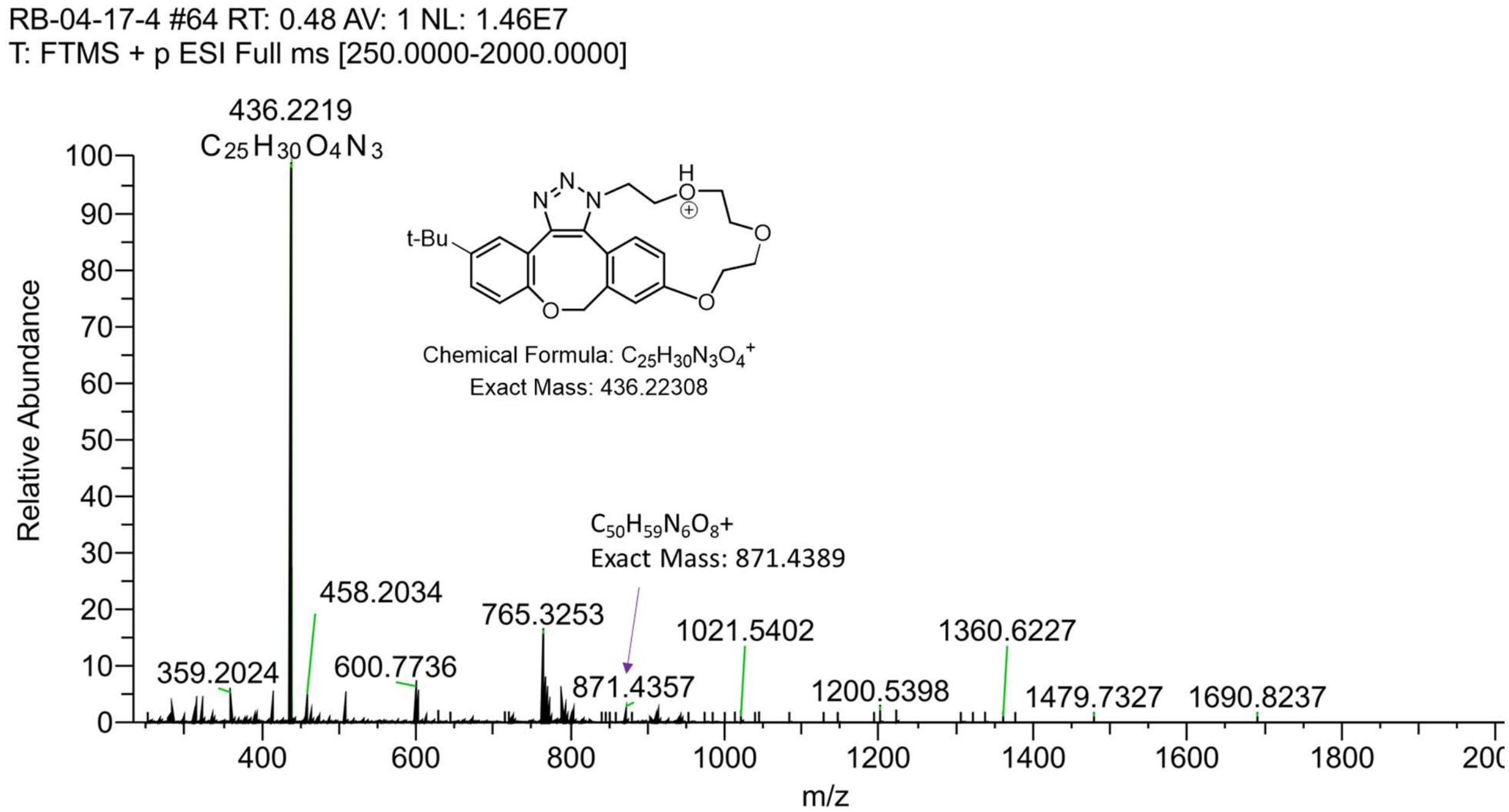

Figure S6a. Macrocycle **8b** from the photolysate of photo-ODIBO-EG$_3$-N$_3$(**6b**).

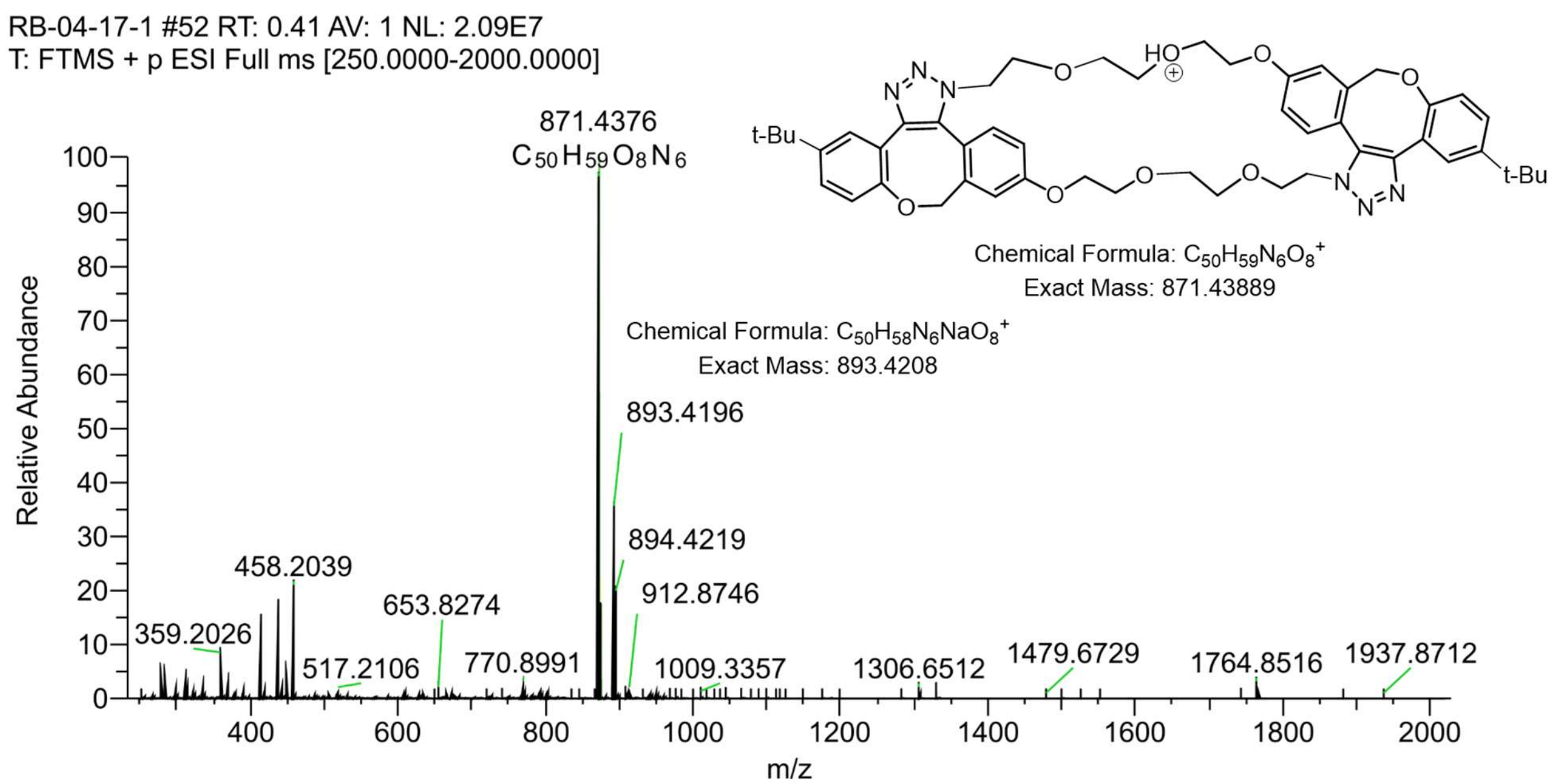

Figure S6b. One of the cyclic dimers **7b-dimer** from the photolysate of photo-ODIBO-EG$_3$-N$_3$(**6b**).

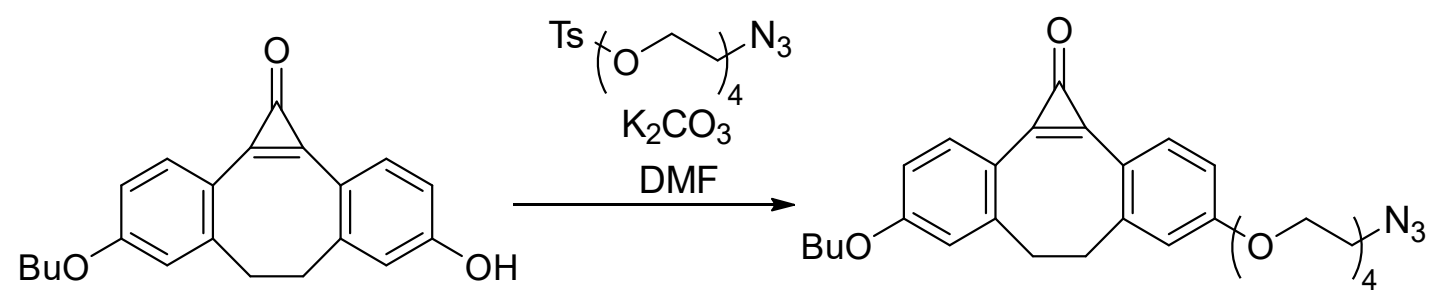

34 9a

4-(2-(2-(2-(2-azidoethoxy)ethoxy)ethoxy)ethoxy)-9-butoxy-6,7-dihydro-1H-dibenzo[a,e] cyclopropa[c][8]annulen-1-one (photo-DIBO-EG$_4$-N$_3$, **9a**). 2-(2-(2-(2-azidoethoxy)ethoxy) ethoxy)ethyl tosylate (1.6 g, 4.3 mmol), photo-DIBO-OH (**34**, 812 mg, 2.53 mmol), and potassium carbonate (350 mg, 2.53 mmol) were dissolved in DMF (25 mL) and heated for 4 hours at 70 °C. The reaction mixture was diluted with 200 mL ethyl acetate and washed with brine, dried over anhydrous magnesium sulphate and concentrated. Crude product was purified by chromatography (20-60% ethyl acetate in hexane) to give 1.28 g (96%) of **9a** as a yellow waxy solid.

^{1}H NMR δ 7.93 (d, J = 8.6 Hz, 2H), 6.97 – 6.84 (m, 4H), 4.21 (dd, J = 5.6, 3.9 Hz, 2H), 4.04 (t, J = 6.5 Hz, 2H), 3.94 – 3.85 (m, 2H), 3.76 – 3.65 (m, 10H), 3.38 (t, J = 5.1 Hz, 2H), 3.33 (d, J = 10.6 Hz, 2H), 2.70 – 2.54 (m, 2H), 1.79 (m, 2H), 1.58 – 1.44 (m, 2H), 0.99 (t, J = 7.4 Hz, 3H).

^{13}C {^{1}H} NMR δ 162.6, 162.1, 161.6, 153.8, 147.8, 142.4, 142.0, 135.8, 135.7, 116.6, 116.4, 116.2, 116.2, 112.4, 112.3, 70.9, 70.7, 70.71, 70.68, 70.0, 69.5, 68.0, 67.7, 50.7, 37.2, 37.2, 31.2, 19.20, 13.8.

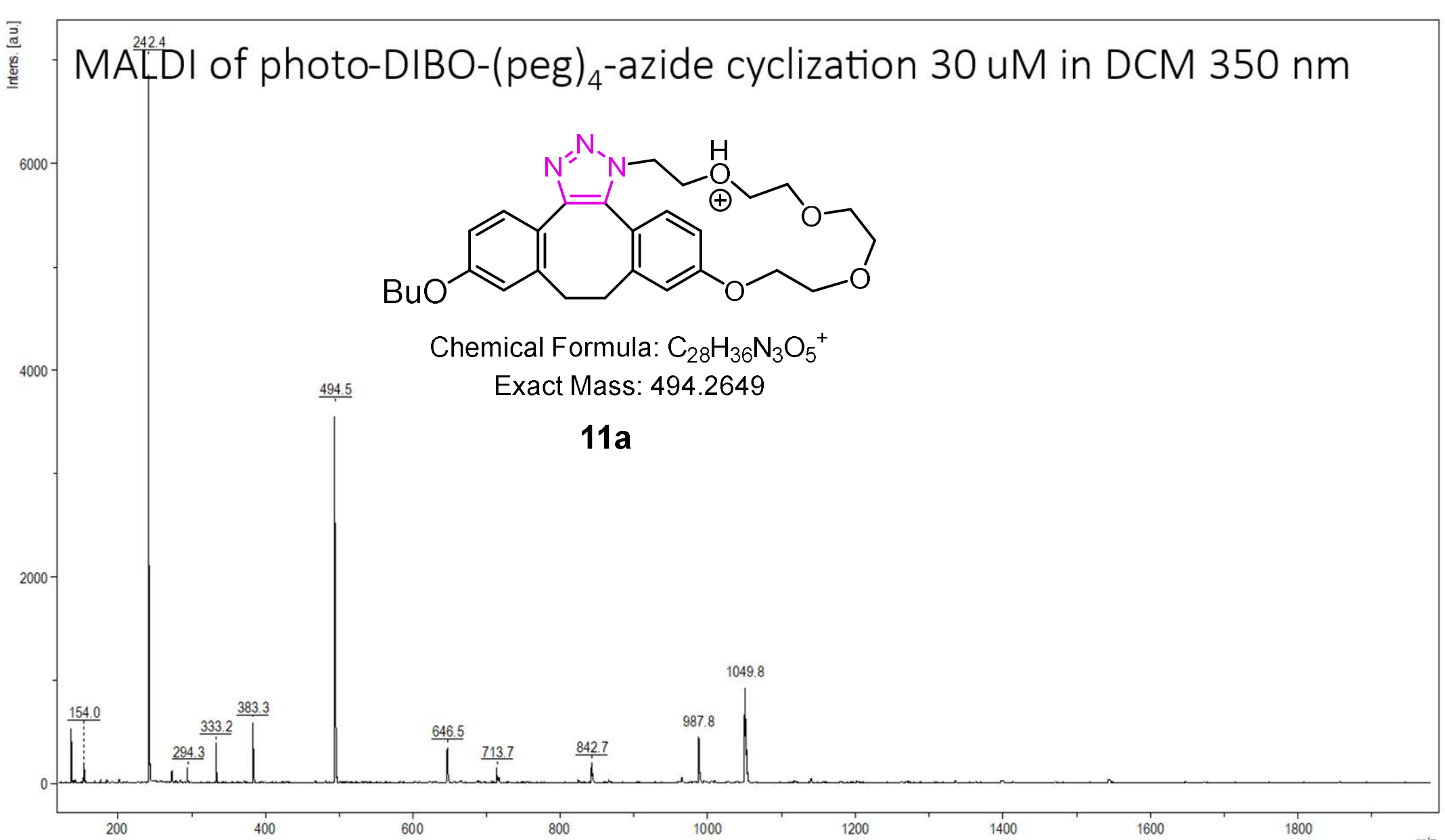

Figure S7a. ESI-mass spectrum of the photolysate of **9a** in DCM.

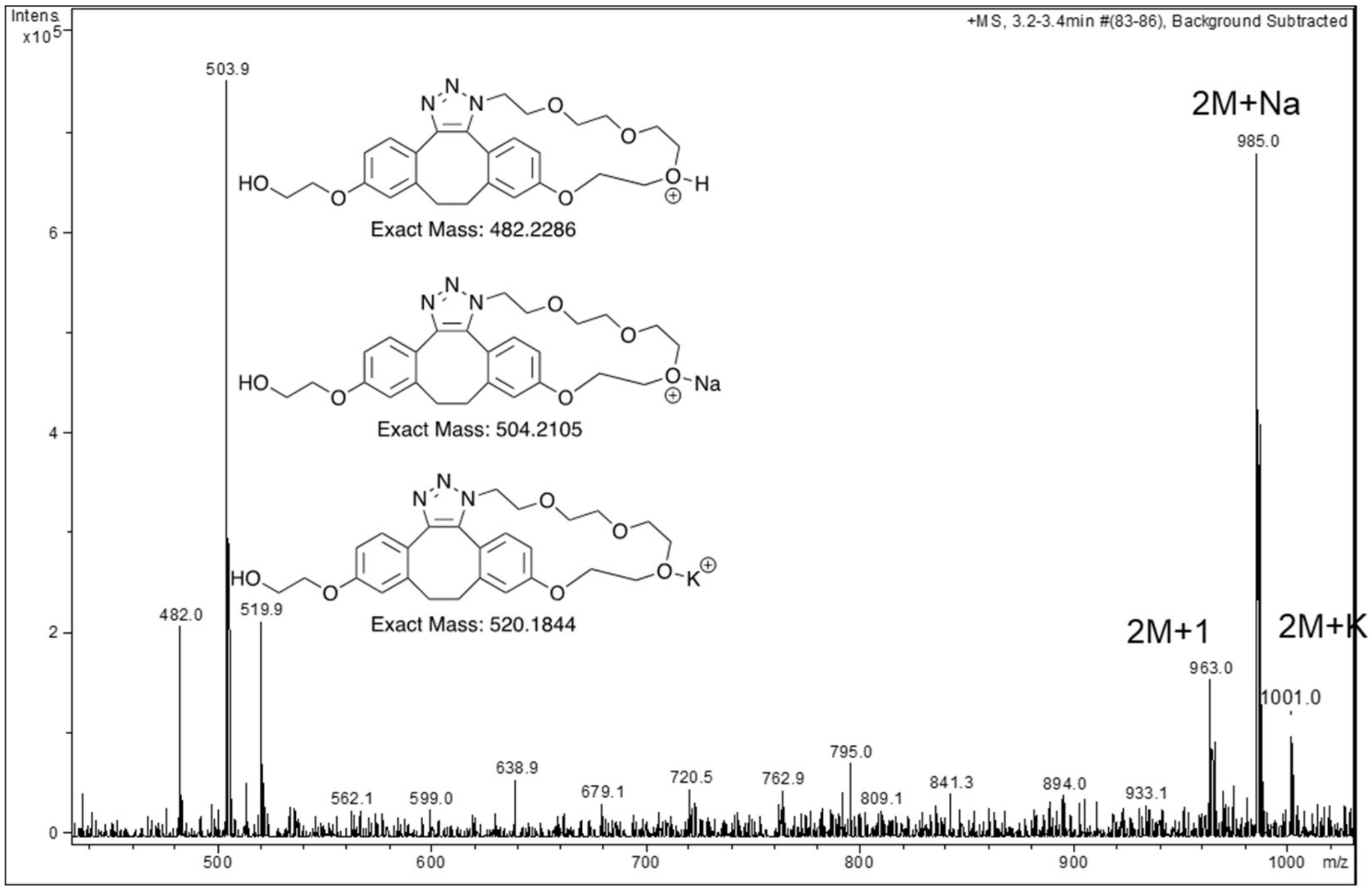

Figure Sb. ESI-mass spectrum of the photolysate of **9b** in DCM.

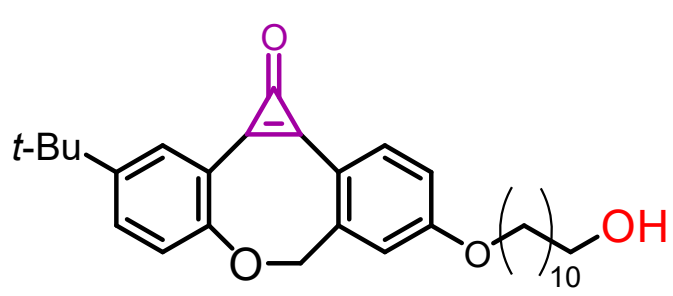

35

3-(tert-butyl)-9-((11-hydroxyundecyl)oxy)dibenzo[b,f]cyclopropa[d]oxocin-1(7H)-one (**Photo-ODIBO-O(CH_2)$_{11}$-OH**, **35**). A solution of 11-bromoundecan-1-ol (0.210 g, 0.84 mmol) in DMF (2 mL) was added dropwise to a stirring solution of K_2CO_3 (0.110 g, 0.796 mmol), potassium iodide (9 mg, 0.05 mmol) and photo-ODIBO-OH (**4**, 0.157 g, 0.51 mmol) in DMF (5 mL) and was stirred for 3 h at 80 °C. The reaction mixture was cooled to room temperature, diluted with water (100 mL), extracted with ethyl acetate (5 × 100 mL), washed with brine (100 mL), dried and concentrated in vacuo. The residue was purified by chromatography (ethyl acetate: hexanes 1:10) to afford **8** (0.091 g, 38%) as a slightly yellow oil.

^{1}H NMR δ 8.01 (d, 1H), 7.48 (m, 2H), 7.18 (d, 1H), 7.04 (m, 2H), 5.29 (d, 1H), 4.73 (d, 1H), 4.15 (m, 2H), 3.63 (t, 2H), 1.94 (m, 2H), 1.75 (bs, 1H), 1.55 (m, 4H), 1.35 (s, 9H), 1.29 (m, 12H).

^{13}C{^{1}H} NMR δ 160.51, 160.46, 152.7, 147.9, 144.7, 141.6, 139.9, 133.8, 131.7, 130.8, 122.1, 121.8, 116.7, 115.1, 113.4, 78.6, 69.3, 63.1, 34.7, 32.9, 31.5, 29.6, 29.56, 29.50, 29.4, 29.3, 25.9, 25.8.

HRMS (ESI/ Orbitrap), *m/z*: [M+H]$^+$ Calcd for $C_{31}H_{41}O_4^+$ 477.2999; Found 477.2984.

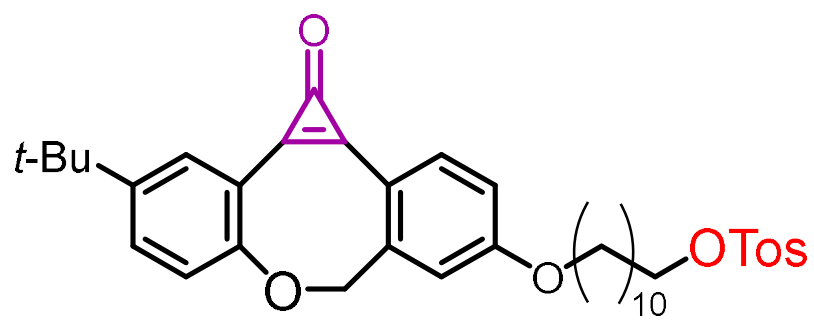

36

11-((3-(tert-butyl)-1-oxo-1,7-dihydrodibenzo[b,f]cyclopropa[d]oxocin-9-yl)oxy)undecyl 4-methylbenzenesulfonate (**photo-ODIBO-C_{11}-OTos**, **36**). Triethylamine (0.097 g, 0.95 mmol) was added to the stirred solution of p-toluenesulfonyl chloride (0.182 g, 0.95 mmol) and photo-ODIBO-C_{11}-OH (**35**, 0.091 g, 0.191 mmol) in CH_2Cl_2 (13 mL). The reaction mixture was stirred for 12 h, filtered, concentrated in vacuo and purified by chromatography (acetone: hexanes 1:20) to afford **36** (0.118 g, 98%) as a colorless oil.

^{1}H NMR δ 7.98 (d, 1H), 7.78 (d, 2H), 7.48 (m, 2H), 7.33 (d, 2H), 7.19 (d, 1H), 7.05 (t, 2H), 5.29 (d, 1H), 4.73 (d, 1H), 4.16 (m, 2H), 4.00 (t, 2H), 2.43 (s, 3H), 1.94 (m, 2H), 1.59 (m, 4H), 1.35 (s, 9H), 1.25 (m, 12H).

^{13}C{^{1}H} NMR δ 160.49, 160.46, 152.7, 147.9, 144.73, 144.71, 141.7, 139.9, 133.8, 133.4, 131.5, 130.8, 129.9, 128.0, 122.1, 121.8, 116.8, 115.1, 113.4, 78.6, 70.9, 69.3, 34.7, 31.5, 29.6, 29.5, 29.4, 29.3, 29.0, 28.9, 25.9, 25.4, 21.7.

HRMS (ESI/ Orbitrap), *m/z*: $[M+H]^+$ Calcd for $C_{38}H_{47}O_6S^+$ 631.3088; Found 631.3064.

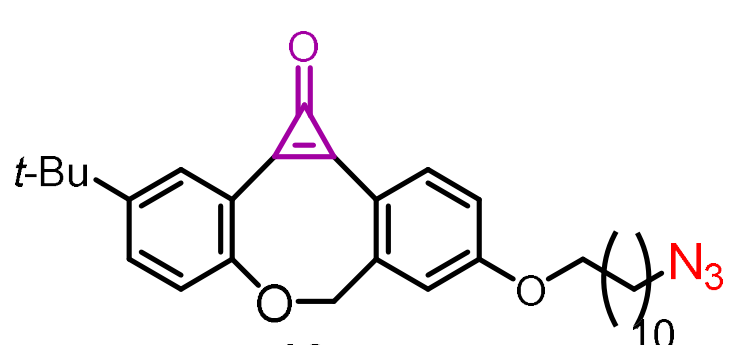

12

9-((11-azidoundecyl)oxy)-3-(tert-butyl)dibenzo[b,f]cyclopropa[d]oxocin-1(7H)-one (photo-ODIBO-C_{11}-N_3) (**12**). Sodium azide (0.050 g, 0.77 mmol) was added to a solution of photo-ODIBO-C_{11}-OTs (**36**, 0.089 g, 0.141 mmol) in DMF (5 mL) and stirred for 12 h at room temperature. The reaction mixture was diluted with ethyl acetate (250 mL), washed with brine (100 mL), water (5 × 100 mL), brine again (100 mL), dried and concentrated in vacuo. The residue was run through a silica plug (methanol: dichloromethane 1:20) to afford **12** (0.054 g, 76%) as a faint yellow oil.

^{1}H NMR δ 7.98 (d, 1H), 7.48 (m, 2H), 7.19 (d, 1H), 7.05 (t, 2H), 5.30 (d, 1H), 4.74 (d, 1H), 4.15 (m, 2H), 3.23 (t, 2H), 1.95 (m, 2H), 1.58 (m, 4H), 1.35 (s, 9H), 1.29 (m, 12H).

$^{13}C\{^1H\}$ NMR δ 160.51, 160.47, 152.7, 147.9, 144.8, 141.7, 139.9, 133.8, 131.6, 130.8, 122.1, 121.8, 116.8, 115.2, 113.4, 78.6, 69.3, 51.6, 34.7, 31.5, 29.64, 29.63, 29.57, 29.49, 29.3, 29.2, 28.9, 26.8, 25.9

HRMS (ESI/ Orbitrap), *m/z*: $[M+H]^+$ Calcd for $C_{31}H_{40}O_3^+$ 502.3046; Found 502.3046.

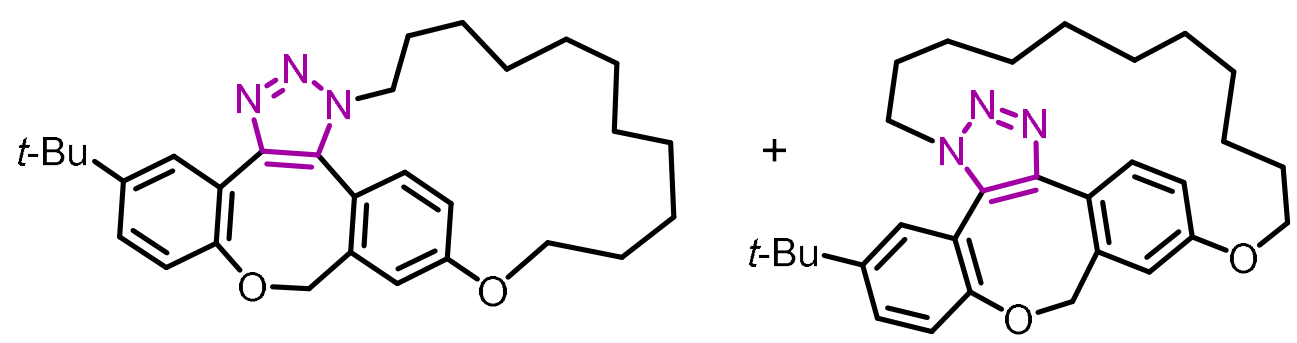

14a **14b**

Macrocycles 14a and **14b**. A solution of photo-ODIBO-C_{11}-N_3 (**12**, 0.025 g, 0.05 mmol) in CH_2Cl_2 (200 mL) was irradiated (14 × 350 nm lamps) for 5 min with stirring and incubated for 22 h at room temperature in the dark. The reaction mixture was concentrated in vacuo, purified by chromatography (acetone: hexanes 1:5) to afford two macrocyclic isomers, one major **14a** (17.5 mg, 74%) as a colorless oil and one minor **14b** (5.4 mg, 22%) as a white solid.

Major isomer **14a**

^1H NMR (CD_2Cl_2) δ 7.32 (m, 2H), 7.03 (m, 2H), 6.97 (m, 2H), 5.17 (d, 1H), 4.95 (d, 1H), 4.35 (m, 1H), 4.20 (m, 2H), 3.71 (m, 1H), 2.36 (m, 1H), 1.95 (m, 1H), 1.72 (m, 2H), 1.61 (m, 2H), 1.46 (m, 4H), 1.30 (m, 4H), 1.27 (s, 9H), 1.12 (m, 4H).

$^{13}C\{^1H\}$ NMR (CD_2Cl_2) δ 158.0, 153.7, 145.1, 140.9, 137.3, 135.2, 130.1, 128.7, 128.4, 122.9, 122.4, 121.9, 115.5, 113.8, 72.5, 69.6, 48.4, 34.5, 31.5, 29.8, 29.1, 28.8, 28.3, 27.2, 26.9, 26.1, 25.5, 24.5.

Minor isomer **14b**

^1H NMR δ 7.47 (m, 2H), 7.21 (m, 2H), 7.10 (d, 1H), 6.76 (d, 1H), 5.20 (d, 1H), 4.85 (d, 1H), 4.22 (m, 1H), 4.11 (m, 2H), 3.92 (m, 1H), 2.15 (m, 2H), 1.75 (m, 2H), 1.56 (m, 4H), 1.45 (m, 4H), 1.33 (m, 2H), 1.29 (s, 9H).

$^{13}C\{^1H\}$ NMR δ 156.7, 152.3, 146.1, 143.9, 138.8, 131.8, 130.5, 129.9, 127.4, 123.7, 120.0, 117.6, 116.5, 113.8, 69.9, 69.3, 50.1, 34.3, 31.5, 30.1, 29.3, 28.5, 27.3, 26.8, 25.9, 24.9, 24.4, 24.3.

HRMS (ESI/ Orbitrap), *m/z*: $[M+H]^+$ Calcd for $C_{30}H_{40}N_3O_2^+$ 474.3115; Found 474.3110

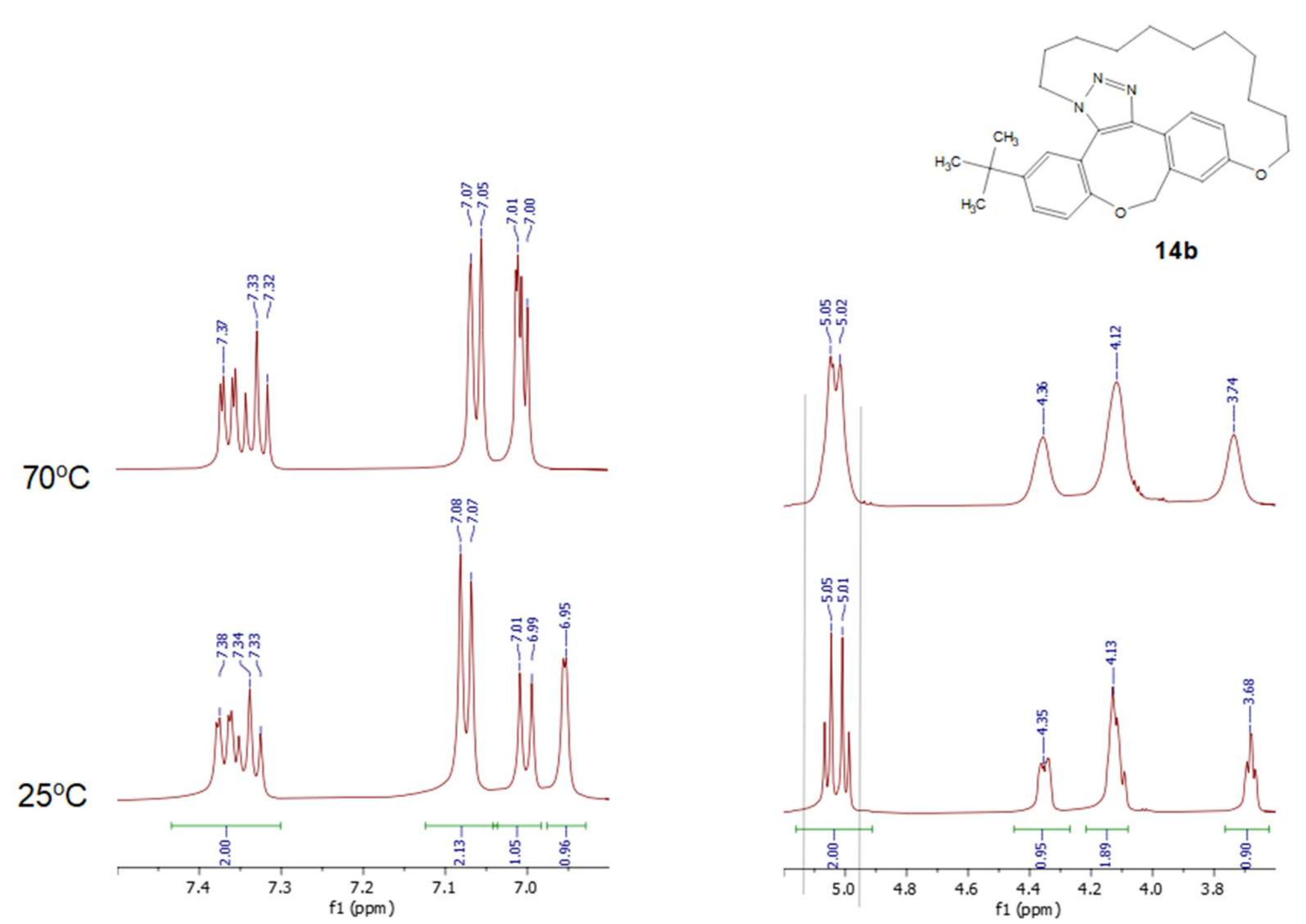

Figure S8. Variable temperature ^{1}H NMR spectra of the macrocycle **14b**.

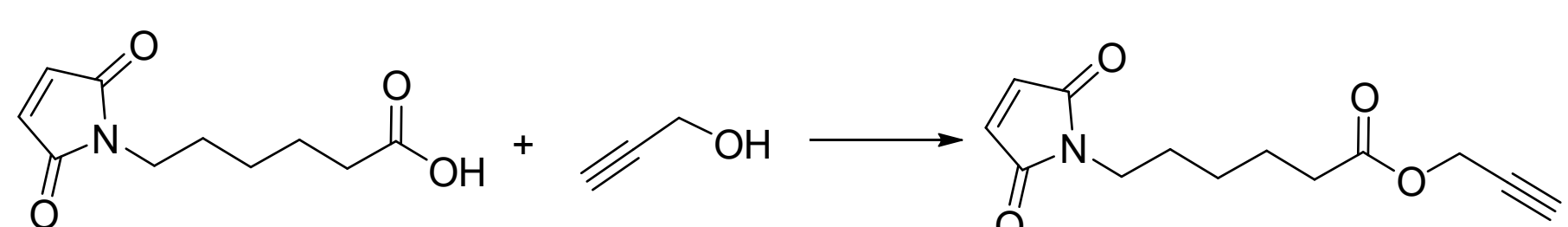

37

Prop-2-yn-1-yl 6-(2,5-dioxo-2,5-dihydro-1H-pyrrol-1-yl)hexanoate (**37**).[9] Propargyl alcohol (265 mg, 0.28 mL, 4.7 mmol) and 6-(2,5-dioxo-2,5-dihydro-1H-pyrrol-1-yl)hexanoate (400 mg, 3.3 mmol) were dissolved in toluene (4 mL). 3 drops of sulfuric acid was added and the reaction mixture was refluxed for overnight. The reaction mixture was diluted with ethyl acetate (100 mL) and washed with brine (2 × 50 mL) and dried over anhydrous sodium sulfate. Crude product was purified by silica gel chromatography to get 400 mg (68%) of **37** as a yellow oil.

^{1}H NMR δ 6.65 (s, 2H), 4.61 (s, 2H), 3.48-3.43 (m, 2H), 2.45 (s, 1H), 2.32 - 2.27 (m, 2H), 1.64 - 1.51 (m, 4H), 1.30 - 1.25 (m, 2H).

^{13}C{^{1}H} NMR δ 172.5, 170.8, 134.1, 77.7, 74.9, 51.8, 37.5, 33.7, 28.2, 26.1, 24.2.

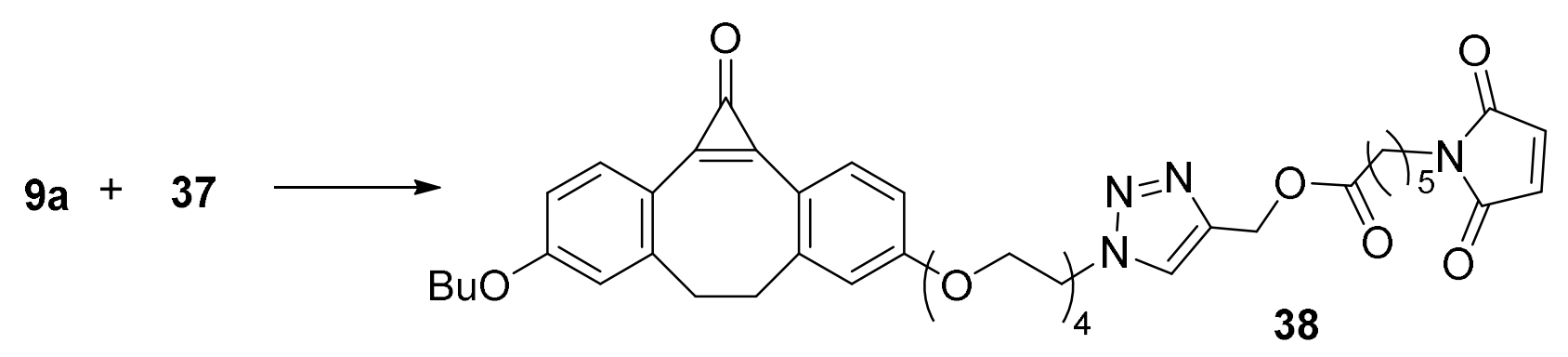

(1-(2-(2-(2-(2-((9-butoxy-1-oxo-6,7-dihydro-1H-dibenzo[a,e]cyclopropa[c][8]annulen-4-yl)oxy)ethoxy)ethoxy)ethoxy)ethyl)-1H-1,2,3-triazol-4-yl)methyl 6-(2,5-dioxo-2,5-dihydro-1H-pyrrol-1-yl)hexanoate (**38**). Photo-DIBO-EG$_4$-N$_3$ (**9a**, 261 mg, 1 Eq, 0.50 mmol), prop-2-yn-1-yl 6-(2,5-dioxo-2,5-dihydro-1H-pyrrol-1-yl)hexanoate (**37**, 137 mg, 1.1 Eq, 0.55 mmol), copper (I) iodide (28.6 mg, 0.3 Eq, 0.15 mmol), and sodium ascorbate (52.9 mg, 0.6 Eq, 0.3 mmol) were dissolved in 5 mL of THF : H_2O mixture (4:1) at r.t. under stirring. After completion of the reaction (~ 26 h, TLC control), the reaction mixture was diluted with DCM (50 mL), washed with brine (2 × 25 mL), and concentrated in vacuum. Crude product was purified by chromatography (10-30% acetone in ethyl acetate) to produce 310 mg (80%) of **38** as a colorless oil.

^{1}H NMR δ 7.91 - 7.89 (m, 2H), 7.80 (s, 1H), 6.90 - 6.87 (m, 4H), 6.67 (s, 2H), 5.20 (s, 2H), 4.53 (t, J = 5 Hz, 2H), 4.19 (t, J = 4 Hz, 2H), 4.03 (t, J = 6 Hz, 2H), 3.87 (t, J = 5 Hz, 4H), 3.73 - 3.70 (m, 2H), 3.65 - 3.61 (m, 6H), 3.47 (t, J = 7 Hz, 2H), 3.33 - 3.31 (m, 2H), 2.62 - 2.59 (m, 2H), 2.30 (t, J = 7 Hz, 2H), 1.82 - 1.75 (m, 2H), 1.66 - 1.45 (m, 6H), 1.30 - 1.21 (m, 2H), 0.98 (t, J = 7.4 Hz, 3H).
^{13}C{^{1}H} NMR δ 173.2, 161.5, 142.4, 141.9, 135.6, 134.0, 124.9, 116.5, 116.3, 116.2, 116.1, 112.3, 70.8, 70.6, 70.5, 69.5, 69.3, 68.0, 67.6, 57.5, 50.2, 37.5, 37.1, 37.1, 33.8, 31.1, 28.2, 26.1, 24.3, 19.2, 13.8.
HRMS (ESI/ Orbitrap), *m/z*: $[M+H]^+$ Calcd for $C_{42}H_{51}N_4O_{10}^+$ 771.3600, found 771.3606.

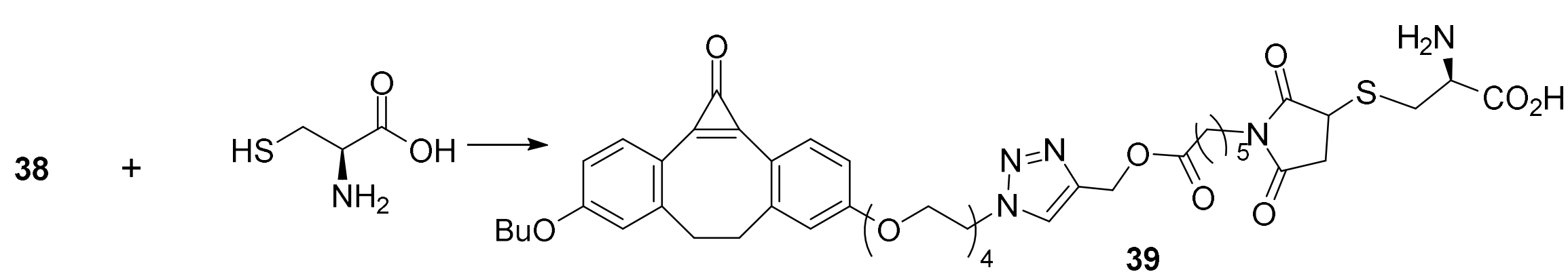

S-(1-(6-((1-(2-(2-(2-(2-((9-butoxy-1-oxo-6,7-dihydro-1H-dibenzo[a,e]cyclopropa[c][8] annulen-4-yl)oxy)ethoxy)ethoxy)ethoxy)ethyl)-1H-1,2,3-triazol-4-yl)methoxy)-6-oxohexyl)-2,5-dioxopyrrolidin-3-yl)cysteine (**39**) - Compound **38** (200 mg, 1 Eq, 0.26 mmol) and cysteine (63 mg, 2 Eq, 0.52 mmol) were dissolved in DMF (5 mL) and triethylamine (39 mg, 1.5 Eq, 0.39 mmol) was added dropwise. The reaction was stirred for 4 hours at r.t., diluted with DCM (50 mL) and washed with brine (2 × 30 mL). The organic layer was concentrated in

vacuum and purified by silica gel column chromatography (5-20% Methanol in DCM) to give 170 mg (75%) of 40 as a yellowish oil.

^{1}H NMR (DMSO) δ 8.13 (s, 1H), 7.78 (d, J = 8.4 Hz, 2H), 7.18 – 6.98 (m, 4H), 5.10 (s, 2H), 4.52 (t, J = 5.2 Hz, 2H), 4.27 – 4.05 (m, 5H), 3.79 (m, 4H), 3.64 – 3.27 (m, 18H), 3.17 (d, J = 2.5 Hz, 3H), 2.46 (d, J = 10.8 Hz, 2H), 2.28 (t, J = 7.4 Hz, 2H), 1.77 – 1.65 (m, 2H), 1.46 (m, 6H), 1.19 (t, J = 7.2 Hz, 3H), 0.94 (t, J = 7.4 Hz, 3H).

^{13}C{^{1}H} NMR (DMSO) δ 177.6, 177.4, 175.6, 175.5, 173.0, 162.8, 162.0, 161.8, 152.5, 148.5, 142.5, 142.3, 142.2, 135.4, 135.4, 125.6, 116.7, 116.4, 116.2, 113.3, 70.4, 70.2, 70.1, 70.0, 69.2, 69.1, 68.1, 68.0, 57.5, 49.8, 45.7, 40.6, 40.4, 40.2, 40.0, 39.7, 39.5, 39.3, 36.7, 36.3, 33.6, 31.1, 27.1, 25.9, 24.3, 19.2, 14.1.

HRMS (ESI/Orbitrap), *m/z*: $[M-H]^-$ Calcd for $C_{45}H_{56}N_5O_{12}S^-$ 890.3652, found 890.3632.

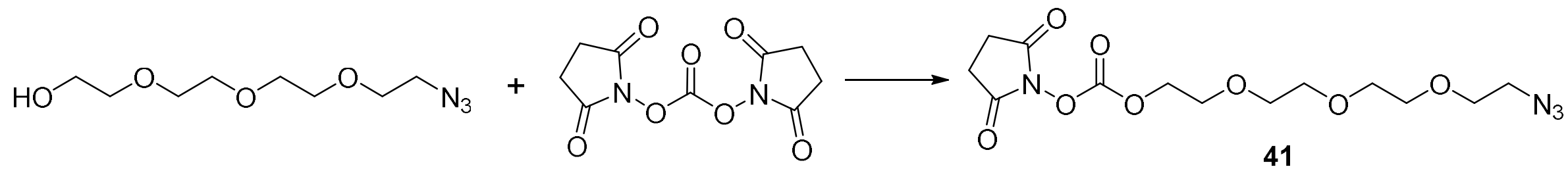

41

2-(2-(2-(2-azidoethoxy)ethoxy)ethoxy)ethyl (2,5-dioxopyrrolidin-1-yl) carbonate (**41**)[10] - 2-(2-[2-[2-azidoethoxy)ethoxy]ethoxy]ethanol (1 g, 1 Eq, 4.5 mmol), bis(2,5-dioxopyrrolidin-1-yl) carbonate (3 g, 2.5 Eq, 11.4 mmol), and potassium carbonate (630 mg, 1 Eq, 4.6 mmol) were dissolved in acetonitrile (20 mL) and stirred for overnight at r.t. The solvent was evaporated in vacuum, the reaction mixture was diluted with ethyl acetate (60 mL) and washed with brine (2 × 30 mL). Aqueous layer was extracted with DCM (50 mL). Combined organic layer were dried over anhydrous $CaCl_2$ and the solvents removed in vacuum at 40 ^{0}C to give 1.4 g (85%) of **41** as a colorless oil.

^{1}H NMR δ 4.50 - 4.41 (m, 2H), 3.82 - 3.74 (m, 2H), 3.69 - 3.67 (m, 10H), 3.39 (t, J = 5 Hz, 2H), 2.84 (s, 4H)

^{13}C{^{1}H} NMR δ 168.6, 168.6, 151.7, 70.9, 70.7, 70.7, 70.7, 70.3, 70.0, 68.3, 50.7, 25.5.

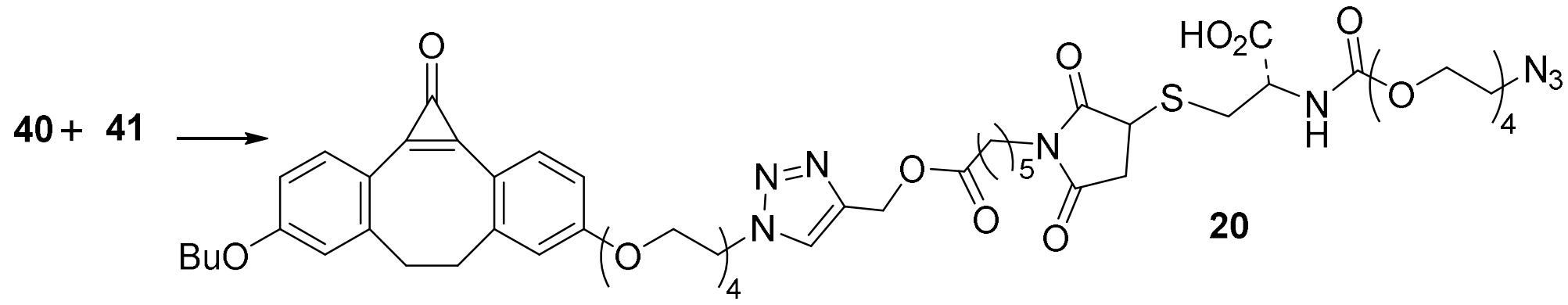

20

N-(13-azido-2,5,8,11-tetraoxatridecanoyl)-S-(1-(6-((1-(2-(2-(2-(2-((9-butoxy-1-oxo-6,7-dihydro-1H-dibenzo[a,e]cyclopropa[c][8]annulen-4-yl)oxy)ethoxy)ethoxy)ethoxy)ethyl)-1H-1,2,3-triazol-4-yl)methoxy)-6-oxohexyl)-2,5-dioxopyrrolidin-3-yl)cysteine (**20**). Compound **40** (80 mg, 1 Eq, 90 μmol) and triethylamine (11 mg, 15 μL, 1.2 Eq, 0.11 mmol) were stirred in DCM (3 mL) for 10 minutes and then a solution of 2-(2-(2-(2-azidoethoxy)ethoxy)ethoxy)ethyl (2,5-dioxopyrrolidin-1-yl) carbonate (**41**, 65 mg, 2 Eq, 0.18 mmol) in 1 mL DCM was added. The reaction mixture was stirred overnight at r.t., solvents were removed in vacuum, and the crude product was purified by chromatography (2-10% methanol in DCM) to give 58 mg (75%) of **20** as a yellowish oil.

^{1}H NMR (600 MHz) δ 7.91 (d, J = 6.0 Hz, 2H), 7.80 (s, 1H), 6.94 – 6.81 (m, 4H), 5.17 (s, 2H), 4.52 (t, J = 5.1 Hz, 2H), 4.18 (dt, J = 6.9, 3.4 Hz, 3H), 4.02 (td, J = 6.6, 2.2 Hz, 3H), 3.85 (dd, J = 6.8, 3.3 Hz, 4H), 3.71 – 3.57 (m, 15H), 3.47 – 3.28 (m, 5H), 3.08 (d, J = 7.4 Hz, 2H), 2.59 (d, J = 11.1 Hz, 2H), 2.27 (q, J = 5.5 Hz, 2H), 1.82 – 1.71 (m, 2H), 1.58 – 1.39 (m, 12H), 0.97 (td, J = 7.3, 1.4 Hz, 4H).
^{13}C{^{1}H} NMR (151 MHz) δ 174.8, 173.3, 173.2, 162.3, 161.7, 154.0, 148.0, 142.6, 142.0, 141.6, 135.9, 135.9, 125.0, 116.4, 116.4, 116.3, 115.9, 112.5, 112.4, 112.4, 70.8, 70.6, 70.6, 70.54, 70.52, 70.0, 69.52, 69.46, 69.3, 68.1, 67.7, 57.4, 53.9, 53.5, 50.7, 50.3, 42.0, 37.2, 37.1, 33.8, 33.8, 31.1, 29.7, 29.7, 24.2, 19.2, 13.8, 12.0.
HRMS (ESI/ Orbitrap), *m/z*: [M-H]$^-$ Calcd for $C_{54}H_{71}N_8O_{17}S^-$ 1135.4663, found 1135.4677.

Macrocycle **22**: HRMS (ESI/ Orbitrap), *m/z*: [M-H]$^-$ Calcd for $C_{53}H_{71}N_8O_{16}S^-$ 1107.4714, found 1107.4697.

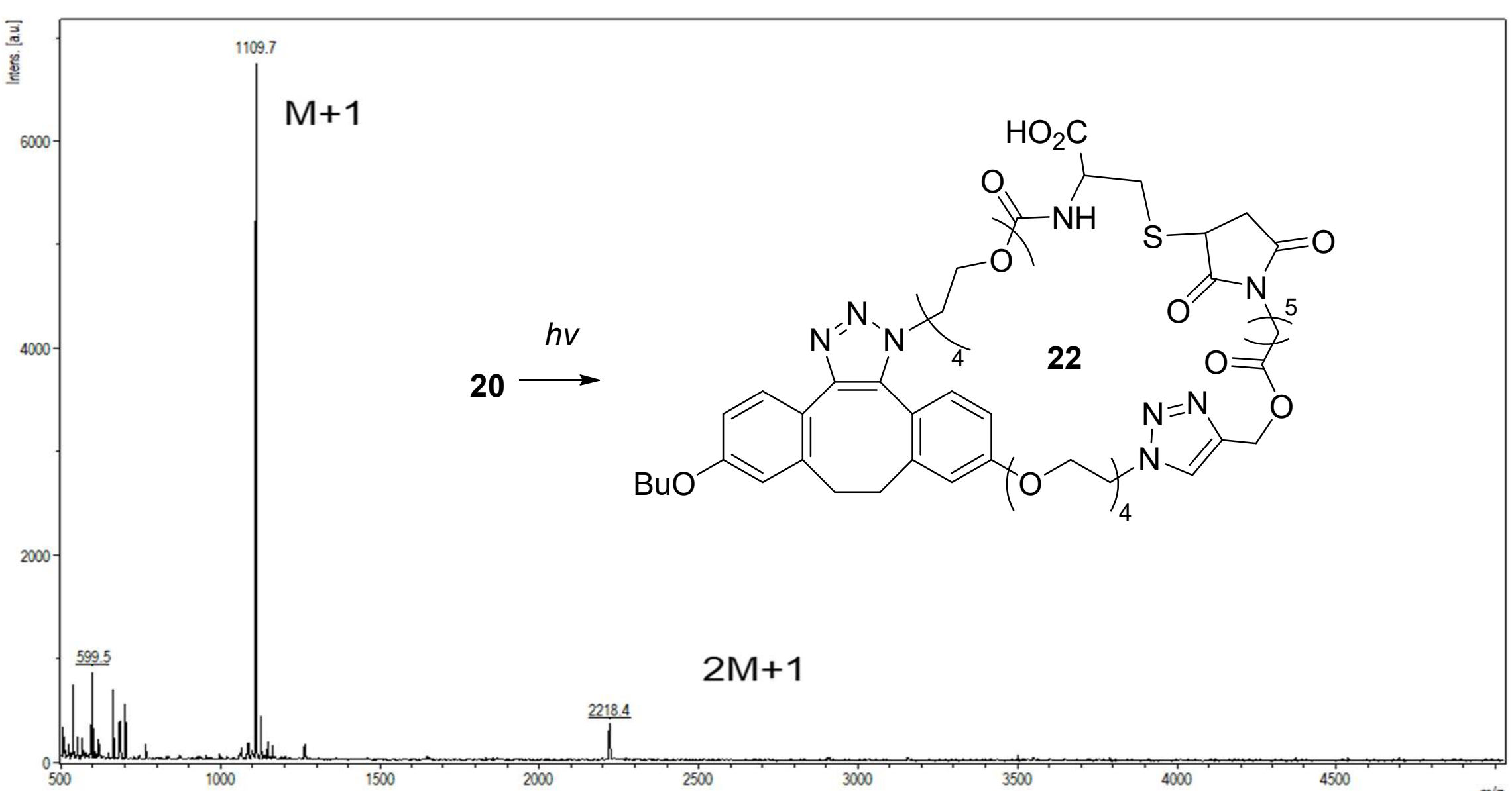

Figure S9. Mass spectrum of the photolysate of **20** in DCM treated with MeOH solution of NaN_3.

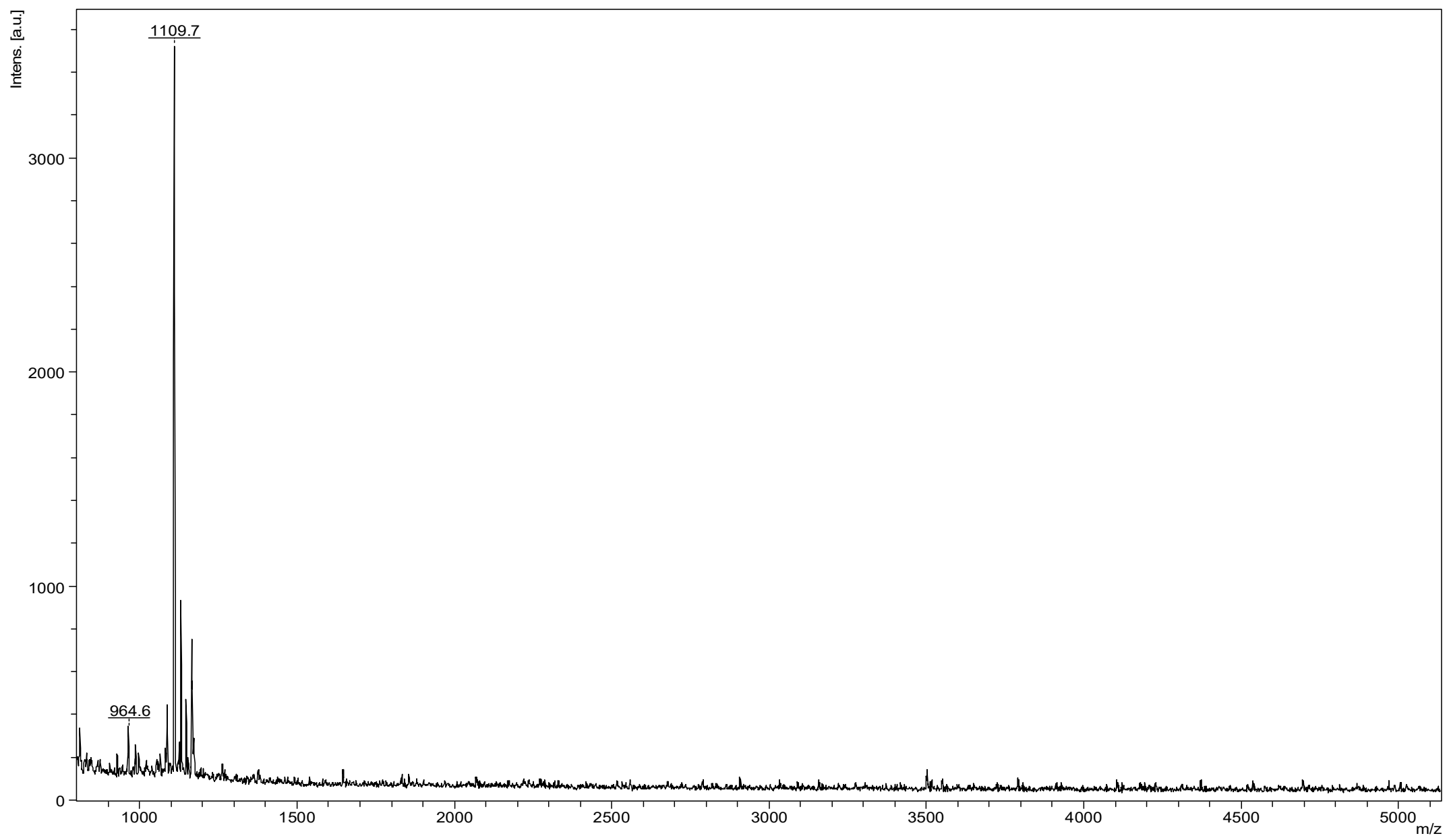

Figure S10. Mass spectrum of the photolysate of neat **20** treated in the dark with NaN_3.

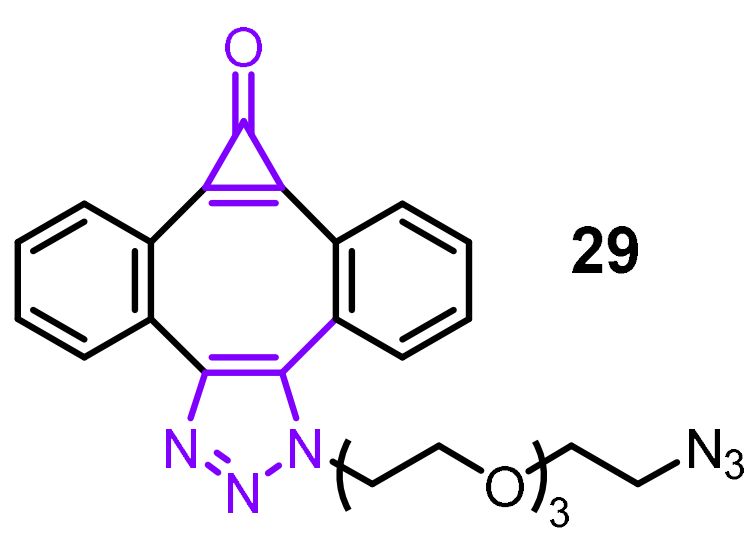

29

1-(2-(2-(2-(2-azidoethoxy)ethoxy)ethoxy)ethyl)dibenzo[3,4:7,8]cyclopropa[5,6]cycloocta [1,2-*d*][1,2,3]triazol-8(*1H*)-one (MC-DIBOT-EG$_4$-N$_3$, **29**). A solution of bis-azido-EG$_4$ (**25**, 30 mg, 0.13 mmol) in 5 mL of DCM was added to a solution of MC-DIBOD (**28**, 30 mg, 0.13 mmol) in 5 mL of DCM and stirred at r.t. for 16 h. The solvent was removed under vacuum and the crude product was purified by chromatography (50% acetone/hexanes) to give 11 mg (18%) of the product as yellowish oil.

^{1}H NMR δ 1H 7.87 (ddd, J = 7.8, 3.7, 1.3 Hz, 2H), 7.69 – 7.65 (m, 2H), 7.63 – 7.49 (m, 2H), 7.45 (tt, J = 7.6, 1.5 Hz, 2H), 4.45 – 4.37 (m, 2H), 4.31 – 4.22 (m, 2H), 3.99 – 3.92 (m, 2H), 3.86 – 3.79 (m, 2H), 3.52 – 3.43 (m, 8H).

^{13}C{^{1}H} NMR δ 157.5, 154.7, 152.0, 142.8, 133.9, 133.9, 133.2,132.5, 132.4, 132.3, 131.6, 131.4, 130.6, 129.5, 128.7, 127.1, 124.6, 70.7, 70.6, 70.4, 70.3, 69.6, 61.7, 50.8, 49.0.

MS (ESI), *m/z*: [M+Na]$^+$ Calcd for $C_{25}H_{24}N_6O_4Na^+$ 495.1757, found 494.9.

Macrocycles 26a,b (1:3 mixture).

^{1}H NMR δ 1H 7.71 (dd, J = 7.8, 1.2 Hz, 2H), 7.67 – 7.64 (m, 0.6 H), 7.55 - 7.51 (m, 2.6 H), 7.49 – 7.44 (m, 2.6 H), 7.29-7.27 (m, 0.6 H), 7.22 (d, J = 7.2 Hz, 2H) 4.74 - 4.68 (m, 2H), 4.61 – 4.55 (m, 0.6 H), 4.48 – 4.38 (m, 2.6 H), 4.05 – 3.99 (m, 0.6 H), 3.82 – 3.77 (m, 0.6 H), 3.73 – 3.67 (m, 2 H), 3.63 – 3.56 (m, 0.6 H), 3.50 – 3.45 (m, 1.2 H), 3.29 – 3.25 (m, 2H), 3.18 – 3.07 (m, 6H), 2.98 – 2.93 (m, 2H).

^{13}C{^{1}H} NMR δ 146.0, 145.4, 134.8, 134.0, 132.7, 131.4, 131.1, 130.82, 130.56, 130.07, 130.03, 129.3, 129.09, 129.04, 128.4, 127.2, 71.7, 70.21, 70.20, 70.1, 69.0, 68.4, 49.4, 48.6.

157.5, 154.7, 152.0, 142.8, 133.9, 133.9, 133.2,132.5, 132.4, 132.3, 131.6, 131.4, 130.6, 129.5, 128.7, 127.1, 124.6, 70.7, 70.6, 70.4, 70.3, 69.6, 61.7, 50.8, 49.0.

HRMS (MALDI-TOF), *m/z*: [M+Na]$^+$ Calcd. for $C_{24}H_{24}N_6NaO_3^+$ 467.180, found 467.211.

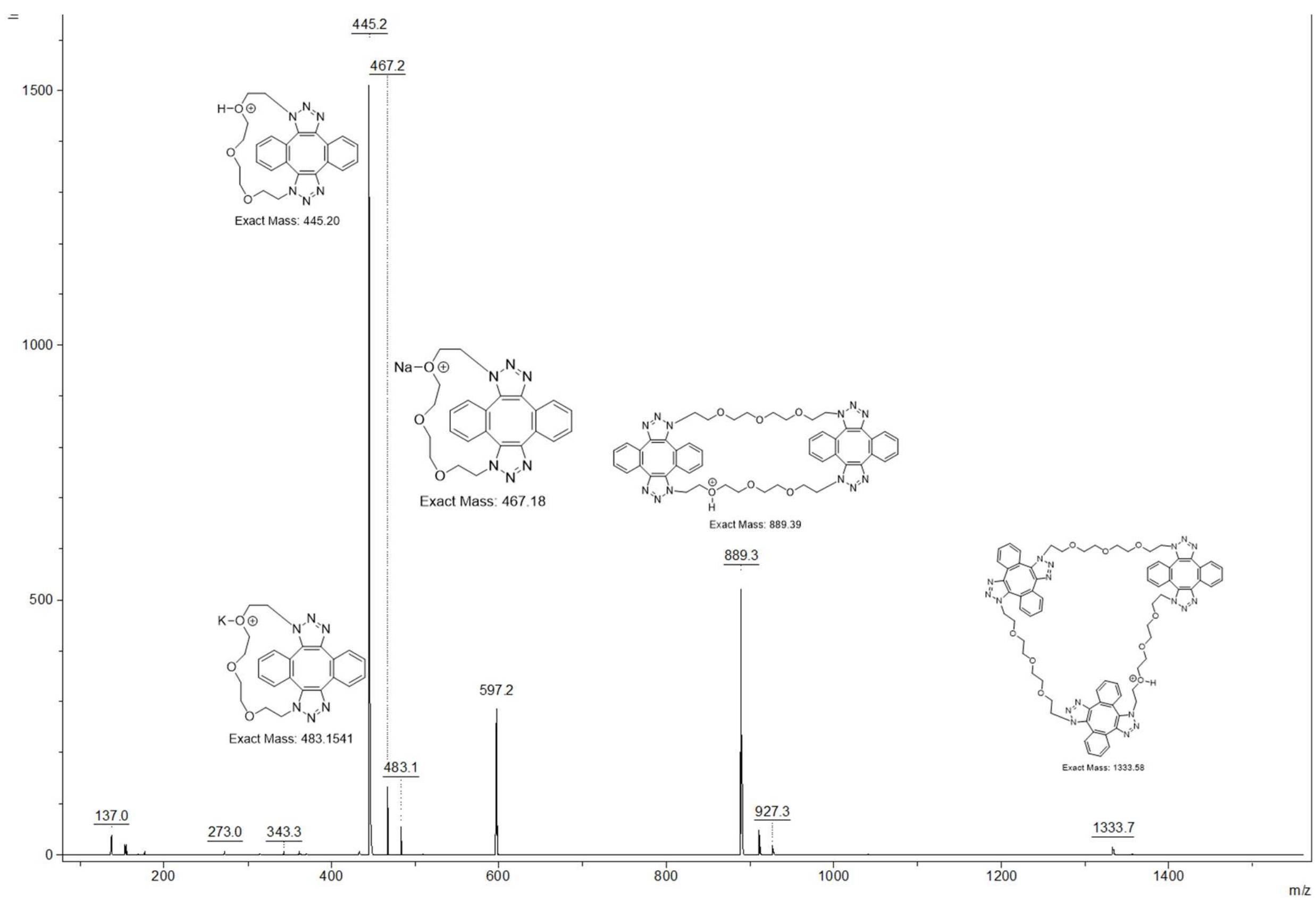

Figure S11. Mass spectrum of the photolysate of photo-DIBOD (**23**) in the presence of 1 eq. of bis-TEG azide (**25**).

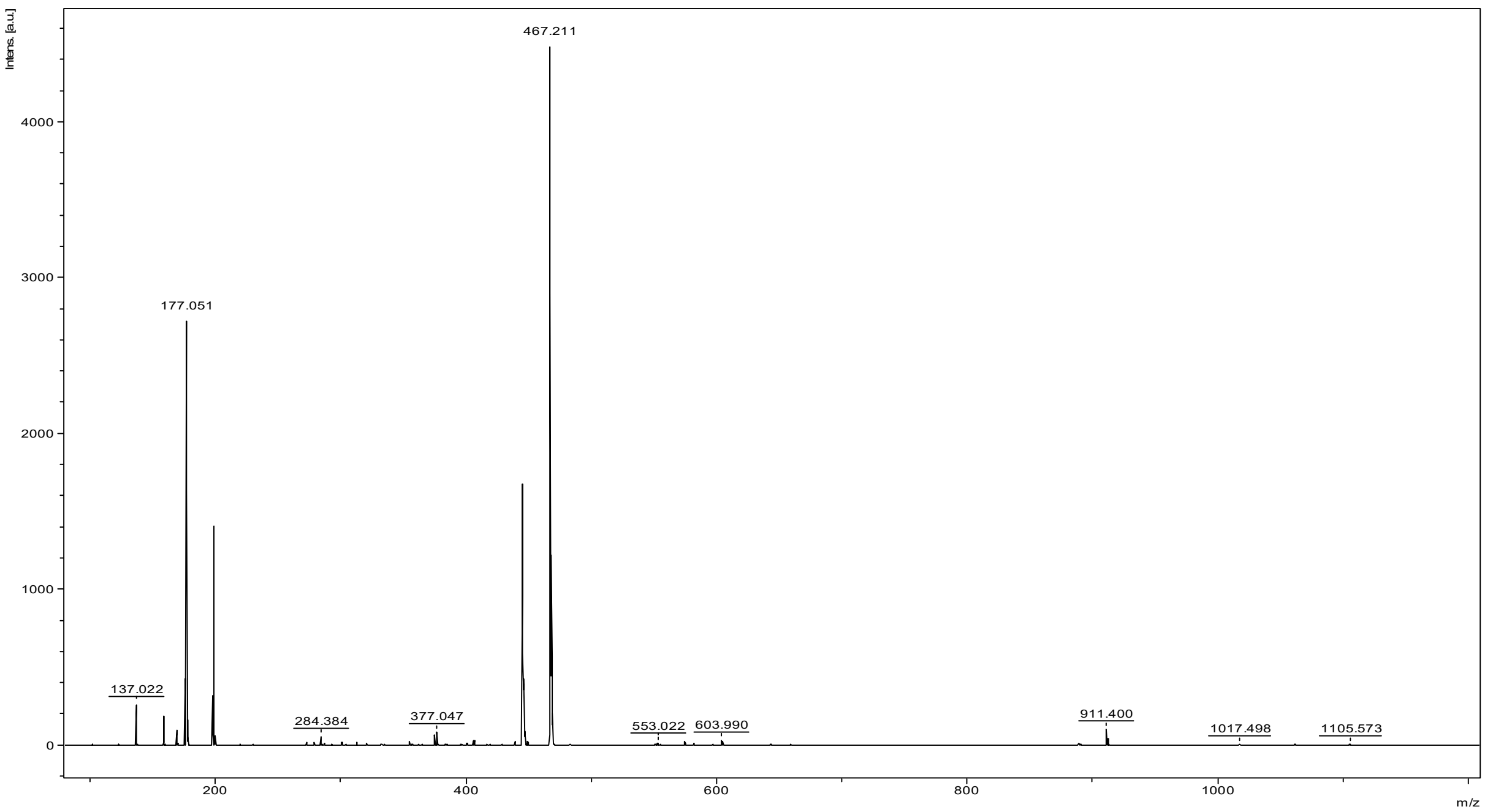

Figure S12. Mass spectrum of the reaction of photogenerated of DIBOD (**24**) with subsequent addition of 1 eq. of bis-TEG azide (**25**).

Macrocycle 27: HRMS (ESI), *m/z*: $[M+H]^+$ Calcd for $C_{48}H_{49}N_{12}O_6^+$: 889.3893, found 889.3890

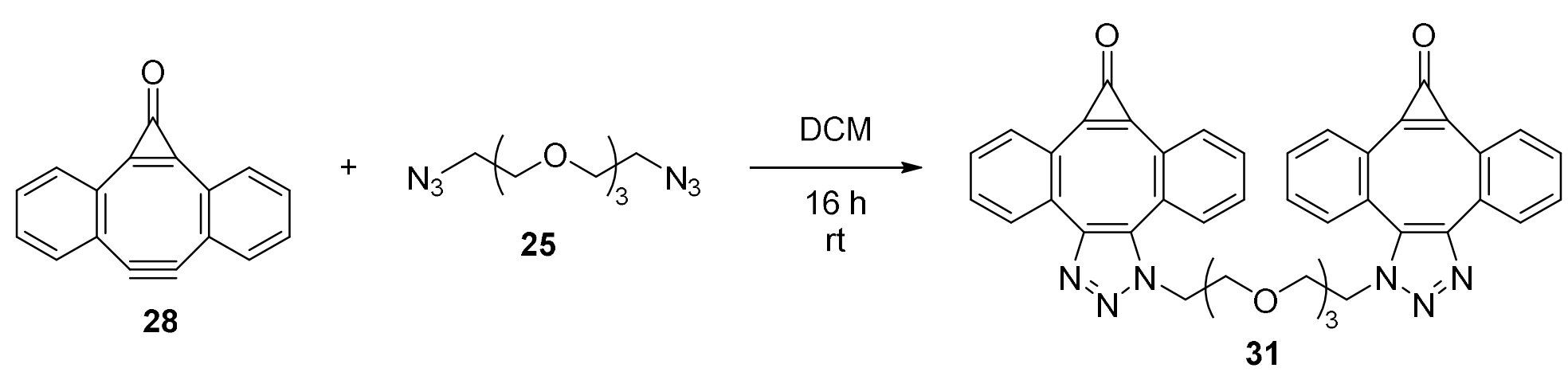

1,1'-(((oxybis(ethane-2,1-diyl))bis(oxy))bis(ethane-2,1-diyl))bis(dibenzo[3,4:7,8] cyclopropa[5,6]cycloocta[1,2-d][1,2,3]triazol-8(1H)-one (**31**). Bis-azido-EG$_4$ (**25**, 15 mg, 0.063 mmol) was dissolved in DCM (5 mL) and MC-DIBOD (**28**, 30 mg, 0.131 mmol) was added to the solution. The mixture was stirred for 16 h at r.t. The solvent was removed under vacuum and the crude product was purified by chromatography (50% acetone/hexanes) to give 8 mg (19%) of **31** as a light-yellow semi-solid.

^{1}H NMR δ 7.87 (m, 2H), 7.69 – 7.65 (m, 2H), 7.63 – 7.43 (m, 12H), 4.45 – 4.37 (m, 2H), 4.31 – 4.22 (m, 2H), 3.99 – 3.92 (m, 2H), 3.86 – 3.79 (m, 2H), 3.52 – 3.43 (m, 8H).

^{13}C{^{1}H} NMR δ 157.5, 154.7, 152.0, 142.8, 133.93, 133.87, 133.2, 132.53, 132.38, 132.28, 131.6, 131.4, k130.6, 129.5, 128.7, 127.1, 124.6, 70.74, 70.58, 69.60, 48.96.

MS (ESI), *m/z*: $[M+H]^+$ Calcd for $C_{42}H_{33}N_6O_5^+$ 701.2507, found 701.2.

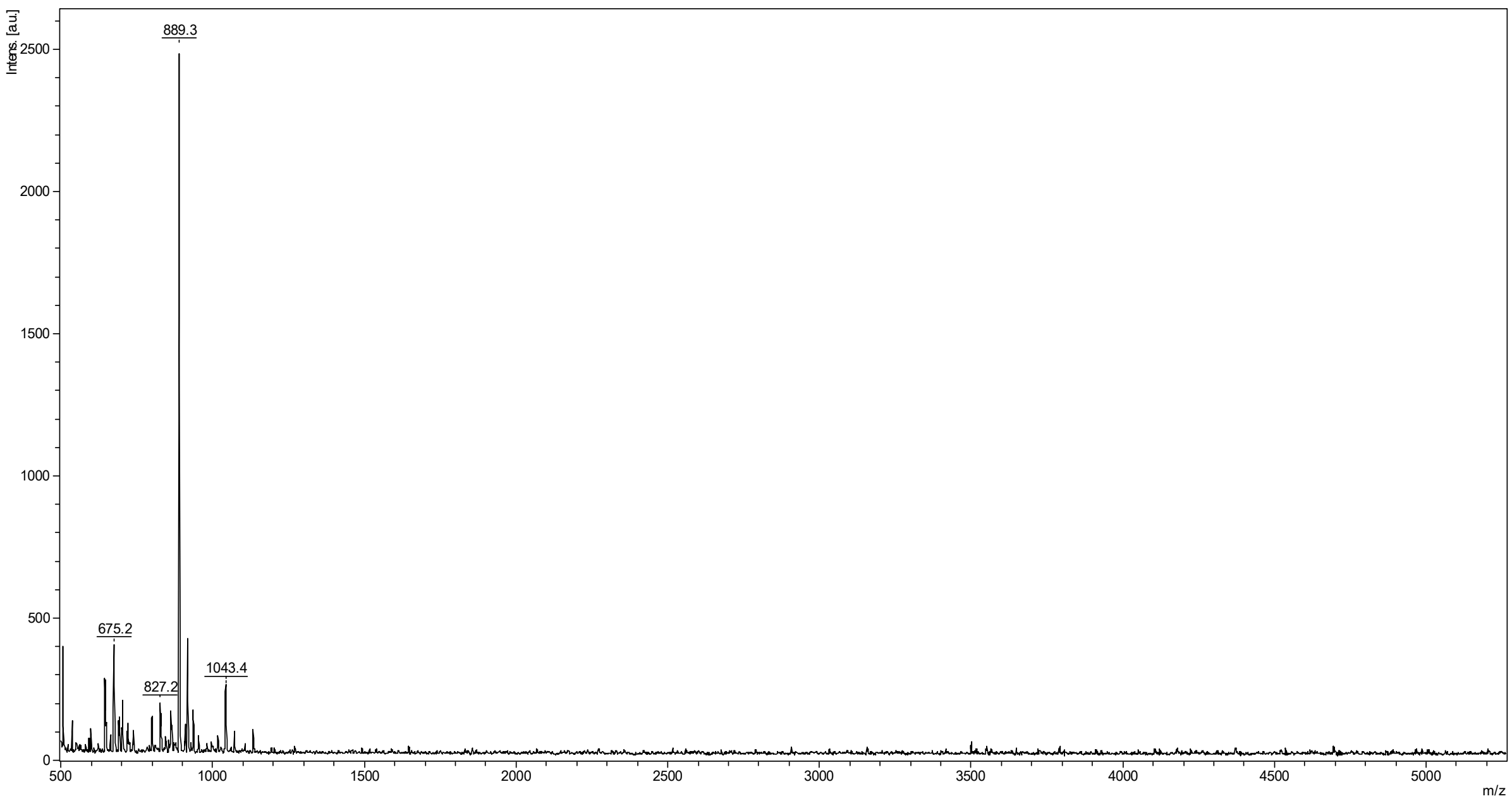

Figure S12. Mass spectrum of the photolysate of **31** in the presence of 1 eq. of bis-TEG azide (**25**) with subsequent addition of NaN_3.

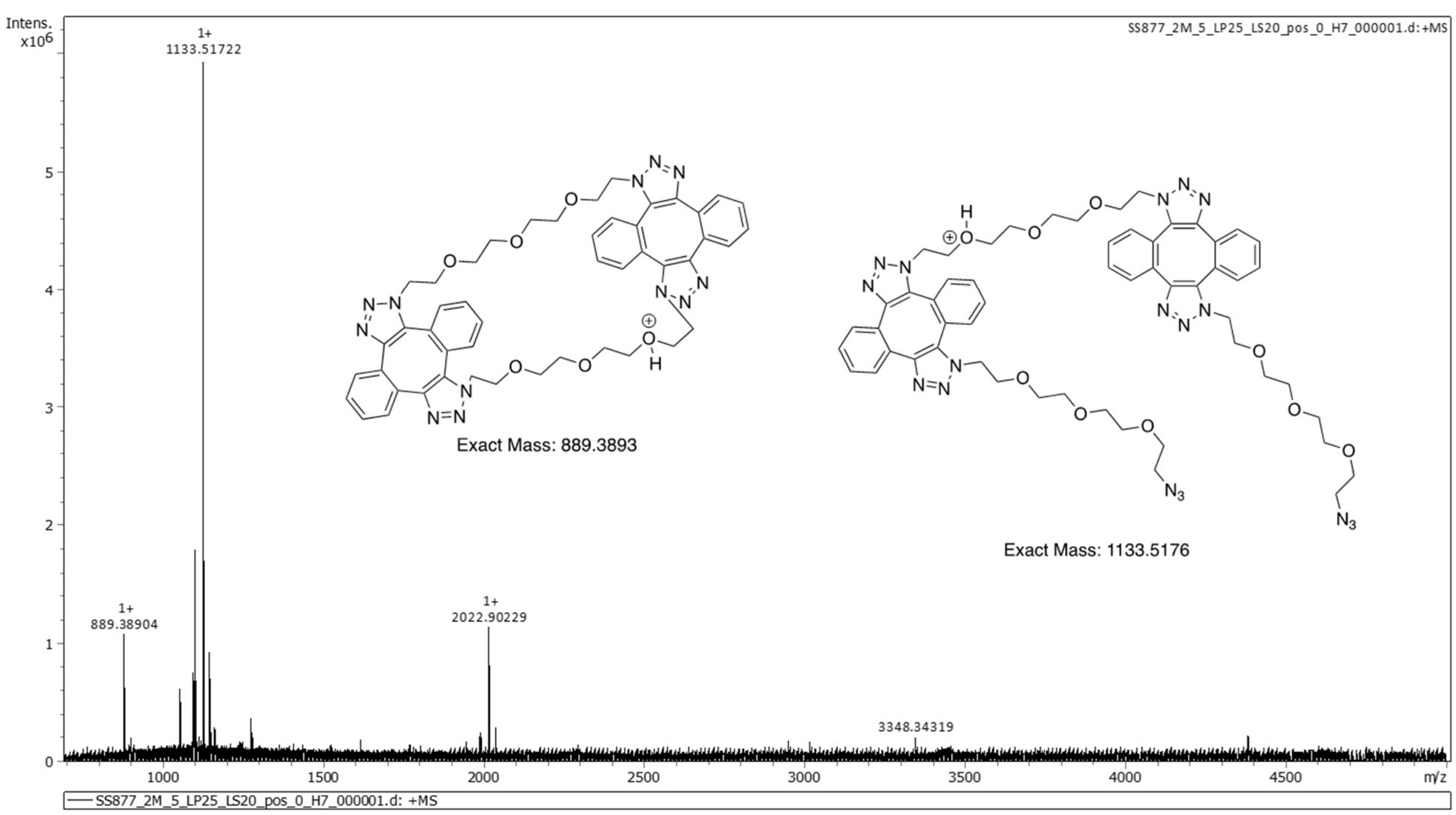

Figure S13. Mass spectrum of the photolysate of **31** in the presence of 100 eq. of bis-TEG azide (**25**) with subsequent addition of NaN_3.

Theoretical Procedures. Density functional theory calculations were carried out using Gaussian 16 Revision C01.[11] Geometries were pre-optimized using a B3LYP hybrid functional and 6-31+G(d) basis set and then re-optimized using extended basis set at B3LYP/6-311+G(d,p) level. Comparison of the transition state energies of the cyclization versus dimerization reaction were conducted using B3LYP/6-31+G(d)//B3LYP/6-311+G(d,p) method. Zero-point vibrational energy (ZPVE) corrections, required to correct the raw relative energies to 0 ^{0}K, were obtained from B3LYP/6-311+G(d,p) frequency calculations. Analytical second derivatives were computed to confirm each stationary point to be a minimum by yielding zero imaginary vibrational frequencies for the intermediates and one imaginary vibrational frequency for each transition state. The DFT frequency analyses are known to overestimate the magnitude of the vibrational frequencies.[12] We have compared experimental vibrational frequencies for triple bond and azide moieties in **7**, **10**, **13** with values calculated at B3LYP/6-311+G(d,p) level to estimate the average scaling parameter as 0.96 (e.g., ODIBO $\nu_{C\equiv C}$, experimental: 2151.0 cm^{-1} and DFT: 2249 cm^{-1}.). All ZPVE were scaled by this factor. Optimized geometries obtained from B3LYP/6-31++G(d,p) calculations are listed below in Gaussian 16 output format. The effect on the solvent (MeOH) on the activation barrier was evaluated using SMD solvation model.[13]

ODIBO-EG$_4$-N$_3$ (**7a**)

C,9.692934,-0.206999,0.232191
C,9.257953,-1.531773,0.402057
C,7.908253,-1.870799,0.405097
C,6.935927,-0.888415,0.248301
C,7.344936,0.459094,0.081191
C,8.712198,0.772117,0.069963
C,11.198439,0.114039,0.233053
C,11.815269,-0.313665,1.585306
C,11.890896,-0.659456,-0.913294
C,11.476243,1.615224,0.033788
O,5.611519,-1.238875,0.356261
C,4.71185,-1.03196,-0.773452
C,3.524927,-0.138779,-0.474544
C,3.714511,1.250059,-0.213313
C,5.079128,1.622665,-0.104779
C,6.264936,1.368573,-0.061806
C,2.237024,-0.646081,-0.520688
C,1.125171,0.18969,-0.335105
C,1.309575,1.553298,-0.099171
C,2.602595,2.072303,-0.036481
O,-0.089867,-0.425783,-0.405854
C,-1.26976,0.350927,-0.217706
C,-2.444083,-0.6057,-0.339279

N,-14.307558,-0.483367,0.183914
N,-15.359495,0.132646,0.332437
N,-16.39143,0.589638,0.453208
O,-3.622633,0.157093,-0.158454
C,-4.811055,-0.61277,-0.241657
C,-5.983875,0.329664,-0.029985
O,-7.170483,-0.440492,-0.112616
C,-8.350624,0.32233,0.068807
C,-9.531079,-0.627623,-0.043237
O,-10.713367,0.13567,0.13692
C,-11.897725,-0.63386,0.05659
C,-13.066521,0.315423,0.26692
H,9.984117,-2.325305,0.535304
H,7.592237,-2.897973,0.545172
H,8.982331,1.810802,-0.061578
H,12.88719,-0.093026,1.600397
H,11.693273,-1.383888,1.767739
H,11.345796,0.222211,2.414798
H,12.963777,-0.443258,-0.923504
H,11.476773,-0.371759,-1.883626
H,11.770348,-1.739964,-0.80577
H,12.555264,1.791632,0.041229
H,11.038577,2.220293,0.83249
H,11.090184,1.977966,-0.922809
H,4.354133,-2.024064,-1.058582
H,5.283226,-0.617373,-1.607619
H,2.061834,-1.699683,-0.707437
H,0.465997,2.214721,0.043595
H,2.747209,3.12777,0.160311
H,-1.343413,1.134077,-0.980274
H,-1.269631,0.821047,0.771756
H,-2.365523,-1.393241,0.422754
H,-2.435121,-1.085667,-1.327602
H,-4.819804,-1.398366,0.526264
H,-4.892979,-1.096263,-1.224777
H,-5.974704,1.115476,-0.798033
H,-5.900874,0.813876,0.952915
H,-8.429312,1.107235,-0.696199
H,-8.354023,0.807198,1.054896
H,-9.454105,-1.41143,0.722634
H,-9.528189,-1.112718,-1.028901
H,-11.908525,-1.418096,0.826189
H,-11.982585,-1.121338,-0.92453
H,-12.97988,0.7969,1.246966
H,-13.053824,1.09372,-0.503737

TS_1(7a)
C,-3.3446688047,-0.2977612266,0.0853681662
C,-3.3038871398,-1.7086204537,-0.0181188902
O,-2.1265084353,-2.4148942196,0.0169312271
C,-1.1474796102,-2.235343189,-1.0441185266

C,0.190327539,-1.807999406,-0.4945273681
C,0.3269297679,-0.5208420394,0.0902184688
C,-0.8502276442,0.2867422897,0.1336444668
C,-2.0873651097,0.3897523676,0.127609379
C,-4.5959959635,0.3398051145,0.1387557612
C,-5.7974745723,-0.3676739689,0.1073262218
C,-5.7191476999,-1.7660214795,0.0101524619
C,-4.4969131686,-2.4248225916,-0.0573221557
C,1.2857741615,-2.6575808065,-0.5441193107
C,2.5249712115,-2.2699433962,-0.0141679638
C,2.6504407164,-1.0193810945,0.5954274696
C,1.5586691919,-0.1559440404,0.6362961863
O,3.5348909495,-3.1830754377,-0.1356481817
C,5.6534689501,-1.962550505,-0.3747685685
C,4.8000647202,-2.9086604441,0.4710650672
N,-2.415958781,2.6139129357,0.1715594258
N,-1.3013678679,2.8932412017,0.028892651
N,-0.1394645555,2.4189463074,0.0192042617
C,0.7819723861,2.9248850841,-1.0156937453
C,1.8415053833,3.9014219506,-0.4910443773
O,2.864413349,3.3031461597,0.2882694517
C,3.8240519072,2.5692227846,-0.4604945458
C,4.9859920932,2.2383410305,0.4652539007
O,5.7322443371,1.1984823522,-0.1502747217
C,6.9428472409,0.8753599614,0.5210065761
C,7.488495114,-0.4410335839,-0.0098277489
O,6.7532793027,-1.5707487067,0.4434067401
C,-7.1715450134,0.3228323113,0.1788014916
C,-7.0519648952,1.8545237811,0.2780533824
C,-7.9853773158,-0.0147229743,-1.0922687032
C,-7.9367772716,-0.1816231299,1.4246098176
H,-1.0556887727,-3.19886964,-1.5515670465
H,-1.5245558842,-1.4996737397,-1.7588589944
H,-4.5916374893,1.4164971127,0.2149280799
H,-6.6250567068,-2.3608816621,-0.0135171999
H,-4.4483832903,-3.505528445,-0.123246072
H,1.2008367064,-3.6441802275,-0.9858303003
H,3.5866740501,-0.6993260258,1.0298001476
H,1.6584036706,0.8148776412,1.1057952259
H,5.0696952696,-1.0924403228,-0.6811633262
H,6.0125820193,-2.4753462246,-1.2773558548
H,5.2855408785,-3.8794294263,0.5723591007
H,4.6683022565,-2.490359978,1.4729592511
H,0.2234095088,3.4059989645,-1.8246930701
H,1.2652200096,2.0403101499,-1.4371609389
H,1.370073397,4.648052989,0.150873708
H,2.2868116194,4.4206999792,-1.3534885785
H,4.1732980986,3.1606343363,-1.3183337702
H,3.4056916123,1.6302049374,-0.8407809277
H,4.5956507535,1.9119529342,1.4388575683
H,5.6133331684,3.1242472469,0.6328042971

H,6.7669023105,0.7797288395,1.6013804299
H,7.6874468156,1.6714955847,0.3638198501
H,8.507393701,-0.5682927713,0.367355182
H,7.5324017181,-0.4152269372,-1.1061098941
H,-8.0508881037,2.2970108582,0.3254694133
H,-6.5428117526,2.2806714732,-0.5905932688
H,-6.5108323129,2.1638201185,1.1761667813
H,-8.9676569798,0.4667640639,-1.0538572655
H,-8.1460284432,-1.0901683015,-1.1989347199
H,-7.4692234588,0.3368362552,-1.9899894826
H,-8.9182290821,0.2988937476,1.4884397496
H,-7.3849451983,0.0486426411,2.3400680783
H,-8.0963655621,-1.2620511103,1.3939580796

TS_2(7a)
C,2.0284292791,-0.6453556063,-0.3718503514
C,1.5010303266,-1.639690183,0.4884102249
O,0.1456387804,-1.8406100989,0.5864677342
C,-0.5268702171,-2.4007166202,-0.5817001249
C,-1.6651757563,-1.5396986395,-1.0634214882
C,-1.4097777559,-0.2854740704,-1.6796476058
C,-0.0605490626,0.2047072597,-1.6333370982
C,1.0796517408,0.0873847412,-1.151916241
C,3.4168998041,-0.4720629017,-0.4227694266
C,4.2977123147,-1.2225492923,0.3646391336
C,3.7385384807,-2.1690164527,1.2312173205
C,2.3603644965,-2.3809220079,1.2861593265
C,-2.9767887815,-1.9699582823,-0.8956061914
C,-4.0474987976,-1.1931976061,-1.3411846405
C,-3.7978968821,-0.0067775401,-2.0319612834
C,-2.4913938545,0.4434233623,-2.1892550681
O,-5.3219166642,-1.658820622,-1.1144758076
N,1.9914837877,2.114779807,-1.5186790938
N,0.9974629034,2.5279595302,-2.1638894286
N,-0.0089852145,2.1762936372,-2.6210851708
C,2.546055355,3.0071712293,-0.4826020504
C,1.5063064085,3.6477557609,0.4322571378
O,0.8696458552,2.6388059134,1.1897408197
C,-0.483604978,2.9053907303,1.5380380421
C,-1.0524268374,1.6290496201,2.1433220127
O,-2.4706527405,1.7149010104,2.0912245095
C,-3.1173855267,0.4728510762,2.3296681145
C,-4.5219267979,0.5464448424,1.7443057341
C,5.8162601802,-0.9945957101,0.2440506067
C,6.2699020374,-1.3251922284,-1.1971951628
C,6.1511145483,0.4819948413,0.5577077977
C,6.6181585404,-1.8806508611,1.214975504
O,-5.0095779546,-0.7888619508,1.6818226901
C,-6.261295503,-0.9674951938,1.0465406612
C,-6.2443100899,-0.7704518453,-0.4703962972
H,-0.9027442091,-3.3826433265,-0.284386833

H,0.2089986676,-2.5376127704,-1.3775787183
H,3.7952292224,0.2693767279,-1.1147749339
H,4.3720710213,-2.7666379621,1.87296153
H,1.9387799811,-3.1231133773,1.954173209
H,-3.1908111412,-2.9004092208,-0.3826261643
H,-4.6191987455,0.5741239019,-2.4350437199
H,-2.3023691257,1.3815909985,-2.691932165
H,3.1514396715,3.7895011677,-0.9554415689
H,3.2160468259,2.3793213886,0.1051675683
H,0.7715997133,4.2008583493,-0.1694461473
H,2.0093061336,4.3731093022,1.0894143799
H,-1.0658219987,3.1541939541,0.6415488323
H,-0.5512668013,3.7489813662,2.2387985113
H,-0.706624458,1.4925907379,3.1773184738
H,-0.6995423946,0.7786147901,1.5494416794
H,-3.1482851669,0.2464184409,3.4041725132
H,-2.5816825817,-0.3387492377,1.8248631236
H,-4.4481570109,0.997266058,0.7485603269
H,-5.1864584095,1.1698711508,2.3590560978
H,7.3478087043,-1.1667274511,-1.3032637303
H,5.7651689781,-0.6966489004,-1.9345769785
H,6.053662392,-2.3683141404,-1.4438787813
H,7.230107667,0.6503299763,0.4851418974
H,5.832829744,0.7484083947,1.5694956623
H,5.6634157337,1.165794648,-0.1410237816
H,7.6865053166,-1.6811761882,1.0951018544
H,6.4593535555,-2.9449525692,1.0214464386
H,6.3599177123,-1.6790814677,2.2583273341
H,-6.5540823349,-1.9956319627,1.2698600869
H,-7.0220317109,-0.2928763263,1.4728867929
H,-7.2358784964,-1.013101293,-0.8620853604
H,-6.0249078798,0.2666091919,-0.7362522683

8a(**ht**)
C,-3.0403014453,-0.0963356996,-0.5802357762
C,-2.9918677228,-1.4530440482,-0.9506591564
O,-2.0855439851,-2.1172590614,-1.7438174968
C,-0.9593316626,-1.5116245804,-2.3924949106
C,0.1931001891,-1.2945203576,-1.4417202311
C,0.2803706856,-0.1032517449,-0.6963682948
C,-0.6848112733,0.9707481433,-0.9343919843
C,-2.0712187697,0.9624274845,-0.9304302451
C,-4.1368358459,0.329777111,0.2044821488
C,-5.1536234911,-0.5065737682,0.6446782224
C,-5.0570136767,-1.8577294431,0.272047887
C,-4.0081924755,-2.3112492189,-0.5033069759
C,1.1526781567,-2.2863334765,-1.2673485555
C,2.1900786744,-2.1285404339,-0.3400819751
C,2.2303965431,-0.9797975807,0.4552549227
C,1.2843945206,0.0213937211,0.2649209958
O,3.1048596723,-3.1397130509,-0.2823074279

C,5.2643051548,-2.0546747312,0.1924107911
C,4.1859481381,-3.0418829969,0.6510276489
N,-2.4978280031,2.2322316888,-1.2159319288
N,-1.4744680104,3.0017577622,-1.4222761913
N,-0.3565797227,2.2605871139,-1.2549854267
C,0.9236786945,2.8459366424,-1.6540660229
C,1.5882868793,3.7653956859,-0.6179134796
O,2.3575865938,3.1037205669,0.3761524649
C,3.5899629064,2.5698607326,-0.0946309502
C,4.4084679502,2.1222136941,1.1105779654
O,5.3125928589,1.1221600248,0.6618960496
C,6.2493083746,0.7018100759,1.6458658057
C,6.8993075705,-0.6034866753,1.2143661639
O,6.0310748461,-1.7234003642,1.3471997041
C,-6.3351499151,-0.0176830956,1.4991466696
C,-6.2582244829,1.4910175324,1.7947470198
C,-7.6599736586,-0.2947969979,0.7505510736
C,-6.343824628,-0.7741460068,2.8480827796
H,-0.6833185246,-2.2396698331,-3.1559015587
H,-1.2574376234,-0.5874558615,-2.8914574959
H,-4.152013115,1.3806843066,0.4549869541
H,-5.8114548438,-2.5705740098,0.5850221753
H,-3.9465579067,-3.3512163142,-0.8008579521
H,1.1129793527,-3.2016381179,-1.8469804436
H,2.996864087,-0.8415672689,1.2039367147
H,1.333795252,0.920030016,0.8668821136
H,4.8033700854,-1.1611265942,-0.2340839279
H,5.9057212519,-2.5117019551,-0.572960069
H,4.5841359653,-4.0532373643,0.7259574006
H,3.8203698674,-2.7486464032,1.6391044599
H,0.714190295,3.4359179362,-2.5483233739
H,1.5874541489,2.0296430608,-1.9396586288
H,0.8219823898,4.3325040352,-0.0878589493
H,2.2264625187,4.4768489207,-1.1609097271
H,4.1322350165,3.326647102,-0.6774743891
H,3.4281814342,1.6957068863,-0.7356921482
H,3.733157763,1.7164861096,1.8759399951
H,4.9505018695,2.9675884439,1.5549639176
H,5.7464848497,0.5465482726,2.6102793165
H,7.0250909058,1.4710299592,1.7835391745
H,7.752555814,-0.7962434625,1.8713972685
H,7.2742972365,-0.5167293735,0.1869597614
H,-7.1204942124,1.7908328742,2.3970798922
H,-6.2709720754,2.0866761827,0.8782369701
H,-5.3568776444,1.7499569274,2.3570499383
H,-8.5129838769,0.0410822042,1.3488644015
H,-7.7958374808,-1.3596226575,0.5461241313
H,-7.6847144445,0.2353866333,-0.2054040132
H,-7.1851481039,-0.4432324684,3.4654220116
H,-5.4203114631,-0.5888737014,3.4037837639
H,-6.4382794688,-1.8537409131,2.7079939826

8a(**hh**)
C,-1.9716353558,-0.3982194115,0.1350206436
C,-1.4706779961,-1.1431355629,-0.9410729475
O,-0.1143811579,-1.2539620805,-1.1105413196
C,0.4717072311,-2.2160991983,-0.1910726844
C,1.5602426532,-1.6041014878,0.636660292
C,1.3347243179,-0.56096508,1.5631115026
C,0.1183970678,0.2860336535,1.674037032
C,-1.1080119081,0.4236634508,1.0016980821
C,-3.3576322371,-0.4386065452,0.3625991899
C,-4.249888453,-1.1171780922,-0.4750327475
C,-3.710095536,-1.7760732913,-1.5853544182
C,-2.3345681077,-1.803094885,-1.8044162582
C,2.8499936358,-2.1028185246,0.4429551938
C,3.9334400038,-1.6141109882,1.1610750533
C,3.7041761939,-0.6866271008,2.1753164626
C,2.4299231917,-0.1704468769,2.3588087875
O,5.1965567196,-2.0754099587,0.8620106202
N,-1.5968649093,1.6330842419,1.457825944
N,-0.7706298437,2.1655268739,2.3766017945
N,0.229032924,1.3673857773,2.5204812062
C,-2.5092294531,2.568585626,0.7882622571
C,-1.7427550457,3.6328846064,-0.0106165588
O,-0.9646840969,3.0427619088,-1.0386906912
C,0.4522850146,3.1501997442,-0.8755598464
C,1.104725201,2.1882354182,-1.8583538473
O,2.4881799823,2.1203508145,-1.5348575742
C,3.1436976532,0.9867383644,-2.083827993
C,4.4786404896,0.8131079086,-1.3694895835
C,-5.7546663796,-1.1242772715,-0.1459810838
C,-5.9745198006,-1.7902774228,1.2324020646
C,-6.2877879003,0.3266063714,-0.1038704126
C,-6.5742004852,-1.9029732689,-1.1912639621
O,4.9242743845,-0.5096925079,-1.6500910429
C,6.1602844611,-0.8921397317,-1.0758027946
C,6.1460307885,-1.0838782651,0.4424328576
H,0.8714977899,-3.0379714447,-0.7891360568
H,-0.3223924371,-2.6162527219,0.4449364465
H,-3.7397077728,0.0702310944,1.2390198771
H,-4.3521874628,-2.3003662572,-2.2803453575
H,-1.9139800246,-2.3474928019,-2.6418946719
H,3.0313258245,-2.8515225886,-0.3187560805
H,4.5211684706,-0.3426772118,2.7994891567
H,2.2738312803,0.5942495733,3.1058817356
H,-3.1229595591,3.0523173573,1.5521936227
H,-3.1536499452,2.0034244614,0.1207942349
H,-1.1202669189,4.2248116975,0.6664629656
H,-2.480905084,4.3002754793,-0.4711842508
H,0.7535437728,2.8701798397,0.1385206011
H,0.7794973141,4.1818050898,-1.0600923608

H,0.9606270895,2.5262508221,-2.894122147
H,0.640382068,1.2018937361,-1.7504402286
H,3.2898729359,1.1038622076,-3.166243117
H,2.546208791,0.0843607845,-1.9156595887
H,4.3129579471,0.9650527533,-0.2975170398
H,5.2193457851,1.5478989268,-1.7143973373
H,-7.0401957893,-1.8066194334,1.4802658048
H,-5.4556605295,-1.2546409357,2.0307034888
H,-5.6102882611,-2.8212940146,1.230411891
H,-7.3589765396,0.3312128345,0.1188658328
H,-6.1395861131,0.8260113851,-1.0653035057
H,-5.7898138233,0.9211617033,0.6656572699
H,-7.6321624363,-1.8788000507,-0.9171547397
H,-6.2730971306,-2.9526798366,-1.2460325349
H,-6.4831230241,-1.4663324853,-2.1897344611
H,6.4166137598,-1.8410507402,-1.5525892118
H,6.9496775047,-0.1610781081,-1.3185532396
H,7.1299260347,-1.4481931738,0.7503726177
H,5.9603332359,-0.1402071493,0.9630491668

ODIBO-O-$(CH_2)_{11}$-N_3 (**13**)
C,9.8541019526,-0.4301559802,0.155456321
C,9.3485089342,-1.7170579282,0.4028872971
C,7.9820704279,-1.9767941873,0.4483213869
C,7.0638343525,-0.9493321747,0.2577070441
C,7.54489796,0.3620512953,0.0125428308
C,8.9272431057,0.5943501808,-0.0401492566
C,11.3751417495,-0.1970910466,0.1114713949
C,11.9962221733,-0.5919231293,1.4717744497
C,11.9972300925,-1.065635523,-1.0068334032
C,11.7334434787,1.2737008407,-0.169234244
O,5.7244617389,-1.2169995543,0.4076200254
C,4.8144523018,-1.0168826796,-0.7153635336
C,3.6861688097,-0.0430416484,-0.4426825458
C,3.9595559903,1.3442303478,-0.2574099458
C,5.3450123607,1.642478284,-0.1924107451
C,6.515599323,1.3240727591,-0.1585612341
C,2.3713149565,-0.4771368601,-0.4398976341
C,1.3100178738,0.4282343492,-0.2784934062
C,1.5780589708,1.789800397,-0.1167973387
C,2.899349493,2.2362825408,-0.1037614944
O,0.0641629314,-0.1206167523,-0.2962729865
C,-1.0747648233,0.7279179803,-0.1257472035
C,-2.3191862907,-0.144335857,-0.1711418918
N,-15.0527706176,-0.4232163653,0.6213784497
N,-15.4195116656,-0.900124569,-0.4496277047
N,-15.8638820222,-1.3895750623,-1.3737202133
C,-3.6083713874,0.6663729905,0.0049846149
C,-4.873138808,-0.1993359618,-0.0339577403

C,-6.1679304404,0.6016988822,0.1424215924
C,-7.4325480014,-0.263893637,0.1057467783
C,-8.7282140025,0.5358519964,0.2831344952
C,-9.9916495505,-0.3314410078,0.2493391664
C,-11.2870879327,0.4691889119,0.4272732319
C,-12.5464727799,-0.405001912,0.3967871059
H,-3.6713089185,1.4311146506,-0.7797695311
H,10.0311247875,-2.5432951963,0.5645675451
H,7.6112198508,-2.9753966889,0.6479649484
H,9.2530359009,1.6075463126,-0.2311403008
H,13.0790567711,-0.4333157727,1.455142901
H,11.8175044015,-1.6428545988,1.7107825728
H,11.5762890015,0.010900129,2.2816382343
H,13.0802664301,-0.9130844725,-1.0482672214
H,11.5791730192,-0.803016975,-1.9825678239
H,11.8173360976,-2.1305644513,-0.842075302
H,12.8206637224,1.3876548677,-0.1906285282
H,11.347794868,1.9420764781,0.6054454233
H,11.3482781494,1.6099234596,-1.1358144323
H,4.3956590668,-2.0001976813,-0.9416215766
H,5.3912136258,-0.6791735273,-1.5799605622
H,2.1331488382,-1.5270922279,-0.5686667205
H,0.7769870633,2.50554633,0.0062087236
H,3.1072832306,3.2904102505,0.0355696835
H,-1.0972059756,1.4797810058,-0.9249450409
H,-1.002502972,1.2521799638,0.8356070476
H,-2.2431021562,-0.9040800298,0.6138565557
H,-2.3388217854,-0.678427238,-1.1269997627
H,-4.8106015065,-0.9645843293,0.7500354815
H,-4.9096008011,-0.7424631204,-0.9867876106
H,-6.2302508923,1.3666662141,-0.6423669504
H,-6.1298408026,1.1459988779,1.0949018221
H,-8.7923540532,1.2998198316,-0.5024410823
H,-8.6891915335,1.0812456475,1.2349173395
H,-9.9283253436,-1.0947864816,1.0351004829
H,-10.0316413365,-0.8767934447,-0.7021092617
H,-12.4954967723,-1.1635411816,1.1850654015
H,-12.5954058375,-0.9442074115,-0.5568093292
H,-3.5731216726,1.208893528,0.9584132386
H,-7.3697034141,-1.0290357202,0.8901758852
H,-7.4709200491,-0.8081524629,-0.84663707
H,-11.3516470563,1.2314699509,-0.3596137416
H,-11.2467038749,1.0147413142,1.3786726273
C,-13.8274805683,0.4116233476,0.5833511555
H,-13.8123510087,0.9295141648,1.5428401603
H,-13.9202981482,1.1714837679,-0.2024910438

TS_1(13)
C,3.0073762196,-0.3945872658,0.2262128363
C,2.6858859525,-1.7208201342,-0.147024445
O,1.4465033487,-2.2692314802,0.0768821599

C,0.2791508992,-1.6906724824,-0.5681728161
C,-0.7635645873,-1.2729422395,0.438227996
C,-0.5013101819,-0.1874566836,1.3062804017
C,0.7717188807,0.4472491277,1.1600541261
C,1.9516381288,0.4060122727,0.7745309942
C,4.3265730121,0.0556794286,0.0450639378
C,5.3313706958,-0.7608413059,-0.4731705029
C,4.9768429003,-2.072086627,-0.8280586111
C,3.6778179314,-2.5424921593,-0.6749616452
C,-1.9807009015,-1.9504798798,0.5127901743
C,-2.951014221,-1.5731051008,1.448191569
C,-2.6755271811,-0.5230472235,2.3330375048
C,-1.4707487497,0.157993145,2.2599509917
O,-4.1685846408,-2.1707182759,1.6023662885
N,2.6500654749,2.5376908822,0.9604438867
N,1.6145746258,2.9630659895,1.258396703
N,0.4603700113,2.6368831961,1.6163900476
C,-0.6894477239,3.4169424061,1.1291885703
C,-0.69947366,3.7083273509,-0.3823970773
C,-2.1302406724,3.7811755658,-0.9478111259
C,-2.7321857828,2.3842345403,-1.1541720556
C,-4.2400497633,2.3410246196,-1.4242837067
C,-4.7475051909,0.8970169626,-1.5325990853
C,-6.2710554472,0.7556515591,-1.6023380047
C,-6.7850015368,-0.6955478377,-1.6466566858
C,6.7811335447,-0.2781124405,-0.6605728742
C,6.9740564308,1.1816282659,-0.2109868863
C,7.7324740264,-1.1677707947,0.1733809937
C,7.1681524754,-0.3766017998,-2.1547211501
C,-6.3916312667,-1.5630559636,-0.4280979658
C,-5.1519299703,-2.4463160448,-0.6477155494
C,-4.643515874,-3.1145297553,0.629568751
H,-1.2609807289,0.9625623821,2.9539199358
H,-0.1241171345,-2.4614062167,-1.2304972434
H,0.5911977403,-0.8386406801,-1.1769813768
H,4.5375785532,1.0735325545,0.3354091316
H,5.7227846975,-2.7463435958,-1.2330532765
H,3.4168182252,-3.5591482936,-0.945048674
H,-2.1560500938,-2.7740769141,-0.1673322606
H,-3.4229345051,-0.2597414688,3.0719232361
H,-0.7663544389,4.3454902538,1.706289878
H,-1.5504686762,2.8076337836,1.4053641665
H,-0.1514444769,2.9151671484,-0.9018060832
H,-0.1583508408,4.6396799325,-0.5756870251
H,-2.1235972937,4.325062793,-1.8983551892
H,-2.7640658719,4.3672574475,-0.2691491521
H,-2.5307893707,1.7691711525,-0.2698077445
H,-2.2008961271,1.8941141008,-1.979991876
H,-4.4817028989,2.8982066751,-2.3382463316
H,-4.7677322895,2.8486804272,-0.6062982541
H,-4.374101478,0.3475742397,-0.6622996877

H,-4.2952199238,0.4197096166,-2.4120460764
H,-6.6480523351,1.2912241131,-2.4823490151
H,-6.713663169,1.2552852871,-0.7305767608
H,-6.4378148275,-1.1795037897,-2.5687034722
H,-7.8766307122,-0.6609651239,-1.7231408489
H,8.0160823551,1.4764727106,-0.3632229255
H,6.7446650304,1.3149673298,0.8495581192
H,6.3484748035,1.8710520784,-0.7841986964
H,8.7685610978,-0.8372400345,0.0489517643
H,7.6791003489,-2.2162597926,-0.129289772
H,7.4835246565,-1.1139460069,1.2367479841
H,8.1989668795,-0.0397364649,-2.303592049
H,6.5140324093,0.2490201618,-2.7684151412
H,7.0959083271,-1.4013647359,-2.5269307307
H,-7.2292105145,-2.2163606334,-0.1586925866
H,-6.2251804588,-0.9170740396,0.4405521062
H,-4.3428356729,-1.8654460067,-1.0980539925
H,-5.3921479854,-3.2405867782,-1.3656707066
H,-5.4572953704,-3.6324495248,1.1417543193
H,-3.8673281364,-3.8556389057,0.4134995623

TS_2(13)
C,-2.1702168772,-0.962588278,0.2055601731
C,-1.8126203126,-2.1546440837,-0.4770964632
O,-0.5632992676,-2.7077508339,-0.3687005569
C,0.5909847773,-1.9396449921,-0.7972238233
C,1.6139523232,-1.7416684081,0.2924140935
C,1.3014384208,-0.9753354793,1.4434009371
C,-0.0091621162,-0.3965869039,1.4921818274
C,-1.1353870238,-0.3354314175,0.9671385293
C,-3.500215056,-0.530140911,0.1389323669
C,-4.4857841204,-1.2205781545,-0.5755764495
C,-4.0942896973,-2.3845533164,-1.2461308427
C,-2.7774312766,-2.8421439115,-1.1999167556
C,2.9059927877,-2.2290947379,0.1146467157
C,3.9130046067,-1.9335012336,1.0386822409
C,3.5861173904,-1.2517012657,2.2134387121
C,2.2969534341,-0.7774582601,2.4106282192
O,5.2274479958,-2.2819263928,0.8720574724
N,-1.803614953,1.7524822374,1.6127437166
N,-0.8257981731,1.9114383939,2.3755532262
N,0.1090597684,1.3385560035,2.769055209
C,-2.4883752522,2.9197191498,1.0292670465
C,-1.6250177399,4.1100700303,0.5831083726
C,-0.6673686182,3.8991904679,-0.6060702492
C,0.656308005,3.200962167,-0.2685638799
C,1.6645605172,3.1617185554,-1.4255099226
C,2.9636102595,2.4495560401,-1.0245851857
C,3.9764186172,2.2591164087,-2.1626673573
C,5.2385143344,1.4719231193,-1.7568285398
C,-5.9344424586,-0.6978073854,-0.5836028961

C,-6.4778366423,-0.6610271207,0.8638914222
C,-5.9691234454,0.7289283442,-1.1788620392
C,-6.8702140973,-1.5867100638,-1.4233693903
C,4.9305998001,0.0349635472,-1.3182304765
C,6.1506000474,-0.7977126265,-0.9030637706
C,5.8304475214,-2.2224344476,-0.4353598209
H,1.0377961241,-2.4913906496,-1.6284252732
H,0.2519238542,-0.9696620227,-1.1722582104
H,-3.7564085503,0.3551250021,0.7044238388
H,-4.8141899637,-2.9604726801,-1.8121514545
H,-2.4879735024,-3.7540058931,-1.7090619759
H,3.1330780158,-2.7967217864,-0.7793928514
H,4.3684060858,-1.0591161646,2.9377628622
H,2.0622604189,-0.2028766196,3.2964560187
H,-3.2408627512,3.2729164643,1.7454196278
H,-3.0333509777,2.5169066081,0.1725379168
H,-1.0656236475,4.4908426258,1.4458148312
H,-2.3390286378,4.8985258802,0.3172013982
H,-0.4394367813,4.8870400627,-1.0243129788
H,-1.1876292546,3.3494630462,-1.401004069
H,0.4646796828,2.1754952452,0.0590860172
H,1.1185525095,3.7121999601,0.5853910417
H,1.8845689339,4.1824699069,-1.7643054459
H,1.2157673276,2.6445241915,-2.2839585631
H,2.6951200831,1.475598695,-0.6041948374
H,3.4418723006,3.0059500279,-0.2078364898
H,4.2759950552,3.2406420818,-2.5491082724
H,3.4876481911,1.7404770055,-2.9982787636
H,5.7489947443,1.9999543188,-0.9408972321
H,5.9401236503,1.4595278585,-2.5999457583
H,-7.5074249826,-0.289537168,0.8743490033
H,-5.8812042678,-0.0088020389,1.505828472
H,-6.4720494201,-1.6603607075,1.3074496191
H,-6.9950866716,1.1098729179,-1.1956034942
H,-5.5875219296,0.734146301,-2.2037136082
H,-5.3663399328,1.4266151304,-0.5927389992
H,-7.8831909019,-1.1757417619,-1.4006576271
H,-6.9207278484,-2.6070587987,-1.0338154289
H,-6.5572687722,-1.6337634013,-2.4701509819
H,4.4069867772,-0.4811418899,-2.1342756179
H,4.2307993877,0.0696145437,-0.4844434399
H,6.702238248,-0.2899440382,-0.10353267
H,6.8435976558,-0.8869591844,-1.7502451552
H,5.2123550112,-2.7481087225,-1.1708156435
H,6.7535622576,-2.7916475871,-0.31728266

14a

C,2.8854076076,0.066165534,-0.4183676371
C,2.5447742385,-1.1268357237,-1.0808927652
O,1.3491107957,-1.5010886737,-1.6493577793
C,0.1699683912,-0.6876022894,-1.6614970532

C,-0.5596759872,-0.7169905545,-0.3392678351
C,-0.2614820197,0.2214730921,0.6589463947
C,0.7113470625,1.2817175166,0.3785855171
C,2.0029891565,1.2160767814,-0.1266805954
C,4.223055501,0.2130944876,0.016199218
C,5.2097891595,-0.7483965054,-0.15533932
C,4.8232335822,-1.9330480415,-0.8039576805
C,3.5294580095,-2.1111805607,-1.2530623738
C,-1.5188407949,-1.7089867616,-0.1092700028
C,-2.1819162236,-1.7875122921,1.1176716778
C,-1.8516507307,-0.8768111033,2.131359763
C,-0.9068795923,0.1084556716,1.9014910638
O,-3.1316789704,-2.7073101766,1.4438932861
N,2.4831444765,2.4948900908,-0.2131903665
N,1.5714369118,3.3320338804,0.18238841
N,0.4875780913,2.6218762971,0.5473175456
C,-0.7538358227,3.3269415192,0.8682816811
C,-1.4386564496,3.9612623986,-0.3648460665
C,-2.9711674347,3.8242420461,-0.3202883807
C,-3.4265193378,2.3999122285,-0.6638214976
C,-4.9016334859,2.0809066133,-0.3956645485
C,-5.2028624518,0.5999383466,-0.6603194661
C,-6.6079622529,0.1409212499,-0.2599288538
C,-6.8769458424,-1.3626588326,-0.4613205859
C,6.65842895,-0.5621726034,0.3264565195
C,6.8747750383,0.7966925174,1.0166339956
C,7.0149878574,-1.6782826586,1.3358589283
C,7.6163556221,-0.6451479976,-0.8851342892
C,-5.9499783688,-2.3044163877,0.3421550555
C,-4.7302724866,-2.8230749181,-0.4388107315
C,-3.709815534,-3.5526108629,0.4343767904
H,-0.6524099929,0.7945109221,2.7015812447
H,-0.4458635923,-1.1438122176,-2.4376545871
H,0.4109871358,0.3306531583,-1.9712795777
H,4.455529137,1.1478968222,0.5053704815
H,5.5397304163,-2.7300700556,-0.9674962821
H,3.2397797712,-3.0203408552,-1.7663422518
H,-1.7362632421,-2.4112857879,-0.9035123511
H,-2.3481372573,-0.9649533599,3.0901106031
H,-0.5095307018,4.0877685074,1.6112850842
H,-1.4134231519,2.6020350297,1.3413625549
H,-1.0603297508,3.4754399848,-1.269504915
H,-1.1431736948,5.0103595712,-0.4372646788
H,-3.4208148344,4.5364951597,-1.0202468696
H,-3.338325137,4.1067922738,0.6752488131
H,-2.8218822181,1.6820219658,-0.0979470404
H,-3.2003413635,2.2070691346,-1.7204707692
H,-5.5495746393,2.718363681,-1.0101054912
H,-5.1392509977,2.3166614953,0.649825306
H,-4.4625793199,0.0107398331,-0.1098294865
H,-5.0373432254,0.3820481556,-1.7238728534

H,-7.3544960002,0.7122421618,-0.8250815383
H,-6.7745292228,0.386190293,0.7972144829
H,-6.81257577,-1.6073048223,-1.5294586818
H,-7.9155385754,-1.5570421663,-0.1747660199
H,7.9172126535,0.8835156666,1.335870209
H,6.2471688248,0.9057637571,1.9053475393
H,6.663065492,1.6329257165,0.3452522019
H,8.0468287204,-1.5627648896,1.6827871345
H,6.9228380574,-2.6726713924,0.8921977687
H,6.3563480117,-1.6392069318,2.2079730962
H,8.6542461942,-0.5214368674,-0.5596755439
H,7.3917034266,0.1396040381,-1.6126147767
H,7.5405555564,-1.6074302552,-1.3974789902
H,-6.5230837324,-3.1715752551,0.6892310903
H,-5.6079263239,-1.791066833,1.2471410632
H,-4.236064482,-2.003624661,-0.9664745883
H,-5.0628659321,-3.5252477881,-1.2135037193
H,-4.1918912383,-4.3480332032,1.0063561836
H,-2.9201146664,-4.0120753138,-0.1685058625

14b
C,-1.4228174621,-0.9512535273,-0.9283689353
C,-1.0419265789,-1.2017699635,-2.2585912691
O,-0.2797540881,-2.3095852584,-2.5442903025
C,1.1073665995,-2.0617857994,-2.7654774558
C,1.9113353021,-1.6050798946,-1.5600222338
C,1.6316916872,-1.9772517133,-0.2302205502
C,0.3269213309,-2.4284313018,0.2922594246
C,-0.9404665196,-1.8744658913,0.1087716701
C,-2.2384764052,0.1619936096,-0.6803114215
C,-2.7066071551,1.004086533,-1.6982474621
C,-2.319798974,0.7038475258,-3.0083970263
C,-1.4962834837,-0.3841858969,-3.2845956056
C,3.0888074964,-0.8999157361,-1.8361466775
C,4.0141649857,-0.6111251548,-0.8356359412
C,3.8226583944,-1.1323094711,0.442021115
C,2.635425251,-1.7842263436,0.7354770389
O,5.1308220894,0.1524873704,-1.0908266101
N,-1.6881585296,-2.4473471075,1.1031152271
N,-0.942055835,-3.3033906688,1.8260377229
N,0.2656938503,-3.288425782,1.3483098981
C,-3.0530440606,-2.1842816175,1.5717207513
C,-3.1103167593,-1.5495317833,2.9761796464
C,-2.5698693271,-0.1143542653,3.1379030936
C,-1.0378144872,0.0286922134,3.1148273737
C,-0.5352171642,1.4773636848,3.0118246712
C,0.9671244557,1.5523118041,2.6976665884
C,1.4747596949,2.9567017862,2.3383016098
C,2.9353809679,2.9924675009,1.8488952818

C,-3.6056488409,2.2070273453,-1.3541006965
C,-4.869048059,1.7180326847,-0.6084716677
C,-2.8271444228,3.1927185457,-0.4529429126
C,-4.0633864137,2.9695507895,-2.6115053005
C,3.1536206735,2.3472480759,0.4713299295
C,4.6258118267,2.3354747535,0.0268201681
C,4.9269656607,1.5717398615,-1.2701834569
H,1.5002742129,-3.0240294025,-3.105235726
H,1.2334860795,-1.3427574791,-3.5840996138
H,-2.4974562012,0.3862825783,0.3446398602
H,-2.6592413721,1.3134121262,-3.834902529
H,-1.2149627869,-0.6178993194,-4.3052160039
H,3.2942422391,-0.570872457,-2.8499264076
H,4.5716334134,-0.9602316472,1.2055311074
H,2.4405655198,-2.122028186,1.7456473758
H,-3.5700962843,-3.146036437,1.5962918634
H,-3.5448938036,-1.5624334269,0.8246489128
H,-2.5974016416,-2.22102907,3.6707019848
H,-4.1685217557,-1.5583875181,3.2614501056
H,-2.947439016,0.2800926314,4.0884866494
H,-3.0132475737,0.5281758351,2.3665817822
H,-0.6265212778,-0.521238402,2.2672279364
H,-0.6199990882,-0.4500703981,4.0081929018
H,-0.7622294885,2.0311660234,3.9315154971
H,-1.0831907142,1.9864797716,2.2086892753
H,1.1707310325,0.8708368354,1.8654206851
H,1.541813612,1.1661423217,3.5492354765
H,1.3739549025,3.6121493332,3.2115941976
H,0.8285379723,3.3858332445,1.5611564896
H,3.5738620097,2.4903392101,2.5876057943
H,3.2758237279,4.0347051588,1.8145050645
H,-5.5195877286,2.5647958257,-0.3695703847
H,-4.6215425624,1.21850845,0.3310551129
H,-5.4384667178,1.015545062,-1.2233460845
H,-3.453257228,4.0546453819,-0.2024720923
H,-1.9304220884,3.5597299288,-0.959652884
H,-2.5140729305,2.7264564107,0.4835740201
H,-4.7043827202,3.8049739445,-2.3178395634
H,-4.6409881883,2.332955211,-3.2874167498
H,-3.2187895859,3.3850642538,-3.1677546161
H,2.5594124828,2.887652724,-0.2771967709
H,2.7694194281,1.3267119135,0.484699601
H,5.259428382,1.9172461935,0.8174024025
H,4.9632737209,3.3701576457,-0.1156268798
H,4.139096204,1.7388473571,-2.0153123358
H,5.871677114,1.914391464,-1.6947441399

TS for the head to tail dimerization of 7a

C,-1 1.1372047999,-7.7739929418,0.1851128779

C,-12.2710463717,-6.9699066566 ,-0.0351625045
C,-12.1691263786,-5.5885734509,-0.1903142491
C,-10.9264 393014,-4.9587873289,-0.1188640672
C,-9.762618563,-5.7354948522,0.1112 996535
C,-9.8971845131,-7.1304427855,0.249521398
C,-11.2927559354,-9.2 982462023,0.347090264
C,-12.208927693,-9.5986433749,1.5585333231
C,-11 .9292801394,-9.8920455746,-0.9332643306
C,-9.943440354,-10.0051112983, 0.5819261152
O,-10.8896115696,-3.5857235234,-0.1813150416
C,-10.152689 9547,-2.9449342999,-1.2582352936
C,-9.1063242107,-1.9935842129,-0.7300 601232
C,-7.9717600238,-2.502094355,-0.0333704933
C,-7.9443386128,-3.9 187405166,0.162147029
C,-8.5152278424,-5.0258802465,0.1881122298
C,-9. 2378799393,-0.6238887921,-0.9201258338
C,-8.2672168136,0.2697397046,-0 .4382739211
C,-7.1546260551,-0.2215809411,0.2539370797
C,-7.0183394904 ,-1.5986555405,0.4497622984
O,-8.5033428075,1.592025017,-0.6904445467
C,-7.573644637,2.5607818535,-0.2131375777
C,-8.1076035494,3.9223878495 ,-0.6344520212
N,-5.2467218415,15.4274877187,0.1993167386
N,-4.4781263 586,16.3078052227,0.5947064844
N,-3.8474566124,17.2085104447,0.9057397 255
C,-7.5614708748,6.2242140142,-0.4880339651
C,-6.4929357157,7.14946 77012,0.0762977678
C,-5.950009538,9.451621216,0.2274642397
C,-6.475899 393,10.8174077125,-0.1900736814
C,-5.9317927639,13.1190704242,-0.03742 08312
H,-13.2577043753,-7.4220534078,-0.0890425165
H,-13.0523874121,-4 .9773618149,-0.353256406
H,-8.9915586991,-7.6959903952,0.4204723436
H, -12.3311984368,-10.6820797669,1.6845666654
H,-13.2067949702,-9.1635743 48,1.4346529506
H,-11.7804171493,-9.1942866392,2.4832472527
H,-12.0499 589155,-10.9781282188,-0.8306872347
H,-11.2980243107,-9.7009194177,-1. 8092978624
H,-12.9181318455,-9.465166098,-1.1342865225
H,-10.108608337 5,-11.0837168263,0.6902079222
H,-9.4499299844,-9.6533224159,1.49544872 61
H,-9.2528877459,-9.8594382641,-0.2570677997
H,-10.887867673,-2.3983 621498,-1.858899047
H,-9.6947941094,-3.7155471316,-1.8876180428
H,-10. 1003385995,-0.2179799915,-1.4422985844
H,-6.3979661137,0.4476374443,0. 6478555632
H,-6.1685051733,-1.9789724486,1.0071644813
H,-6.5810664588, 2.3925076407,-0.6515619856
H,-7.4902881094,2.5098055037,0.880452891
H, -9.1053668108,4.0869401643,-0.198735053
H,-8.2011982334,3.9677124002,- 1.7307404982

H,-8.5409037412,6.4685830805,-0.0483801672
H,-7.633831256 6,6.3543943864,-1.5790078494
H,-5.5129166171,6.9030901693,-0.361642559 8
H,-6.42178681,7.0196944647,1.1676045245
H,-4.9496435357,9.2866241315 ,-0.2026077581
H,-5.8626772454,9.4018707738,1.3241735535
H,-7.47569274 15,10.9833063483,0.2401527456
H,-6.5621088049,10.8684459874,-1.2865200 691
H,-6.9132233215,13.3663147205,0.396756959
H,-5.999391243,13.252134 8503,-1.1285627351
C,-4.8616121101,14.0389960643,0.5348630269
H,-4.799 3070964,13.904862838,1.6226717405
H,-3.8855259729,13.7900117057,0.0984 302841
H,20.0847673786,0.0496524751,1.4900705955
C,20.1685294177,0.230 2635127,0.4127858172
C,19.2088557327,1.3485922607,-0.0620337887
H,21.2 09439662,0.5064141475,0.2013908277
H,19.9555145468,-0.7140445788,-0.10 22669483
C,17.7546704623,0.9445957235,0.2471269342
C,19.5492608607,2.6 634297488,0.6812682886
C,19.4479523066,1.5755479527,-1.5676368424
C,17 .3595391233,0.6970838263,1.5759823201
C,16.7790642051,0.8079031476,-0. 7463517045
H,20.584308202,2.962255354,0.4719067425
H,19.4467636572,2.5 57756364,1.7668303137
H,18.8881748175,3.4773467286,0.3608198971
H,20.4 937427282,1.8592983624,-1.7344346226
H,18.8208525311,2.382366563,-1.96 51772809
H,19.2553590992,0.6695053491,-2.1544784471
C,16.0555405241,0. 3240987086,1.9039937278
H,18.0815298445,0.7941162597,2.3821298988
C,15 .4549778314,0.4475778612,-0.4416312865
H,17.0180048853,0.9872025477,-1 .7882055129
C,15.0882749885,0.1954230947,0.907978771
H,15.7700826793,0 .1439696536,2.936641294
C,14.3845717575,0.2882423756,-1.3643119134
O,1 3.7889896009,-0.0761697697,1.2708861451
C,13.2149656611,0.0497115576,- 1.6208091449
C,13.1467597415,-1.2975893474,0.7981030973
C,11.927988120 9,-0.4529240099,-1.2856289889
C,11.8760146007,-1.0713499965,0.00109794 41
H,12.9122262895,-1.8799553027,1.6952878048
H,13.8668225849,-1.86376 28878,0.1975893081
C,10.7596507864,-0.3446344805,-2.0451486331
C,10.65 65409534,-1.5325375746,0.4793298145
C,9.5397725832,-0.8246885852,-1.55 94560569
H,10.8005670121,0.1255270874,-3.023349254
C,9.4861413103,-1.4 142556453,-0.291151215
H,10.5838813397,-1.9996148399,1.4580438187
H,8. 6497071235,-0.72616701,-2.1703979942
O,8.3503081906,-1.9071832755,0.28 659751

C,7.1187419216,-1.8160150487,-0.4250790386
C,6.0514279837,-2.42 8676004,0.4705275099
H,7.1785675619,-2.3675213711,-1.3726174845
H,6.87 46453317,-0.7675720102,-0.6408373011
H,5.9963448601,-1.8773703464,1.42 21434684
H,6.30473432,-3.476334898,0.6961417054
C,3.7250660845,-2.8819 456714,0.4926131593
C,2.4852654475,-2.729981975,-0.3769614765
H,3.5867 508798,-2.342314987,1.4423607602
H,3.8940053589,-3.9450204761,0.724311 2875
H,2.6229740309,-3.2716905744,-1.3258904589
H,2.318936793,-1.66686 50161,-0.6116167749
C,0.1591318741,-3.1852378795,-0.3545791292
C,-0.91 69854474,-3.7842241954,0.5396273658
H,0.2166779582,-3.7461195346,-1.30 05611443
H,-0.089407629,-2.1390430649,-0.5925533412
H,-0.9750133657,-3 .2235347153,1.4855571454
H,-0.6692341799,-4.8304658372,0.7769864669
C, -3.2449214533,-4.2360304541,0.5626464363
H,-3.3945336721,-3.6805278402 ,1.5021483489
H,-3.0659585862,-5.2931032482,0.8186723471
C,-4.48697638 48,-4.1103412125,-0.3214003003
N,-5.7039704226,-4.5291895019,0.3867854 234
H,-4.6402346068,-3.0597365919,-0.581511686
H,-4.3488407356,-4.6713 214605,-1.2535726238
N,-5.9812930469,-5.7545616754,0.4118271052
N,-6.8 631363411,-6.5124166372,0.4733404921
O,-7.1876032164,4.8930144216,-0.1 671109648
O,-6.8661277473,8.4788486038,-0.2469094182
O,-5.5591282666,1 1.7918711587,0.2851998871
O,4.8232918088,-2.3473020419,-0.2306221859
O ,1.3884385499,-3.2606249448,0.3484160021
O,-2.14840419,-3.7088016526,- 0.1630845012

TS for the head to head dimerization of 7a

C,-2.6959927428,6.7315576285,1.7737519749
C,-3.4810485009,6. 8026088597,2.9397740027
C,-4.602931956,5.9956757267,3.1149551272
C,-4. 9885178535,5.0916771286,2.1246577097
C,-4.2215537615,4.9904140593,0.93 47745281
C,-3.0890404925,5.8150814827,0.7937035307
C,-1.4687486845,7.6 496878566,1.6164858843
C,-1.9198417898,9.1296272148,1.6782965127
C,-0. 4654075944,7.3736966028,2.7628621692
C,-0.7360408315,7.4307270667,0.27 86282377
O,-6.1579056024,4.3967837502,2.3030855608
C,-6.1238128559,2.9 425474587,2.3113924009
C,-7.0305119982,2.3419808232,1.2664059817
C,-6. 7149549926,2.4649588318,-0.1171763944
C,-5.5567736015,3.2511736993,-0. 448222131

C,-4.6948950652,4.0570224825,-0.0417525996
C,-8.1626376495,1 .6354147082,1.6538081601
C,-8.9996205923,1.0331112328,0.7008651161
C,- 8.6914199172,1.1425285572,-0.6594932068
C,-7.5551071715,1.8525650284,- 1.0552240719
O,-10.0823730652,0.3662689113,1.2016330792
C,-10.98434962 24,-0.2616525261,0.2943316634
C,-12.0760593672,-0.9020787734,1.1396678 012
N,-21.9907564066,-7.3162719613,-0.2023426917
N,-22.8000566833,-7.8 842033572,-0.9401893817
N,-23.613680338,-8.4436115557,-1.5154205767
C, -14.0580425444,-2.1799287544,0.9032270587
C,-14.9336310906,-2.81138359 95,-0.1695822365
C,-16.9149298658,-4.0907257265,-0.4053300362
C,-18.01 15185773,-4.7319584052,0.4328792776
C,-19.9919392777,-6.0121006722,0.1 961357001
H,-3.2206563374,7.5009438257,3.7303375974
H,-5.2081439008,6. 0661777497,4.0143358803
H,-2.5382975598,5.7310043816,-0.1324720173
H,- 1.0548437026,9.7965219988,1.5709557583
H,-2.4080354975,9.3685698049,2. 6293819953
H,-2.6279947229,9.3573662718,0.8728364635
H,0.4123199115,8. 0254163482,2.6663969387
H,-0.1208245481,6.3328824531,2.740487694
H,-0. 9090608273,7.5574150731,3.7474908225
H,0.1240413805,8.1076541833,0.215 5427415
H,-1.3830632946,7.6410947006,-0.5813423027
H,-0.3546443311,6.4 070160909,0.1832308366
H,-6.4481694293,2.632571601,3.3104584351
H,-5.0 90748625,2.6095756377,2.1642332248
H,-8.4210554318,1.5369791521,2.7048 59363
H,-9.3199055737,0.6864558406,-1.416183488
H,-7.3183251014,1.9341 686491,-2.1096159808
H,-10.4664004955,-1.0280806123,-0.2971465372
H,-1 1.4207343672,0.4776822187,-0.3899895194
H,-12.5918771998,-0.1325293449 ,1.7348754788
H,-11.6341135773,-1.6330260547,1.8347344417
H,-14.644369 2223,-1.4544643253,1.4883334206
H,-13.6883721104,-2.9558005203,1.59161 25727
H,-14.346526636,-3.5359477998,-0.7553800496
H,-15.3033722877,-2. 0352510381,-0.8578422065
H,-16.3973752578,-4.8604151253,-0.9991729778
H,-17.3536728828,-3.3595043914,-1.1022695897
H,-18.5308676645,-3.96280 31863,1.0252298358
H,-17.5734705256,-5.4631490506,1.1298071786
H,-20.5 813225199,-5.2886024256,0.7809947724
H,-19.6243917622,-6.7879250813,0. 885993299
C,-20.8623242242,-6.643150049,-0.8824160905
H,-21.2282232646 ,-5.8662252998,-1.5662965694
H,-20.2714415571,-7.3646024793,-1.4616925 922
H,20.57978669,-6.3999909418,1.281259273

C,21.0363027514,-5.7779874 485,0.503513562
C,20.8101631742,-4.268600014,0.7636822458
H,22.1099831 526,-6.0043936108,0.483695774
H,20.6072082931,-6.0784198475,-0.4598026 436
C,19.2987328887,-3.9716598183,0.7925956411
C,21.4380631292,-3.8853 756823,2.1260710635
C,21.5429261085,-3.4791178923,-0.3387464173
C,18.4 733313123,-4.6097195054,1.7385435293
C,18.6854837212,-3.0772441415,-0. 091410349
H,22.5156283441,-4.0934406543,2.1208325852
H,20.9944442942,- 4.4494281138,2.9538321709
H,21.2980958121,-2.8183281986,2.3358455962
H ,22.6110618344,-3.7254488329,-0.31815387
H,21.4524752666,-2.395736342, -0.1960748877
H,21.167467014,-3.7266672104,-1.3388273912
C,17.09918386 78,-4.3753269805,1.8007328986
H,18.9049299268,-5.3101470314,2.44822275 93
C,17.3059812022,-2.8120552967,-0.0408022876
H,19.2652741394,-2.5515 01554,-0.8411616393
C,16.4972776832,-3.4779269686,0.9194825264
H,16.48 15326984,-4.8734545925,2.5428043075
C,16.5814229284,-1.9204187146,-0.8 789870831
O,15.1596106593,-3.1919458926,1.0690556251
C,15.5471221562,- 1.3667640749,-1.2171795743
C,14.2312770742,-3.4874637498,-0.0166516839
C,14.1315612467,-1.2345941019,-1.2229329637
C,13.4591626824,-2.285805 9878,-0.5274922631
H,13.5283341689,-4.2262216742,0.3823460485
H,14.787 7112486,-3.9495379448,-0.8391624526
C,13.3876949435,-0.1861232593,-1.7 715652428
C,12.078177062,-2.2300834945,-0.3923685356
C,11.996326303,-0 .1471150011,-1.641136666
H,13.9000591071,0.6123456316,-2.3002721734
C, 11.3398731485,-1.1678656506,-0.9438288119
H,11.5396024295,-3.010448232 ,0.1389427114
H,11.4455952698,0.6790365384,-2.0762643158
O,9.989290646 9,-1.2262960359,-0.7457734401
C,9.1702641638,-0.1796240734,-1.26094973 36
C,7.7379933693,-0.5142846744,-0.8694869272
H,9.2594957081,-0.121845 7169,-2.3537754123
H,9.4634527759,0.7868451805,-0.8304765487
H,7.65221 74111,-0.5770027115,0.2265383409
H,7.4499353031,-1.4897378029,-1.29163 55265
C,5.5384218721,0.3427829479,-1.0810791218
C,4.7825672312,1.51605 13352,-1.688097438
H,5.379370764,0.3163110616,0.0081058095
H,5.1673168 767,-0.6034932917,-1.5044062674
H,4.9386404556,1.5406326967,-2.7780324 999
H,5.1579446875,2.462355389,-1.2679260799
C,2.5829379827,2.37513194 81,-1.893346966
C,1.1506222158,2.0602131222,-1.486294654

H,2.659082144 5,2.4271898207,-2.9907655662
H,2.8837696627,3.352063353,-1.4834744077
H,1.0742722142,2.0084037109,-0.3890812273
H,0.8489919862,1.083913852,- 1.8964993171
C,-1.0481829261,2.9280755582,-1.6805276621
H,-1.196952715 7,2.9122608756,-0.5893683957
H,-1.4247274245,1.9753684822,-2.088135701 1
C,-1.8094984751,4.1032578356,-2.2961364532
N,-3.2398228103,4.0779398 4,-1.9582741276
H,-1.4105192339,5.0421853659,-1.9062350938
H,-1.671706 2424,4.1146948757,-3.3836634196
N,-3.968724137,3.2359960406,-2.5377115 439
N,-4.9930208106,2.6811926658,-2.440197528
O,-12.9761556187,-1.5361 314126,0.2480850119
O,-16.0138328265,-3.4553927485,0.4861015062
O,-18. 9137057897,-5.3698482891,-0.4589119178
O,6.9147797451,0.5187335352,-1. 3808869144
O,3.409014743,1.3418505881,-1.3821916731
O,0.3231109566,3.0 946099864,-1.9971891501

---

[1] The emission spectra of the lamps can be found on the Southern New England Ultraviolet Co. website: https://www.rayonet.org/shop.php?pline=lamps&compat=600.

[2] Brooks, K.; Yatvin, J.; McNitt, C.D.; Reese, R.A.; Jung, C.; Popik, V.V.; Locklin, J. “Multifunctional Surface Manipulation Using Orthogonal Click Chemistry”, Langmuir **2016**, *32*(26), 6600-6605; DOI: 10.1021/acs.langmuir.6b01591.

[3] Arnold, R.; McNitt, C.; Popik, V.; Locklin, J. “Direct Grafting of Poly(Pentafluorophenyl Acrylate) onto Oxides: Versatile Substrates for Reactive Microcapillary Printing and Self-Sorting Modification” *Chem Commun.*, **2014**, *50*(40), 5307-5309; DOI: 10.1039/C3CC47597B.

[4] Sun, P.; Tang, Q.; Wang, Z.; Zhao, Y.; Zhang, K., "Cyclic polymers based on UV-induced strain promoted azide-alkyne cycloaddition reaction", *Polym. Chem.* **2015,** *6* (22), 4096-4101.

[5] Sutton, D. A.; Yu, S.H.; Steet, R.; Popik, V.V. “Cyclopropenone-caged Sondheimer Diyne (Dibenzo[a,e]cy-clooctadiyne): A Photoactivatable Linchpin for Efficient SPAAC Crosslinking”, *Chem. Commun*. **2016**, *52* (3), 553-556; DOI: 10.1039/C5CC08106H.

[6] McNitt, C. D.; Popik, V.V. “Photochemical Generation of Oxa-Dibenzocyclooctyne (ODIBO), the most reactive acetylene for metal-free azide click ligation”, *Org. Biomol. Chem.* **2012**, *10* (41), 8200 – 8202; DOI: 10.1039/C2OB26581H.

[7] Sutton, D. A.; Popik, V.V. "Sequential photochemistry of dibenzo[a,e]dicyclopropa [c,g][8]annulene-1,6-dione: selective formation of didehydrodibenzo[a,e][8]annulenes with

ultrafast SPAAC reactivity", *J. Org. Chem.* **2016**, *81*(19), 8850-8857; DOI: 10.1021/acs.joc.6b01545.

[8] Nainar, S.; Kubota, M.; McNitt, C.; Tran, C.; Popik, V. V.; Spitale, R. C. "Temporal Labeling of Nascent RNA Using Photo-click Chemistry in Live Cells" *J. Am. Chem. Soc.* **2017**, *139*(24), 8090-8093, DOI: 10.1021/jacs.7b03121.

[9] Zhang, W.; Sun, Z.; Jiang, Y.; Liu, X.; Gupta, R.; Russell, T. P.; Bryan Coughlin, B. E. "Tuning microdomain spacing with light using ortho-nitrobenzyl-linked triblock copolymers", *J. Polym. Sci. Part B: Polym. Phys.*, **2018**, *56,* 355-361. https://doi.org/10.1002/polb.24565.

[10] Mortensen, M. R.; Nielsen, N. L.; Palmfeldt, J.; Gothelf, K. V. "Imidazole carbamate probes for affinity guided azide-transfer to metal-binding proteins", *Org. Biomol. Chem*., **2019**, *17*(6), 1379-1383; DOI: 10.1039/c8ob03017k.

[11] Frisch, M. J. et al. *Gaussian 16 Revision C.01*, Gaussian, Inc., Wallingford CT, 2019.

[12] Scott, A. P.; Radom, L. *J. Phys. Chem.* **1996**, *100*, 16502.

[13] Marenich, A.V.; Cramer, C.J.; Truhlar, D.G.; *J. Phys. Chem. B*, **2009**, *113*, 6378-6396.
